# The transcriptionally active regions in the genome of *Bacillus subtilis*

**DOI:** 10.1111/j.1365-2958.2009.06830.x

**Published:** 2009-08-26

**Authors:** Simon Rasmussen, Henrik Bjørn Nielsen, Hanne Jarmer

**Affiliations:** Center for Biological Sequence Analysis, Department of Systems Biology, Technical University of Denmark2800 Lyngby, Denmark

## Abstract

The majority of all genes have so far been identified and annotated systematically through *in silico* gene finding. Here we report the finding of 3662 strand-specific transcriptionally active regions (TARs) in the genome of *Bacillus subtilis* by the use of tiling arrays. We have measured the genome-wide expression during mid-exponential growth on rich (LB) and minimal (M9) medium. The identified TARs account for 77.3% of the genes as they are currently annotated and additionally we find 84 putative non-coding RNAs (ncRNAs) and 127 antisense transcripts. One ncRNA, *ncr22*, is predicted to act as a translational control on *cstA* and an antisense transcript was observed opposite the housekeeping sigma factor *sigA*. Through this work we have discovered a long conserved 3′ untranslated region (UTR) in a group of membrane-associated genes that is predicted to fold into a large and highly stable secondary structure. One of the genes having this tail is *efeN*, which encodes a target of the twin-arginine translocase (Tat) protein translocation system.

## Introduction

The bacterial genome is a highly compact structure. Both strands are densely covered by genes, of which a large part is organized into the even more gene-dense arrangements of operons. Recent technological advances have allowed for an empirical assessment of the prevalence of transcriptionally active regions (TARs) across an entire genome – by the use of either high-throughput sequencing of RNA-derived cDNA (Nagalakshmi *et al.*, 2008) or high-density oligo-nucleotide tiling arrays (Tjaden *et al.*, 2002; Bertone *et al*., 2004; David *et al*., 2006; Li *et al*., 2006; Reppas *et al*., 2006). Where the studies of Tjaden and co-workers and Reppas and co-workers investigated the transcriptional landscape of *Escherichia coli*, we report here the first findings of a high-density tiling-array study performed on the Gram-positive *Bacillus subtilis*. *B. subtilis* was first described in 1835 by the German scientist Christian Gottfried Ehrenberg as the hay/grass-associated bacterium, *Vibrio subtilis* (Ehrenberg, 1835). In 1872 another German scientist, Ferdinand Julius Cohn, renamed it *Bacillus subtilis* (Cohn, 1872). In 1876 Cohn showed for the first time that *B. subtilis* is capable of changing into an endospore state, and hereby surviving environmental changes not suitable for vegetative growth (Cohn, 1876). In 1930 the American bacteriologist, Harold Joel Conn, published a description of the Marburg strain of *B. subtilis* (American Type Culture Collection No. 6051) (Conn, 1930; Teas, 1949) and in 1947 this particular strain was subjected to both X-rays and UV light by Burkholder and Giles (Burkholder and Giles, 1947; Teas, 1949). Charles Yanofsky provided a number of stable auxotrophs, which had been isolated from these experiments, to John Spizizen (Spizizen, 1984), which studied their ability to develop natural competence (Spizizen, 1958; Zeigler *et al*., 2008). Further investigations resulted in the development of a highly efficient two-step protocol for transformation of the #168 strain (Anagnostopoulos and Spizizen, 1961), a success drawing the attention of the research community to such an extent that this strain was selected as the *B. subtilis* model strain. Today *B. subtilis* is widely used as an industrial production strain, and has even been shown to possess probiotic properties (Huang *et al*., 2008). And now, more than 10 years after fully sequencing and annotating the genome the first time (Kunst *et al*., 1997) and only shortly after the recent re-sequencing (Barbe *et al*., 2009), we experimentally validate and extend these efforts.

## Results and discussion

### Identification of transcriptionally active regions (TARs)

**Fig. 1:**
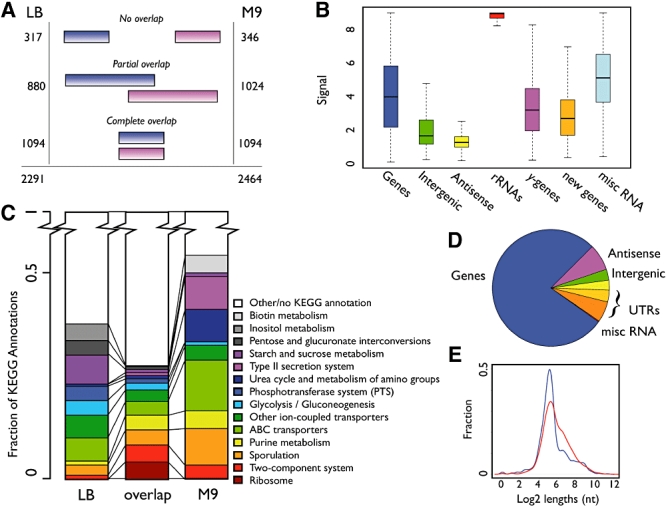
Expression in LB and M9. A. Diagram showing the overlap between TARs identified in the two media. No overlap: less than 5% overlap; Partial overlap: between 5% and 85% overlap (can overlap multiple TARs); Complete overlap: overlap of 85% or more. B. Box plot showing the log2-transformed signal range of the probes within annotated genes (non-y-genes), the regions between genes (Intergenic), antisense to known annotation, rRNAs, y-genes, new genes and misc RNAs as by the re-annotation by Barbe *et al*. C. Representation of the top 14 KEGG terms from genes uniquely expressed in LB and M9, and genes common to the two media. D. Pie chart illustrating the physical position of the probes returning a signal above background. Blue: ORF/gene; dark orange: 5′ UTR; orange: intergenic UTR; yellow: 3′ UTR; green: intergenic region (IR); magenta: antisense; red: misc RNA (Barbe *et al*., 2009). E. Density plot showing the log2 lengths (nt) of 5′ UTRs (red) and 3′ UTRs (blue) as they are determined in the study.

Hybridization of labelled RNA to densely tiled microarrays allows for a high-resolution mapping of genome-wide expression on both strands, and we have found that during growth in rich medium (LB) *B. subtilis* expresses 2291 transcriptionally active regions (TARs), whereas the corresponding number using minimal medium (M9) is 2464 TARs (all listed in Table S1). To determine how many of these were unique we have calculated the TAR overlap between the two conditions (see Fig. S1). If less than 5% of the TAR overlapped we define it as a unique TAR and likewise we define a common TAR if more than 85% overlap. This leads to the identification of 1094 common TARs, whereas 317 and 346 TARs are unique for LB and M9 respectively (Fig. 1A). In total 3662 non-redundant (overlap < 85%) TARs have been identified. An overview of the results in terms of identified genes, gene-like features and TARs can be seen in Table 1. Additionally we have annotated the TARs with experimentally verified and HMM predicted sigma factor binding sites and experimentally verified and predicted Rho-independent terminators (see *Experimental procedures*). A total of 10.5% and 27.3% of the TARs have been annotated with at least one experimental or predicted sigma factor binding site, respectively, and similarly 6.2% and 54% with an experimental or predicted Rho-independent terminator. Together the identified TARs account for 77.3% of the genes as they are currently annotated, and the overlap between the two media is 2843 genes corresponding to 64% of the 4422 known genes (Table S2). The whole-genome expression data, along with the predicted transcripts, sigma factor binding sites and Rho-independent terminators, are visualized in a figure spanning 48 pages (Fig. S2) and we encourage the reader to explore the findings.

**Table 1:** Overview of current annotation and the findings in this study.

Type	Current annotation^a^	LB	M9	Unique
Total CDS	4244	3189	3074	3420
Genes	1912	1514	1469	1627
y-genes	2332	1675	1605	1793
New genes	171^b^	106	103	119
rRNA	30	30	30	30
tRNA	86	82	83	83^c^
ncRNA	16^d^	50	68	84
Antisense	2^e^	60	99	127
TARs	–	2291	2464	3662^f^

a GenBank (AL009126.3).

b Genes annotated as new from Barbe *et al*. (2009).

c The missing tRNAs are: *trnD-Leu2*, *trnSL-Arg1* and *trnSL-Arg2*.

d Ando *et al*. (2002); Suzuma *et al*. (2002); Licht *et al*. (2005); Silvaggi *et al*. (2006); Gaballa *et al*. (2008); Saito *et al*. (2009).

e Silvaggi *et al*. (2005); Eiamphungporn and Helmann (2009).

f TARs were unique if the overlap was less than 85% between the two conditions.

Columns LB and M9 show number of occurrences in that particular category and Unique are unique occurrences in the two media combined.

**Fig. 3:**
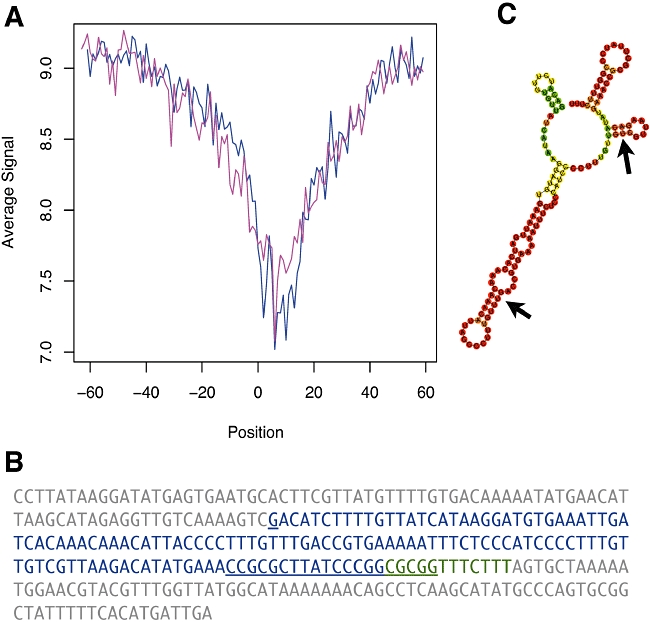
A. Average genomic DNA signal intensity over Kingsford predicted terminators (Kingsford *et al*., 2007). Position 0 corresponds to the middle nucleotide of the predicted terminator. Blue: Watson strand; magenta: Crick strand. B. The *ncr22* transcript and ∼100 nt upstream and downstream. Grey: intergenic nt; blue: identified transcript; underlined: transcription start site (+1) determined by 5′ RLM-RACE and stem-loop of terminator sequence; green: last part of terminator stem-loop and T-tail not within the identified transcript. C. Fold of *ncr22* transcript using RNAfold, coloured as base-pair probabilities. Blue equals zero and red equals 1. The two arrows indicate binding sequence to *cstA* transcript upstream of the start codon.

Hybridization of labelled genomic DNA (gDNA) to the tiling array results in a uniform signal level throughout the genome (as can be seen in Fig. S2). However, we found that low gDNA signals coincide with experimentally verified and predicted Rho-independent terminators (Fig. 3A). This may be explained by the formation of stable structures possibly forming in both the probe and the target, which hereby prevents detection (Ratushna *et al*., 2005). These findings may also explain why normalization using gDNA hybridizations, as performed by Huber *et al.*, did not improve the performance of our TAR findings (data not shown) (Huber *et al*., 2006). This normalization are in areas with Rho-independent termination introducing significant noise and interferes with the determination of correct transcript boundary.

We have benchmarked the prediction of TARs against gene coverage, known transcription start sites (TSSs) and signal autocorrelation (see Fig. S3). From this we see that of 2500 genes predicted to be covered by TARs, only ∼2.5% are estimated to be false positives, here defined as TARs covering genes expressed at the opposite strand (not taking possible antisense transcripts into account). Regarding TSS, our findings are in general within 20 nt from the experimentally verified starts. Additionally it is interesting to note that we do observe a spatial gene expression dependence – neighbouring genes tend to be coexpressed in operons. Experimentally we verify the TSSs of five of the determined transcripts using RNA ligase-mediated rapid amplification of cDNA ends (RLM-RACE) and the results are summarized in Table S3. The verified transcript start sites are within 30 nt of our findings.

### Comparison in gene utilization using two different growth sources

The distribution of the most common KEGG annotations (Kanehisa *et al*., 2008) are shown for genes expressed in both media (common) compared with genes expressed uniquely to either of the conditions. As expected, it becomes evident that *B. subtilis* utilizes different pathways when growing in the two different media. One example is the difference in the *Glycolysis/gluconeogenesis*, where a closer inspection reveals that the gluconeogenesis is inactive when the cell is growing in minimal medium, which is expected (Fillinger *et al*., 2000). Likewise, a large portion of genes involved in the development of competence (with the KEGG annotation *Type II secretion*) is active when the cell is starving. Whereas, many of the genes exclusively expressed when the cell is growing in the rich medium include a large proportion of genes encoding products responsible for uptake and metabolism of various carbon sources, which is expected from growth in a complex medium (Deutscher *et al*., 2002). A puzzling observation is that sporulation genes, based on KEGG annotation (Fig. 1C), are seen expressed at conditions when sporulation should not occur. However when investigated in detail it becomes clear that the majority of these are expressed at levels close to our detection limit and that the few highly expressed are sporulation initiation control genes such as response regulator aspartate phosphatase genes/operons (Auchtung *et al*., 2006). To further ensure that sporulation is indeed not occurring we have analysed the expression of the sporulation regulons σ^F^, σ^E^, σ^G^ and σ^K^ (Steil *et al*., 2005) and reassuringly we find that all of these are expressed below background (shown in Fig. S4). We therefore contribute the above phenomenon to genes involved in sporulation control and/or genes with divergent functionality.

### Determination of untranslated regions (UTRs)

Forty per cent of the probes tiling the genome give a signal above background. As is shown in Fig. 1D the majority of these seemingly expressed elements are generally localized within regions expected to give a signal, either within an annotated gene or in the putative untranslated regions (UTRs) – as they have been determined in this study. The majority of the probes located in the intergenic regions (IRs) and the antisense regions (ARs) have signals below background level. Additionally 5.6% of the probes with signal above background fall within putative 5′ UTRs as they are determined in this study, whereas probes in the 3′ UTRs only comprise 2.7% of the expressed probes, which is even fewer than for intergenic UTRs (3.2%). This corresponds to 1648/1633 (LB/M9) transcripts with a defined 5′ UTR and 1371/1506 (LB/M9) with defined 3′ UTRs. The majority of this difference between 5′ and 3′ UTRs may be explained by their difference in length, as is shown in Fig. 1E. The median lengths are 47 and 36 nt for 5′ and 3′ UTRs respectively (significant in a two-sided Wilcoxon rank sum test with a *P*-value of 1 × 10^−24^). This is opposite to what is observed in higher organisms, such as in the study of David *et al*. (2006) in *Saccharomyces cerevisiae*, where the 3′ UTRs are found to be longer than the 5′ UTRs. It does however correspond well to the previous discovery that the average length of the 3′ UTRs is increasing as a function of the organismal complexity (Mazumder *et al*., 2003). These findings point at emphasis on 5′ UTRs or lack thereof on 3′ UTRs compared with higher organisms in transcriptional and post-transcriptional control in *B. subtilis.* Already well-studied examples of such 5′ UTR-mediated control in *B. subtilis* is the control of the tryptophan operon and *S*-adenosyl methionine (SAM) riboswitch (Grundy and Henkin, 1998; Gollnick *et al*., 2005).

### Novel protein-coding genes

The new annotation by Barbe *et al.* has identified 171 putative novel protein-coding genes increasing the amount of protein-coding genes in *B. subtilis* to 4244 and here we report the first expression data covering these. In general the novel protein-coding genes are expressed at lower signals compared with the remaining protein-coding genes with expression means of 3.0 and 3.9 respectively (Fig. 1B). Additionally only 70% are found to be expressed above background signal, which is less than the protein-coding genes in general (77%). This combined with the short lengths of the newly annotated genes (median 159 versus 258 aa for remaining) and the fact that some of these were found to have sequence errors explains why these have not been annotated before.

We have investigated whether the novel protein-coding genes are expressed mono- or polycistronic and we find that 26 of the 119 expressed genes are encoded monocistronic, which may provide experimental evidence for the existence of these genes. The novel protein-coding genes are listed in Table S4 together with their expression values and the genes annotated to the TAR they belong to.

**Fig. 2:**
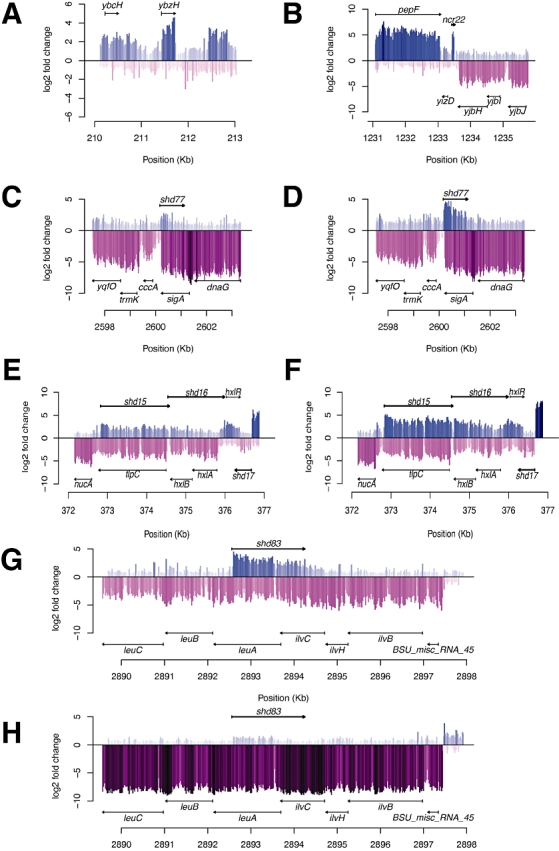
Expression of different regions of the *B. subtilis* genome during growth, where the position and direction of genes are indicated with arrows. Expression on the Watson strand is blue, Crick strand is magenta and the colour intensity also indicates signal strength. A. Expression in the 210–212 kb region in LB showing new protein-coding gene *ybzH* expressed monocistronic. B. Expression during growth in LB medium in the region 1231–1236 kb, showing expression of the novel non-coding RNA *ncr22*. C. Antisense expression in the region 2598–2603 kb in LB (*shd77*) of *sigA*. D. As (C), but expression in M9. E. Antisense expression in the region 372–377 kb in LB (*shd15-shd17*) of *tlpC, hxlB, hxlA* and *hxlR*. F. As (E), but expression in M9. G. Antisense expression in the region 2890–2898 kb during growth in LB (*shd83*) of the operon *ilvBHC-leuABC*. H. As (G), but expression in M9.

An interesting monocistronic expressed new gene is *ybzH*, within the *pro1* prophage-like element (see Fig. 2A), positioned on a transcript with clearly defined boundaries. RLM-RACE mapped the TSS to 7 nt downstream of our observed boundary and exactly at a predicted +1 of SigA factor binding site. Additionally, a Rho-independent terminator is predicted at the transcript end. Regarding functionality, Barbe *et al.* reported blast hits with high similarity to proteins of the arsenic resistance transcriptional regulator family (ArsR) from different *Bacilli* and *Geobacilli* species. This is in agreement with findings that prophages have been shown to confer protective traits to heavy metals such as arsenic (Cervantes *et al*., 1994).

### Alternative ORFs as the result of TAR identification

The transcriptional map also uncovers irregularities in the current annotation, e.g. the region containing the annotated translation start site or stop codon is not expressed. The discrepancy in the annotations of these genes might be due to sequence errors at the time of annotation; however, re-sequencing and re-annotation efforts of Barbe *et al.* seem to have corrected several of these irregularities. An example is the *ykvS* gene that was re-annotated from 143 to 62 aa and is now confined within the observed transcript (see Fig. S2, at 1447 kb). Examples of irregularities between gene annotation and TARs are *cgeD*, *ybcL*, *ybcM*, *ycgN*, *yxxF*, *yqjD* and *ydbO* (Fig. S2). However irregularities may also be explained by alternative internal promoters. The latter is the case of the *hisC*, *tyrA* and *aroE* operon which is transcribed from a promoter residing inside the *trpA* gene (see Fig. S2, at 2372 kb) (Gollnick *et al*., 2005).

### Expression of prophage elements

The data generated here are well suited for a systematic investigation of the prophage elements in *B. subtilis* and we have examined the expression of the prophage elements *PBSX*, *SPβ* and *skin*, and the prophage-like elements *pro1–7* (Zahler *et al*., 1977; Wood *et al*., 1990; Takemaru *et al*., 1995; Nicolas *et al*., 2002). The functionality of the genes expressed from the prophage elements during exponential growth would be expected to be involved in control of the bistable lysogenic equilibrium, conferring immunity to the phages or to be functional genes obtained via hitch-hiking. Genes with unknown functions that are expressed during exponential growth from within these elements are then likely not to be inducing the lytic cycle, but confer beneficial traits during growth in the natural habitat (Lazarevic *et al*., 1999).

In previous microarray studies large clusters of genes in prophage elements were found to be expressed at low levels (Helmann *et al*., 2001). Our analysis reveal that this is in particular true for the *skin* element and to some degree for *pro2* and *pro7* (Figs S5 and S6). Characteristic of these clusters is that their expression are at extremely low levels, indicating that even low expression of these genes is undesirable during exponential growth. This trend is not observed to the same extent within the *SPβ* prophage, where there are low, but not non-existing, basal gene expression levels. Additionally the sublancin genes and neighbouring area (*bdbB* to *sunI*) are highly expressed within *SPβ* and exemplify that prophage genes may confer beneficial traits that are not essential. *yolA* in *SPβ* is the highest expressed gene within the prophage elements and is one of the highest expressed in the entire genome (above the 98% quantile). The gene encodes a 155 aa protein predicted to contain a signal peptide and is hence a putatively exported protein.

The prophage and prophage-like elements *PBSX*, *pro3*, *pro4* and to some extent *pro5* show high levels of gene expression. For the *PBSX* element it is in agreement with previous observations (Krogh *et al*., 1996) and coincides with the fact that it has similar base composition to the native *B. subtilis* sequence in contrast to typical AT-rich prophage elements (Nicolas *et al*., 2002). These expression profiles indicate limited phage functionality or viability of *PBSX*, *pro3*, *pro4* and *pro5*, whereas *skin*, *pro1* and *pro7* may contain gene products undesirable during exponential growth. Expression of all genes, including prophage and prophage-like elements, are listed in Table S2.

### Identification of novel non-coding RNAs

In order to extract high-confidence new non-coding RNAs we have set-up a list of criteria that should be fulfilled. In total we extract 84 non-coding RNAs from segments that fulfil the following criteria: (i) no annotated transcription according to the latest GenBank version (AL009126.3), (ii) higher signal level than neighbouring segments, (iii) higher signal than the corresponding antisense region, (iv) maximum 5% of the probes cross-hybridize to other regions of the genome (using a blat-scoring scheme; Kent, 2002), and (v) if shorter than five probes, the signal should be observed in both media. These putative non-coding RNAs (ncRNAs) (*ncr1–84*) are listed in Table S5. They have a median length of 197 nt and range from 55 to 571 nt. From *E. coli* it is known that the functions of ncRNAs cover a wide range (Kawano *et al*., 2005). Figure S7 shows how conserved the *ncr* genes are across species. We annotate 65% (55) of the *ncr*s with experimental or predicted sigma factor binding sites and 70% (59) with an experimental or predicted Rho-independent terminator.

Of the 16 already known ncRNAs in *B. subtilis*, other than rRNAs and tRNAs, we identify 10: *surA*, *ssrSB*, *ssrSA*, *bsrF*, *bsrG*, *bsrH*, *bsrI*, *fsrA*, *scr* and *ssrA* (Ando *et al*., 2002; Suzuma *et al*., 2002; Silvaggi *et al*., 2006; Gaballa *et al*., 2008; Saito *et al*., 2009). The remaining ncRNAs *bsrC*, *bsrD*, *bsrE*, *surC*, *SR1* and *polC-ylxS* are not identified in our study (Licht *et al*., 2005; Silvaggi *et al*., 2006; Saito *et al*., 2009). Even though we do not identify the *bsrE* transcript, we do find *ncr40* expressed from the opposite strand at the same genomic location (Saito *et al*., 2009). However as there are expression from both strands in this region *ncr40* may be an antisense transcript of *bsrE*. Reasons for the absence of the other RNAs might for *surC* and *polC-ylxS* be that they were identified as being expressed under sporulating conditions (Silvaggi *et al*., 2006). Regarding the *bsrC* and the *SR1* transcripts the regions are expressed (*ydaG-ydaH* and *slp-speA* respectively); however, segments are not identified. The *bsrC* segment is joined with the upstream gene and *SR1* is weakly expressed and is therefore not identified as a segment in our analysis; however, visual inspection reveals a possible transcript at the position (Licht *et al*., 2005). *bsrD* is actually well defined in M9; however, it fails to meet the criteria as it is only two probes and not present in LB (Saito *et al*., 2009). In addition to these non-coding RNAs, 22 riboswitches such as purine, SAM, TPP, FMN, glycine and lysine, and T-box elements are identified as *ncr* elements (Barbe *et al*., 2009). This leaves 54 non-coding RNA elements not previously described.

An example of a novel putative non-coding transcript is *ncr22*, which is located between *yizD* and *yjbH* and is a clearly defined transcript showing high expression in both media (Fig. 2B). Using 5′ RLM-RACE we map the TSS to 18 nt upstream of the observed boundary; however, we are not able to identify a probable sigma factor binding site (Fig. 3B). A Rho-independent terminator sequence is positioned in the 3′ of the transcript where the stem-loop is folded from the last 16 nt of the transcript and 5 nt outside the 3′, and the T-tail following these. This results in a transcript of 133 nt, which when folded using RNAfold seems to fold into a stable structure with a minimum free energy (MFE) of −33.8 kcal mol^−1^. Additionally the *ncr22* transcript is highly conserved in the *Bacillus*, *Geobacillus* and *Staphyloccocus* genera providing supporting evidence for this transcript (Fig. S7). As most bacterial ncRNAs act in a *trans*-acting regulatory role, we have searched for mRNA targets for *ncr22* using targetRNA (Tjaden *et al*., 2006; Vogel and Wagner, 2007). Interestingly the best hit is in the 5′ of the carbon starvation-induced protein messenger (*cstA*). The interaction between the two RNAs occur from +13 to −22 (relative to the start codon) in the *cstA* transcript, covering the region containing the Shine–Dalgarno (SD) sequence, and nucleotides 61–95 in *ncr22* (Fig. 3B). In *E. coli cstA* is under translational control of the RNA-binding protein CsrA and the sRNAs CsrB and CsrC (Dubey *et al*., 2003); however, the CsrA homologue in *B. subtilis* does not seem to have binding affinity for the *cstA* transcript (similarity search using CsrA-binding domains; Yakhnin *et al*., 2007). The above suggests that *cstA* may be under translational control in *B. subtilis* not by CsrA but possibly by *ncr22*.

### Identification of antisense RNAs

We identify 127 TARs fulfilling the same criteria as for non-coding RNAs, except that they are expressed antisense to already known genes with an overlap of more than 10%. We term these shadow genes and name the TARs *shd1–shd127*; details on these are listed in Table S6. The median length of shadow expressed transcripts is 681 nt and ranges from 197 to 3516 nt.

A possible function of these antisense transcripts is as *cis*-acting regulators, as described by Eiamphungporn and Helmann (2009) for the *yabE* gene and Silvaggi *et al*. (2005) for *yqdB*. In this study we detect antisense expression to *yabE* (*shd4*) during growth in both media and in addition an antisense signal (*shd3*) for the upstream gene *yabD* (Fig. S2, at 49 kb). Likewise we observe the other known *B. subtilis* antisense transcript *ratA* (*shd80*) expressed in both media as antisense to *yqdB* (Silvaggi *et al*., 2005).

As an example of a novel antisense transcript *shd77* should be mentioned since this could potentially be of significant importance as it is expressed antisense to *sigA*, the principal sigma factor during vegetative growth (Haldenwang, 1995) (Fig. 2C and D). Sigma A and E binding sites are predicted at −10 and +10, respectively, of the observed 5′-TAR boundary. This finding adds to the complexity of the regulation of the *yqxD-dnaG-sigA* operon, which is already known to be controlled via at least seven different promoters (Wang *et al*., 1999). Furthermore, we have experimentally verified the TSS of *shd15* (Fig. 2E and Fand Table S3) and found it to correspond to the TAR TSS prediction and identify a putative SigA site with −35: TTGATT and −10: TATGAT. This transcript appears to be one of three antisense transcripts (*shd15–17*) antisense to *tlpC*, a methyl-accepting chemotaxis protein, *hxlAB*, formaldehyde detoxification system and *hxlR*, which encodes a positive regulator of *hxlAB*.

If antisense transcripts are acting as negative *cis*-regulatory elements the signal levels of sense and antisense ratio would be expected to anticorrelate which should be possible to observe if there are differential regulation between the conditions tested. Generally, and in line with expectations, we do see anticorrelation (Pearson correlation −0.22) when comparing antisense and sense ratios (LB versus M9) (Fig. S8). When investigating this for *anti-yabE* (*shd4*) the antisense transcript level increases 1.1 log2-fold (LB to M9) with a concomitant 2.7 log2-fold decline in the sense *yabE* signal. However, this trend may not always be observed if multiple regulatory mechanisms control the sense expression or the area is not differentially expressed, as exemplified by the antitoxin *ratA* (*shd80*) with the log2 antisense and sense ratios of −0.9 and −1.1. The antisense–sense transcript pair with the strongest change observed when comparing LB with M9 medium is *shd83*, which partially overlaps *leuA* and *ilvC* in the *ilvBHC-leuABC* operon (Fig. 2G and H). The products of the operon are enzymes involved in branched chain amino acid synthesis and the full-length mRNA is subjected to transcriptional regulation by tRNA^Leu^ T-box in the 5′ UTR, CodY, CcpA, TnrA and processed into smaller units (Mäder *et al*., 2004; Shivers and Sonenshein, 2005). Due to the many regulatory modes of the *ilvB* operon further experiments are needed to understand whether the observed expression change can be explained by antisense RNA expression.

The fraction of sense coding sequence covered by antisense transcripts seems to be divided in two distributions, transcripts covering close to or full length of genes and another existing of transcripts only partially covering genes (Fig. S8). The groups are exemplified by the two already known antisense transcripts *anti-yabE* and *ratA*, which are predicted to cover 72–80% and ∼35% of the coding sequences respectively (see Fig. S2, at 49 and 2678 kb, and Table S6; Silvaggi *et al*., 2005; Eiamphungporn and Helmann, 2009).

In addition to this some of the antisense transcripts seem to be UTRs that overlap genes on the opposing strand. We annotate eight of the transcripts as putative overlapping 5′ UTRs and 26 as putative 3′ UTRs. A closer inspection of the latter reveals that 35% of these have start sites in a 50 nt range of an experimental or predicted Rho-independent terminator (for such an example see *shd49*, Fig. S2 at 1261 kb). This suggests that some 3′ UTR antisense transcripts may arise from terminator read-through events.

We predict sigma factor binding sites for 42% (50% when leaving out putative overlapping 3′ UTRs) of the antisense transcripts near the observed 5′ TSSs. Furthermore, only 17% (22) of the antisense transcripts were predicted to have an Rho-independent terminator at the 3′, which is significantly lower than what is observed for the identified non-coding RNAs (70%).

As Xu *et al*. (2009) report bi-directional promoters as a source of antisense transcription in *S. cerevisiae*, we investigated whether such a phenomenon could also explain some of the antisense transcription in *B. subtilis*. We identified putative sigma factor binding sites on the opposite strand of the predicted antisense TSS and in the case of 16 (13%) antisense TSSs a predicted or experimental site was identified. These findings point at antisense transcription in *B. subtilis* as a ‘directed’ effort and perhaps to a lesser extent the result of bi-directional promoters.

### Sequence and structurally conserved 3′ UTRs

**Fig. 4:**
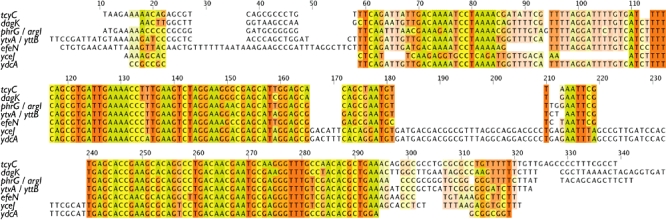
Multiple alignment in clustalw2 (Larkin *et al*., 2007) of the 250 nucleotides downstream of *tcyC*, *dagK*, *phrG* (shared with *argI)*, *ytvA* (shared with *yttB)*, *efeN*, *yceJ* and *ydcA. efeN*, *yceJ* and *ydcA* have the reverse complement of the sequence and are here aligned using the reverse complement. Bases are coloured A = green, T = red, C = yellow, G = orange and the intensity at each position indicates base conservation. No conservation is uncoloured.

**Fig. 5:**
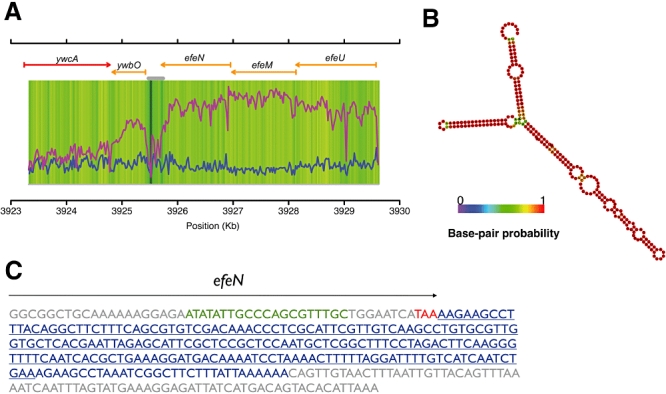
A. Expression of the genomic area near *efeN* as an example of the identified conserved, stable structure forming 3′ UTRs. Watson strand is blue, Crick strand is magenta and the results from the DNA hybridization are shown in a colour gradient from dark green (low signal) to yellow (high signal). The grey bar indicated the location of the 3′ UTR transcript. B. RNA structure, folded using RNAfold, of 220 nt downstream of stop codon of *efenN* coloured as base-pair probabilities. Blue equals zero and red equals 1. C. 3′ sequence of *efeN* containing transcript. Grey: *efeN* CDS, and intergenic nt; green: RLM-RACE primer; red: *efeN* stop codon; blue: 3′ UTR identified by RLM-RACE and the sequence folded in (B); underlined: the conserved sequence.

During the extraction of non-coding RNAs 39 putative *ncr*s were excluded, due to cross-hybridizing probes within the transcripts. An investigation of these revealed that a group of genes have a long 3′ UTR (∼220 nt) with high sequence similarity and according to RNAfold a highly stable secondary structure (see Fig. 4 and Fig. S9). The latter will in our data be apparent by a local decline in signal in both RNA and DNA hybridizations, hence causing the TAR to be split up into a gene containing TAR and a downstream ncr-like TAR. The nine genes having these conserved 3′ UTRs are listed in Table 2 along with their function (known/predicted). A closer inspection reveals that most of these are somehow membrane-associated, either physical sitting in the membrane, in complex with a membrane protein, or being exported. One possible exception is *ytvA*, which is a blue-light-sensing protein positively regulating the sigma-B pathway. Figure 5A shows RNA and DNA hybridization of a ncr-like TARs downstream of *efeN* (former *ywbN*) together with the predicted structure (Fig. 5B). Experimentally we mapped the *efeN* 3′ UTR using 3′ RLM-RACE to 225 nucleotides downstream of the *efeN* stop codon, hereby showing that the conserved sequence is indeed part of the transcript (Fig. 5C).

**Table 2:** The nine genes with the conserved 3′ UTR and their function/predicted function.

Gene	Protein function/genetic organization	Reference
*dagK*	Essential diacylglycerol kinase, lipoteichoic acid (LTA) production	Jerga *et al*. (2007)
*tcyC*	Part of the *tcyABC* operon encoding an l-cysteine uptake system	Burguière *et al*. (2004)
*ydcA*	Rhomboid-like membrane protein^a^	–
*yceJ*	Similar to multidrug-efflux transporter^a^	–
*phrG*	Phosphatase (RapG) regulator, exported, divergent of *argI*	Ogura *et al*. (2003)
*argI*	Arginase, part of *rocDE-argI* operon (RocE: arginine permease)	Gardan *et al*. (1995)
*yttB*	Similar to multidrug resistance protein,^a^ divergent of *ytvA*	–
*ytvA*	Blue-light sensor, positive regulator of the sigma-B pathway	Gaidenko *et al*. (2006)
*efeN*	Similar to Dyp-type peroxidases,^a^ Tat-translocated protein	Jongbloed *et al*. (2004)

a blast search result.

EfeN is a substrate of the twin-arginine translocase (Tat) protein translocation system and is expressed as a part of the *efeUMN* operon. The operon has been shown to be regulated by Fur (ferric uptake regulator) and EfeN is predicted to function as a Fe(III) permease of the dye-decolorizing Dyp-peroxidase family (Jongbloed *et al*., 2004; Ollinger *et al*., 2006). Interestingly the Tat system is able to transport folded proteins and proteins with bound cofactors and to some extent only correctly folded proteins are transported (DeLisa *et al*., 2003). As previous studies on EfeN in *B. subtilis* have focused on the Tat signal peptide the expression of *efeN* coding sequence has been performed via a *xylA-efeN-myc* cassette, without the conserved 3′ UTR (Jongbloed *et al*., 2004). From this it has been observed that while EfeN expressed from the *xylA-efeN-myc* cassette has been identified in extracellular extracts, the wild-type EfeN has never been detected either inside or outside the cell (H. Antelmann and J.M. van Dijl, pers. comm.). To this respect we, in these expression data, see that during vegetative growth, *efeU*, *efeM* and *efeN* are expressed at high rates (Table S2). From this we speculate that the 3′ UTR of the *efeN* transcript may have a function in regulating the translation and/or the physical location of EfeN. Upon completion of folding or cofactor binding the protein would be available for translocation or insertion into the membrane. In *E. coli* Tat proofreading exists, where a protein binds and hereby blocks the Tat signal peptide, so that it is shielded from the translocase until proper assembly has been completed (DeLisa *et al*., 2003). Examples of these Tat signal binding peptides in *E*. *coli* are TorA and NapD (Maillard *et al*., 2007), of which no homologues are found in the *Bacilli*.

Another possible function of the 3′ UTRs could be to inhibit 3′ directed RNA degradation as double-stranded RNA and stable helical regions have been shown to block the activity of the major 3′ exoribonuclease in *B. subtilis* PNPase and RNase II. Additionally the 3′ exoribonuclease RNase R, which has been shown to be able to degrade double-stranded RNA and RNA with secondary structures, needs a single-stranded RNA tail to be active. It has been reported to be active with single-stranded tails of more than 40 nt, and was demonstrated not to be active on RNA with only a 12 nt single-stranded tail (Oussenko *et al*., 2005). The single-stranded tail of the conserved 3′ UTRs ranges from 2 to 10 nt for *phrG* and *efeN*, respectively, meaning that they may be protected from 3′ exoribonuclease degradation (Fig. 5and Fig. S9).

## Conclusions

Since these findings are based on the first experimental attempt to map expression on a genome-wide scale in *B. subtilis*, they are to a large extent allowing us to refine our knowledge about the *B. subtilis* transcriptome. But since almost 1:5 of the currently annotated protein-coding genes are not expressed in this study more studies using a large spectrum of different growth conditions and perturbations are needed in order to reveal the full transcriptional map of *B. subtilis*.

As this is, taken the above into consideration, a work in progress, we report the expression map of *B. subtilis* in two different media, LB and M9, as 2291 and 2464 TARs, respectively, which in total adds to 3662 non-redundant TARs. The predicted TARs clearly describe the spatial expression patterns expected from genes expressed from operons as is the case in *B. subtilis*. Additionally, and as expected, a significant difference has been observed between the length of 5′ and 3′ UTRs with medians of 47 and 36 nt respectively.

By the use of KEGG annotation we clearly see expected differences in gene expression when comparing the two growth sources. Regarding the novel protein-coding genes predicted in the re-sequencing project of Barbe and co-workers, we here report them to be expressed at low levels compared with the previously annotated protein-coding genes although 70% (119) of them are expressed above background levels. Additionally 26 of these are found to be expressed on monocistronic transcripts, providing experimental evidence for their existence. The TSS of *yzbH* has here been mapped using 5′ RLM-RACE. Furthermore the annotation of seven genes did not match well with the expression signals seen, suggesting re-annotation of these.

An analysis was also performed on prophage and prophage-like elements, revealing large clusters of genes from the *skin* element, *pro2* and *pro7*, that are not expressed. On the contrary *PBSX*, *pro3*, *pro4* and *pro5* show high expression and we identify an uncharacterized putative exported protein, *yolA*, within the *SPβ* prophage to be among the most abundantly expressed genes on the genome.

We discover a range of high-confidence novel features covering 84 non-coding RNAs and 127 antisense transcripts. We identify 10 out of the 16 known ncRNAs known in *B. subtilis* (excluding tRNAs and rRNAs), a putative ncRNA on the opposite strand of *bsrE* and 22 riboswitches. Additionally the 5′ of *ncr22* was mapped using RLM-RACE and it may act as a putative *trans*-acting inhibitor on translation of the carbon starvation protein gene *cstA*. Regarding the antisense transcripts, 27% of them could be overlapping 5′ or 3′ UTRs and 50% of the non-3′ UTR antisense transcripts have predicted sigma factor binding sites near the observed TSS. The TSSs of 16 antisense transcripts are opposing an experimental or predicted sigma factor binding site and may be products of bi-directional promoters. The expression of antisense transcripts was found to be anticorrelated to their sense counterparts.

In addition, the analysis of gDNA hybridization has led us to discover stable structures in the 3′ UTRs of several transcripts and one of these tails was experimentally verified for EfeN, a Tat-translocated protein.

## Experimental procedures

### Design of the tiling array BaSysBio Bsub T1

A total of 385 000 feature NimbleGen arrays have been designed, using OligoWiz 2.0 (Wernersson and Nielsen, 2005), with long iso-thermal probes (45–65 nt) covering the entire genome of *B. subtilis* #168 Trp^+^ (AL009126.2) in 22 nt intervals on each strand and an 11 nt offset between the strands. The microarray design and data are available at the Gene Expression Omnibus (GEO) database at NIH as ‘*BaSysBio Bacillus subtilis T1385K array version 1*’ with the records GPL8486 and GSE16086 respectively. The data were remapped to the re-sequenced genome (AL009126.3) using blat (blast-like alignment tool) and 383 probes were removed due to low match (Kent, 2002; Barbe *et al*., 2009).

### The bacterial strain, growth conditions and sample processing

Three *B. subtilis* #168 Trp^-^ cultures were grown in LB medium and three in M9 medium at 37°C and 120 r.p.m. until the OD_600_ had reached a value of 0.5. Generation times for *B. subtilis* in the experiments were 26 and 78 min respectively. Media compositions were: LB (Sigma-Aldrich): 10 g l^−1^ Tryptone, 5 g l^−1^ yeast extract and 5 g l^−1^ NaCl; M9: 0.3% glucose, 0.1 mM CaCl_2_, 1 mM MgSO_4_, 0.05 mM FeCl_3_, 8.5 g l^−1^ Na_2_HPO_4_·2H_2_O, 3 g l^−1^ KH_2_PO_4_, 1 g l^−1^ NH_4_Cl, 0.5 g l^−1^ NaCl, 1 mg l^−1^ MnCl_2_, 1.7 mg l^−1^ ZnCl_2_, 0.43 mg l^−1^ CuCl_2_·2H_2_O, 0.6 mg l^−1^ CoCl_2_·6H_2_O and 0.6 mg l^−1^ Na_2_MoO_4_·2H_2_O. A total of 25 ml from each culture was transferred to a 40 ml tube 1/3-filled with crushed ice and spun at 7000 r.p.m. for 5 min, after which the supernatant was discarded and the cell pellet frozen by dumping the closed tube into liquid nitrogen. Total RNA was extracted by the use of the *FastRNA PRO Blue Kit* from Qbiogene as recommended by the supplier, but with an additional shake in the *FastPrep* instrument and a 1 min incubation on ice between the two shakings. DNA was extracted (from four independent cultures grown in LB under the same conditions as described above) using the *DNeasy Blood tissue kit* from Qiagen as recommended by the supplier. Both RNA and DNA were send to NimbleGen labelled and hybridized to the BaSysBio Bsub T1 chip using a protocol for strand-specific hybridization developed during this work (the *BaSysBio* protocol), and the NimbleGen-standard protocol for double-stranded DNA respectively. All samples were labelled with Cy3 and in the case of RNA first-strand cDNA was produced by random priming and Actinomycin D inhibition of the reverse transcriptase polymerase effect (as suggested by Perocchi *et al*., 2007). We found that the optimal enzyme concentration was 40 μg μl^−1^.

### RNA ligase-mediated rapid amplification of cDNA ends (RLM-RACE)

Transcription start sites were mapped for five transcripts using FirstChoice® RLM-RACE Kit (Ambion) following the manufacturer's protocol. DNase-treated RNA from an independent LB experiment was used as template and nested PCR was performed using primers listed in Table S3. Single-band PCRs were purified using Qiaquick PCR Purification Kit (Qiagen) and multiple bands were excised from gels and purified using Qiaquick Gel Extraction Kit (Qiagen) and sequenced. Transcript end mapping was performed for *efeN* by poly-adenylating DNase-treated RNA using Poly(A) Polymerase (Epicentre Biotechnologies) and Firstchoice® RLM-RACE kit (Ambion) following manufacturer's protocol. Only a single PCR was needed for the 3′ RLM-RACE and the primers are listed in Table S3.

### Data preprocessing, segmentation and TAR creation

Segmentation was performed using the Structural Change Model (SCM) described by Huber *et al*. (2006), in the *Bioconducter* package *tilingArray*. We used default settings allowing 3000 segment to be created for each strand with a maximum length of 400 probes (∼8800 bp). Normalization by reference (gDNA data) was not used as it according to our benchmarking decreased performance, and the optimal detection limit (background) was determined to the 60% quantile (2.2 log2 signal) of the signal intensities. Following the segmentation we created the resulting TARs by accepting all segments above background and joining neighbouring segments if the five probes on each side of a breakpoint were all above background, and when a Student's *t*-test did not rejected the hypothesis that these two sets of probes belonged to the same signal-intensity distribution (*P*-value > 1e-10). Finally short TARs (< 5 probes) between two highly expressed segments were removed. The resulting list of TARs is shown in Table S1.

### Assessment of breakpoints

To determine the accuracy of the TAR breakpoint predictions these were benchmarked against the 654 experimentally verified TSS, which were extracted from the DataBase of Transcriptional regulation in *B. subtilis* (DBTBS, release 5) (Sierro *et al*., 2008), and 425 experimentally verified Rho-independent terminators (Hoon *et al*., 2005). Both the sigma factor binding sites and Rho-independent terminator annotation was transferred to the re-sequenced genome (AL000926.3) using blat (Kent, 2002). The TAR-signal ends were adjusted 9 and 51 nucleotides downstream to optimally predict TSS and TES. The belonging receiver operating characteristic (ROC) curves (Swets, 1988) are shown in Fig. S3.

### Annotation of TARs and UTRs

Known genes were annotated to the TAR with the maximal overlap to it, and only if more than 50% of the gene was covered by the given TAR. The reported 5′ UTR lengths are the distances from the 5′ end of the given TAR to the start of the first ORF in the TAR (if any) and likewise the 3′ UTR lengths are the distances from the stop codon of the last ORF to the TAR 3′ end. Internal UTRs were calculated as the distance between stop and start for two neighbouring ORFs inside TARs.

### Sigma factor and terminator predictions

All identified transcripts were annotated with experimentally verified sigma factor binding sites and Rho-independent terminator sequences (Hoon *et al*., 2005; Sierro *et al*., 2008). The co-ordinates of the above were transferred to the re-sequenced genome (AL009126.3) using blat. Additionally the transcripts were also annotated with predicted sigma factor binding sites from two sources, sigma A sites from Jarmer and co-workers and sigma A, B, E, D, G, F, K, H, X, W predicted by a HMMbuild from all known alignments from DBTBS (Release 5) (Jarmer *et al*., 2001; Sierro *et al*., 2008). The HMM was created using HMMbuild and HMMcalibrate and was used by HMMsearch to search in the sequences 100 nt upstream and 50 nt downstream the TSS. The sigma factors I, M, Y, Z and YlaC and YvrI had too few known sites to build HMMs. Terminators were predicted using TransTermHP 2.0 and in the case of more than one terminator within 50 nt of the TES the closest one was used for annotation (Kingsford *et al*., 2007).

### KEGG analysis

KEGG annotations for *B. subtilis* were downloaded from the KEGG website (September 2008) (Kanehisa *et al*., 2008). KEGG annotations were counted for the genes present exclusively expressed in the LB medium, the M9 medium and genes expressed in both (common). From each of these three categories, the five most occurring annotations were selected and the occurrences were plotted as shown in Fig. 1.

### Identification of novel ncRNAs

Segments of five or more probes without known annotation according to the latest GenBank annotation (AL009126.3) and no ORF predicted by EasyGene (Nielsen and Krogh, 2005) were accepted as putative novel ncRNAs if they were expressed above background and neighbouring segments, contained a maximum of 5% potentially cross-hybridizing probes and had higher signal level than same area on the opposite strand. Segments with less than five probes fulfilling the criteria and expressed in both media were also accepted as possible ncRNAs. In addition, all potentially novel ncRNAs were inspected visually. The ncRNAs were named *ncr1*–*ncr84* and are listed in Table S5. We also searched the first 100 nt of each ncRNA for ribosome binding sites with SD (AGGAGG) and 4–10 nt after that a start codon (ATG/CTG/GTG), resulting in five of these coding for small putative CDSs. The DNA sequences corresponding to the 84 segments that passed the above criteria were extracted from the genome sequence (AL009126.3), and were compared by blast to all available Firmicute genome or plasmid sequences [34 species within 42 strains resulting in 225 entries from the CBS Genome Atlas Database version 2.0 (Hallin and Ussery, 2004)] (Altschul *et al*., 1990). For each species the best hit was recorded as per cent identity over the entire ncRNA length. These results are shown in Fig. S7.

### Identification of antisense RNAs

Segments were subjected to the same criteria as for identification of novel ncRNAs, except expression did not have to be higher than on the opposing strand. Additionally the transcripts are antisense to a known gene (GenBank: AL00926.3) with an overlap of more than 10%. The identified antisense transcripts were manually curated leading to 127 transcripts that were named *shd1–127* and are listed in Table S6.

### 3′ UTR identification

Transcripts with a conserved 3′ UTR structure were identified based on multiple alignment of the 220 nucleotides downstream of all genes, performed using clustalx2 (Larkin *et al*., 2007). Structures were made using RNAfold v. 1.6 and the Vienna RNA web suite (Gruber *et al*., 2008).
