# Supplementary material for: The transcriptionally active regions in the genome of Bacillus subtilis
Source: Mol Microbiol. 2009 Aug 26;73(6):1043–57. doi: 10.1111/j.1365-2958.2009.06830.x (PMC2784878; doi:10.1111/j.1365-2958.2009.06830.x)

Figure S1

Histogram of degree of overlap between TARs in the two medias. An overlap of 0 means that the TAR is unique to that media, whereas an overlap of 1 means that the TAR is identical to a tar in the other media.

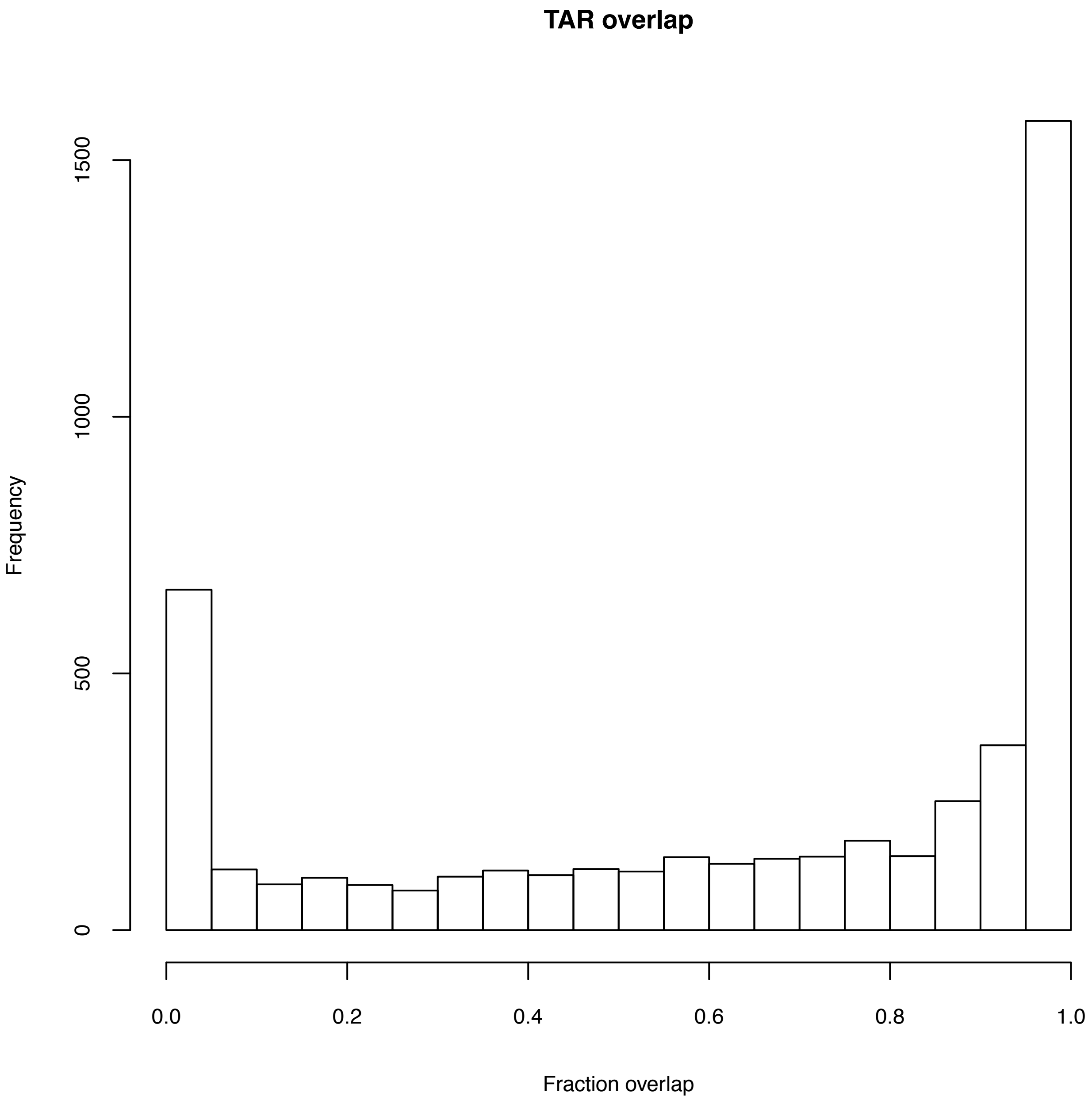

Figure S3

Benchmarking of TARs. (**a**) Shows the ROC-like curve of found genes. The True Positives (TP) are the genes as they are currently annotated and the False Positives (FP) are the same regions but on the opposite strand. (**b**) shows how many of the know Transcription Start Sites (TSSs) that are found as a function of the distance between this and the observed breakpoint. (**c**) Autocorrelation of expression signal as a function of spatial organization of genes (red) or TARs (blue).

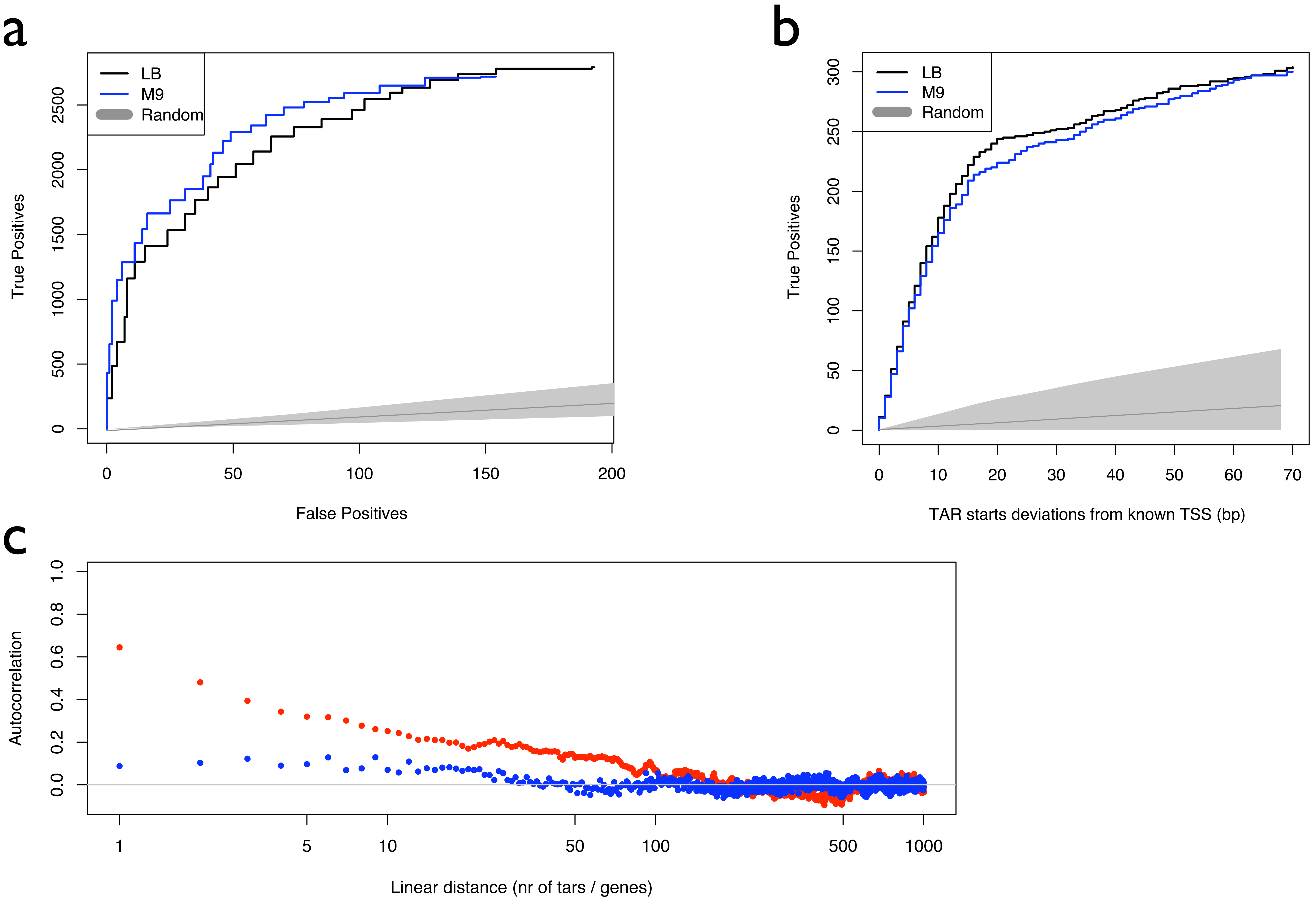

Figure S4

Density of gene expression of sporulation regulons, LB (a) and M9 (b). The regulons are color coded as: *sigF* (green), *sigE* (blue), *sigG* (yellow), *sigK* (red) and all genes (black). The vertical dotted line shows background signal. The composition of each regulon was taken from Steil *et al.*, 2005 and is shown in (c).

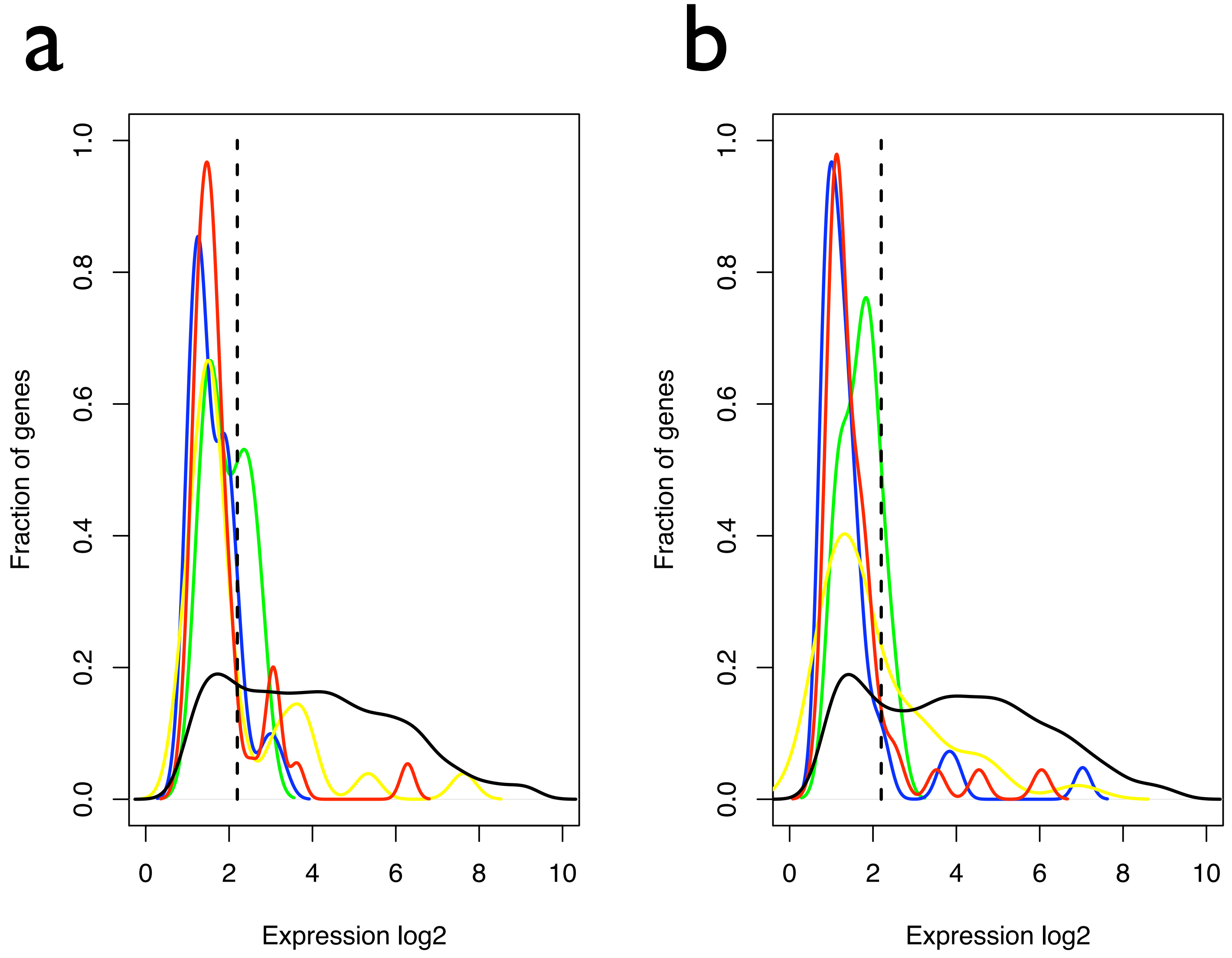

c

Regulon	Genes in regulon
sigF	*bofC, dacF, gpr, lonB, rsfA, spoIIQ, spoIIR, spoIVB, sspN, tlp, yphA, seaA*
sigE	*cotE, cotJA, cotJB, cotJC, cwlD, cwlJ, dacB, mmgA, mmgB, mmgC, mmgD, phoB, safA, spoIID, spoIIIAA, spoIIIAB, spoIIIAC, spoIIIAD, spoIIIAE, spoIIIAF, spoIIIAG, spoIIIAH, spoIIID, spoIVA, spoIVFA, spoIVFB, spoVD, spoVID, ysxE, spoVK, spoVR, usd, yaaH, ydhD, yjmC, exuR, exuT, uxuA, yjmD, uxuB, yknT, spoVM*
sigG	*coxA, csgA, gdh, gerAC, gerBA, gerBB, gerBC, gerD, sigG, sleB, splA, splB, spoIVB, spoVAA, spoVAB, spoVAC, spoVAD, spoVAEA, spoVAEB, spoVT, sspA, sspB, sspC, sspD, sspE, sspF, sspH, sspI, sspJ, sspK, sspL, sspN, tlp, ybaK, yhcN*
sigK	*cgeA, cgeB, cgeC, cgeE, cotA, cotB, cotD, cotF, cotG, cotH, cotS, cotV, cotW, cotX, cotY, cotZ, gerE, gerPA, gerPB, gerPC, gerPE, gerPF, spoIIIC, spoVFA, spoVFB, spsA, spsC, spsD, spsE, spsF, spsG, spsI, spsJ, spsK, sspG, tgl, yabG, ydgB, cotP, ydgA, ykvP, cotR, yvdP*

Figure S5

Gene expression of prophage and prophage-like elements. Upper row show phage element expression in LB media and lower row gene expression in M9 media. Squares represents genes in the phage elements. The color scale range from white (low expression) to blue (high expression). Arrows indicate the sublancin area (*bdbB* to *sunI*) in the *SPβ* prophage.

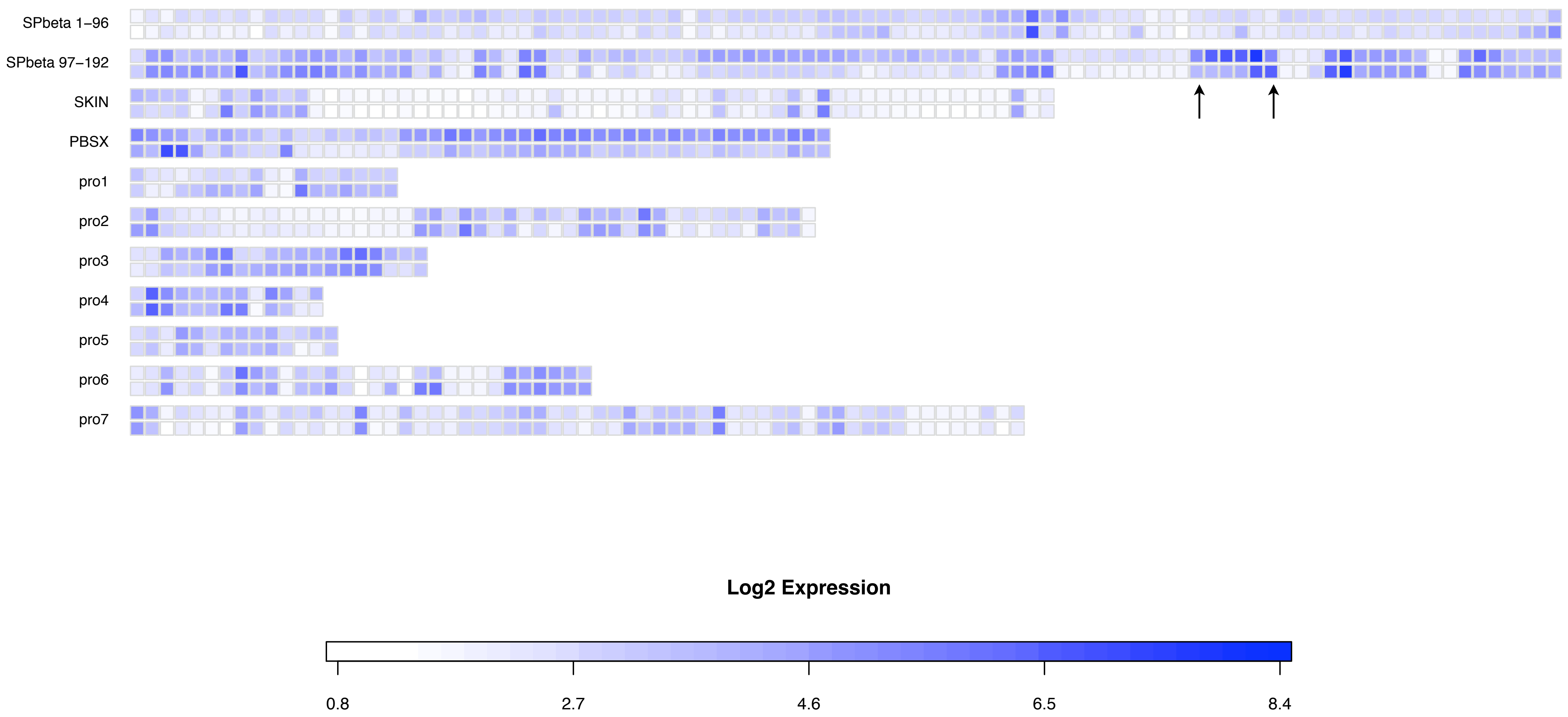

SPβ:2152-2286 kb, *PBSX*: 1316-1347 kb, *SKIN*: 2653-2700 kb, *pro1*: 202-220 kb, *pro2*:529-570 kb, *pro3*: 652-665 kb, *pro4*:1262-1270 kb, *pro5*:1879-1891 kb, *pro6*: 2046-2073 kb, *pro7*: 2707-2756 kb.

Figure S6

Density plots of gene expression of prophage and prophage-like elements. Expression for LB and M9 is shown blue and red, respectively. Prophage coordinates are shown in Fig. S5.

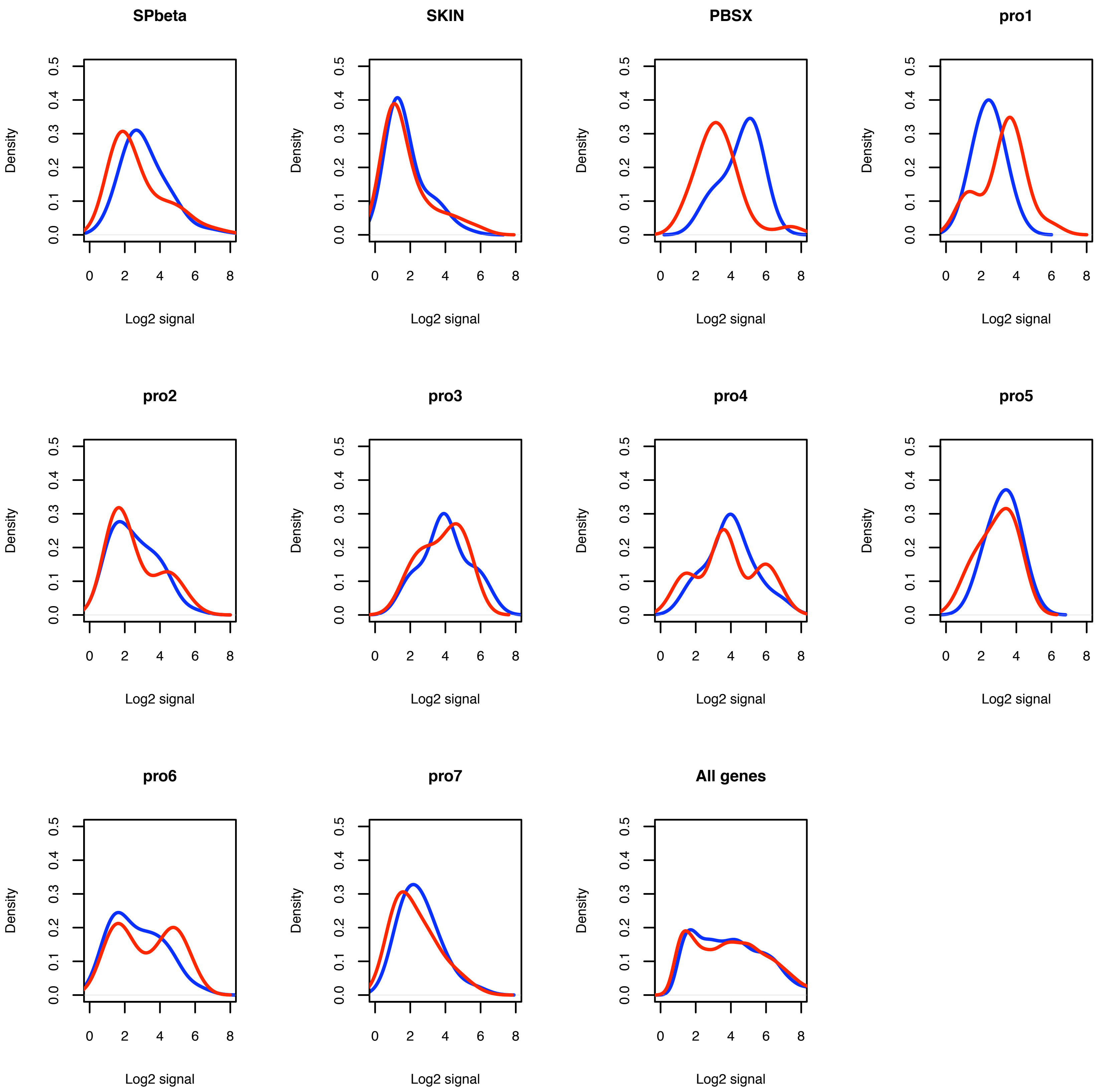

Figure S7

Conservation plot of non coding RNAs (*ncr*) identified. Nucleotide sequence of identified *ncr*s was compared against all available Firmicute sequences (genome and plasmid) and the maximum hit is plotted. The color scale ranges from black (0% identity) to white (100% identity).

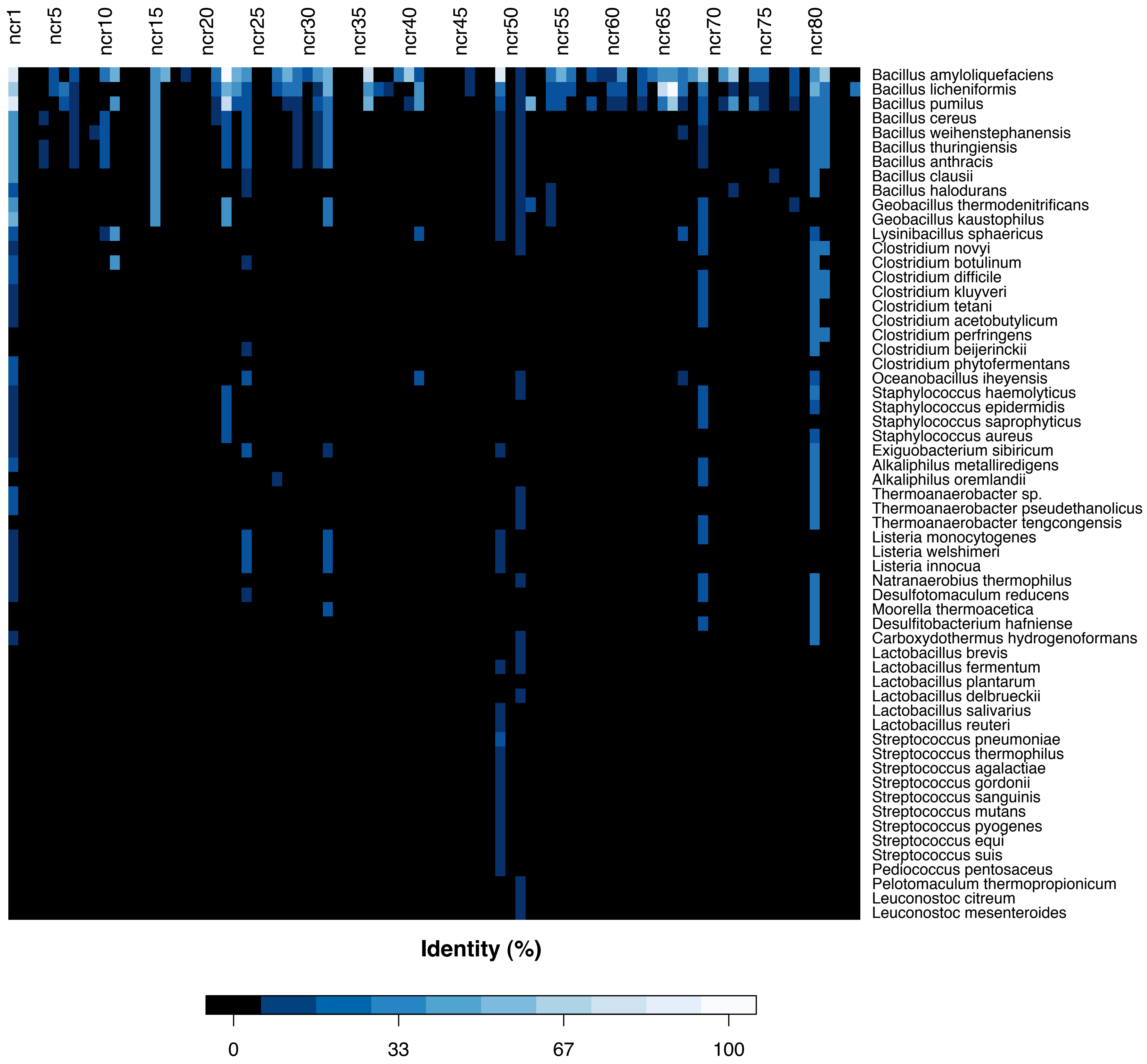

Figure S8

Sense overlap and antisense/ratio of antisense transcripts. (a) Fraction of sense genes (cds) overlapped by antisense transcript. Two distributions (< 0.7 and > 0.7) of overlaps can be seen. (b) For the 127 antisense transcripts, log2 sense (LB/M9) and log2 antisense (LB/M9) ratio is plotted. If antisense transcript is the primary regulator of the sense area at the conditions tested, the antisense and sense ratios would expect to be anti-correlated (upper left and lower right quarters). The number plotted corresponds to the shd nomenclature (e.g. 4 = *shd4*).

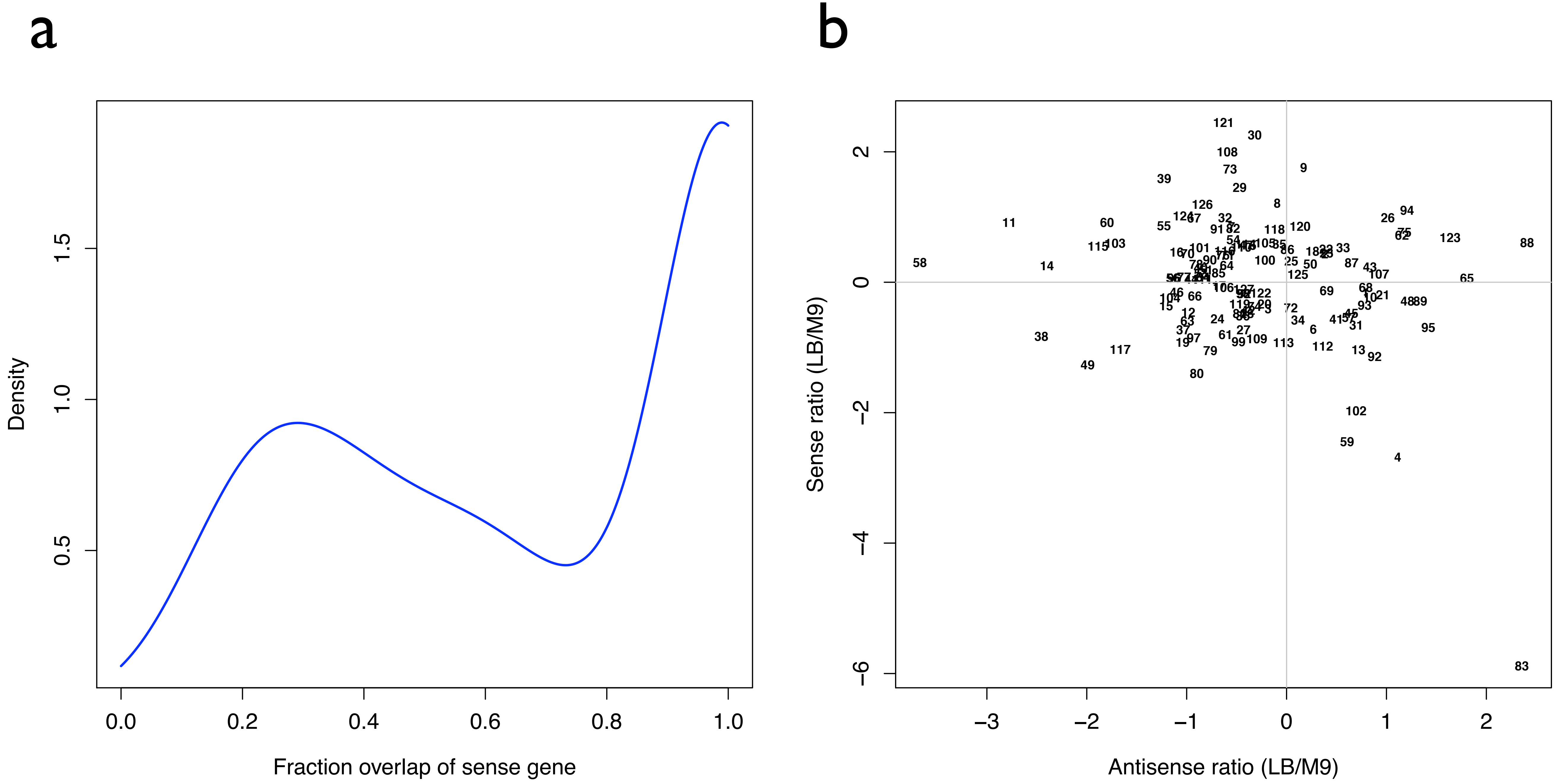

Figure S9

Folding of conserved 3'UTR RNA elements. All elements were folded using RNAfold v1.6 and bases are coloured according to base-pair probabilities, from 0 to 1 (purple, blue, green, yellow to red).

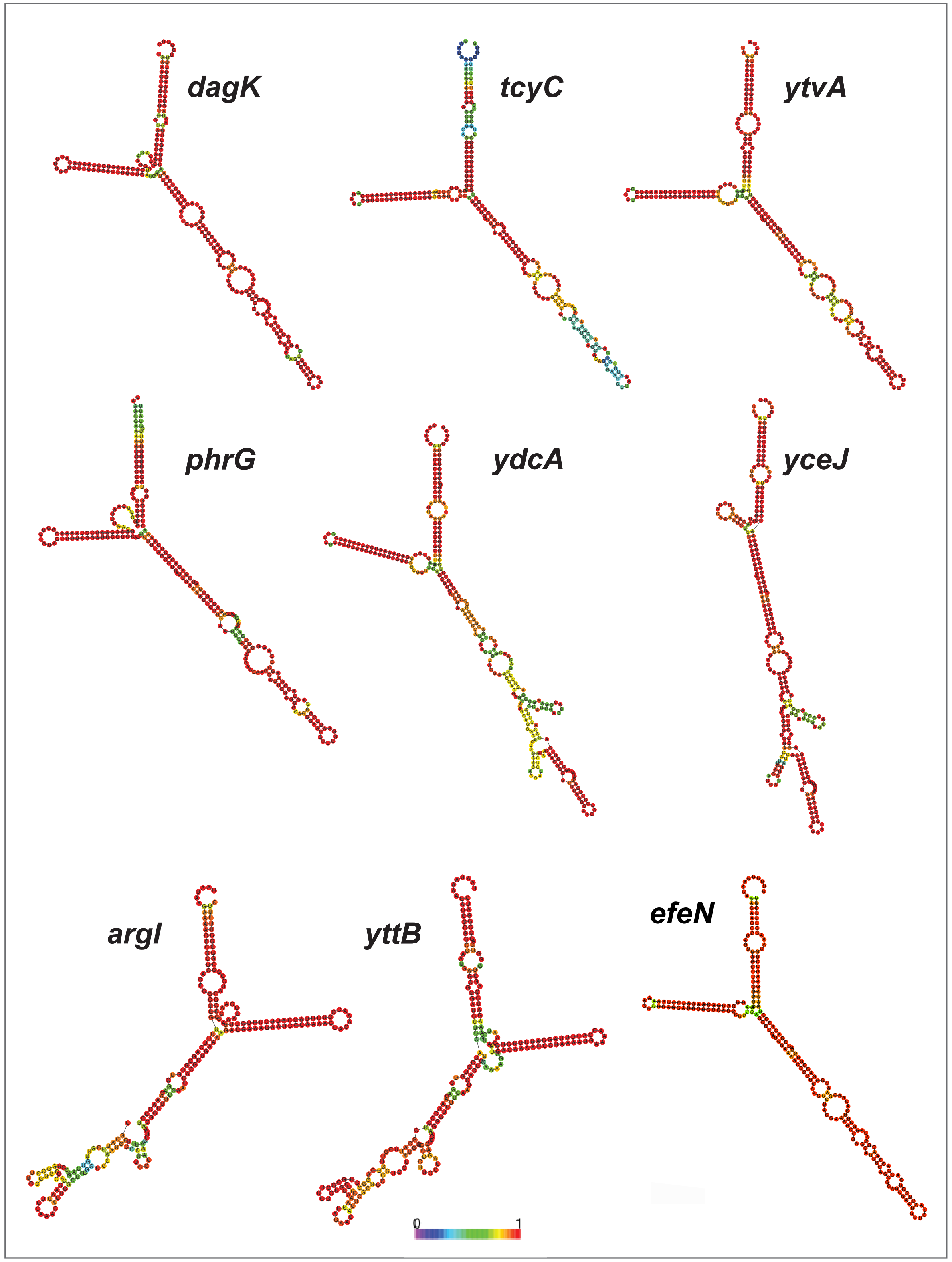

Supplement: Supplementary file 7 [file mmi0073-1043-SD7.pdf]
