# Supplementary material for: The transcriptionally active regions in the genome of Bacillus subtilis
Source: Mol Microbiol. 2009 Aug 26;73(6):1043–57. doi: 10.1111/j.1365-2958.2009.06830.x (PMC2784878; doi:10.1111/j.1365-2958.2009.06830.x)
Supplement: Supplementary file 8 [file mmi0073-1043-SD8.pdf]

Figure S2

The following pages show the level of expression along the genome of Bacillus subtilis when growing on rich (LB) and minimal (M9) medium. The signal from genomic DNA hybridized to the same tiling chip (Rasmussen *et al.*, 2009) is also shown. Each page contain two similar plots each presenting ~44 Kb of the genome. The two blue bands show the expression in LB and M9 respectively on the positive strand, the two magenta bands show the minus strand. The darker the color the higher the expression in the given position. On top of the bands the expression level is plotted as a scaled version (0-10) of the log2 to the fold change (signal/background signal). In the middle of each plot the gDNA signal is shown for both strands, here the color scale is yellow-green-black, where black is low and yellow is high. Along the genome, in all 6 bands in the same positions, white regions indicate regions were the recent re-sequencing of the genome (AL009126.3) have shown either inserts or regions with very low similarity to the last version (see Rasmussen *et al.*, 2009). Annotated genes are shown as black arrows in the top and the bottom of each plot (annotation from AL009126.3), indicating genes on the positive and negative strand respectively. New genes are shown as red arrows below (+ strand) and above (- strand) each band. Both predicted and experimentally verified terminators and sigma-factor binding sites are shown on top of the gDNA bands. Dark red triangles: Experimentally verified terminators, red triangles: Predicted terminators, stars: Experimentally verified sigma-factor binding sites, green dots: Predicted sigma-factor binding sites.

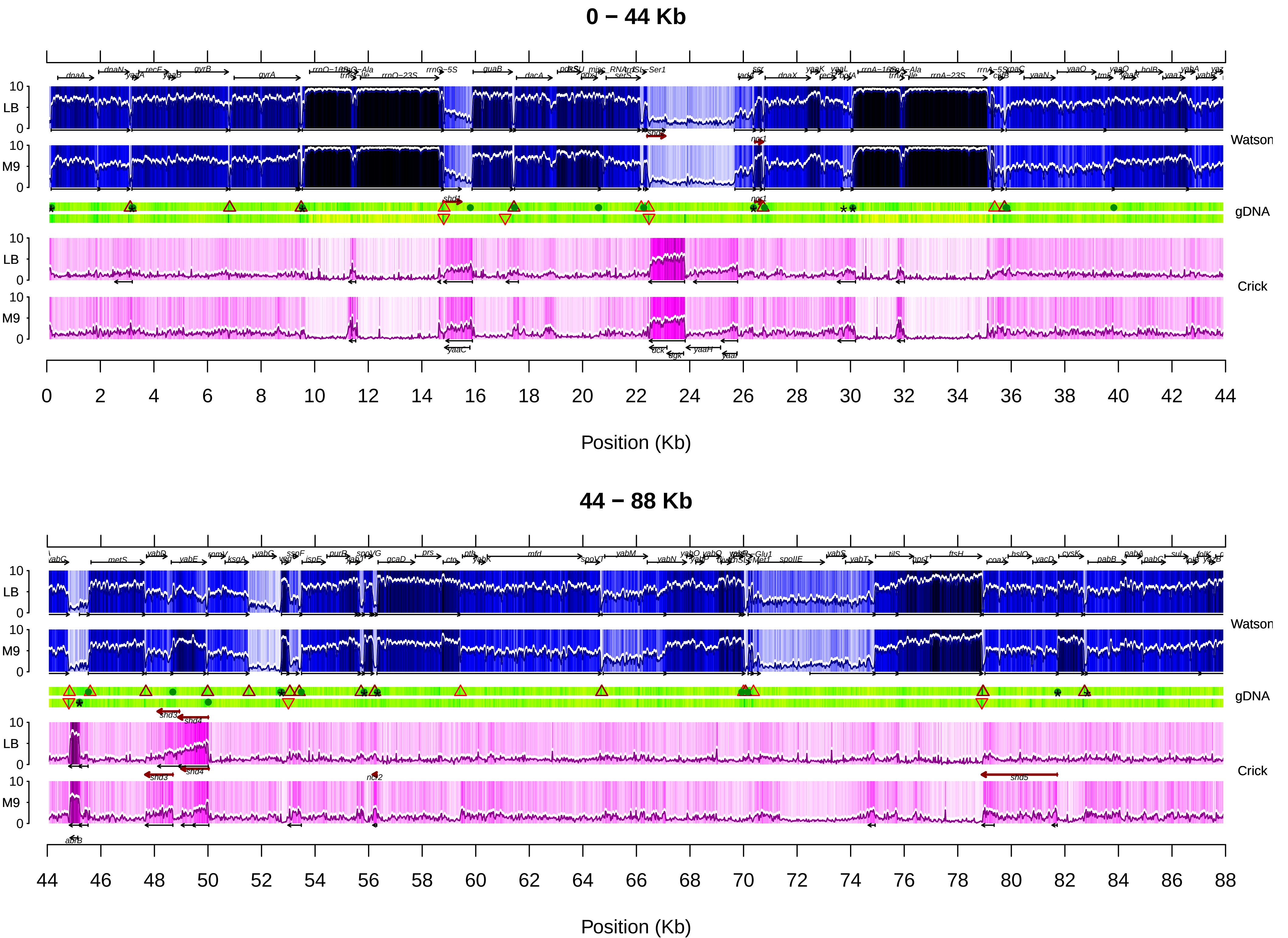
0 − 44 Kb
LB
M9
Watson
gDNA
Crick
Position (Kb)
44 − 88 Kb
Position (Kb)

88 − 132 Kb

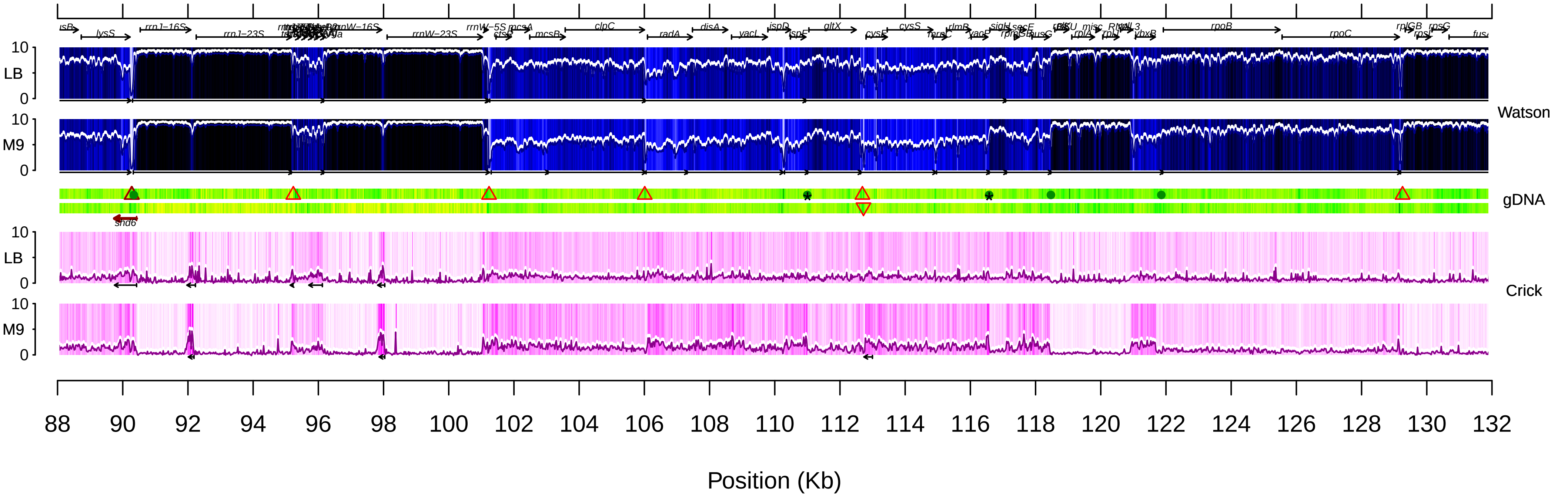

132 − 176 Kb

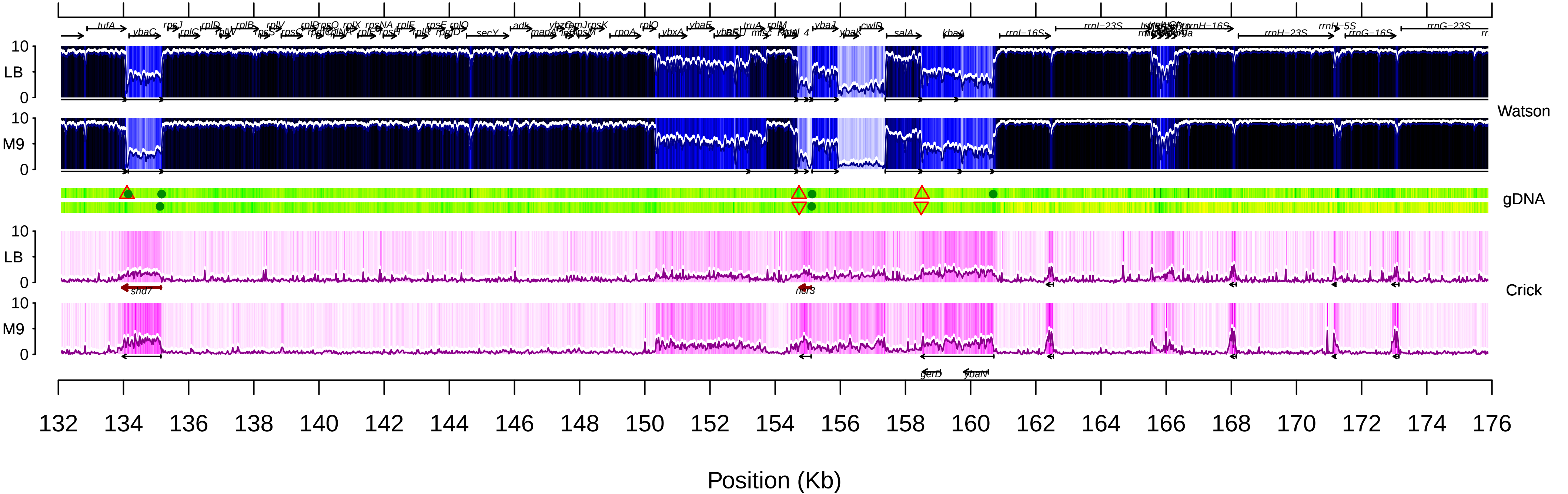

176 − 220 Kb

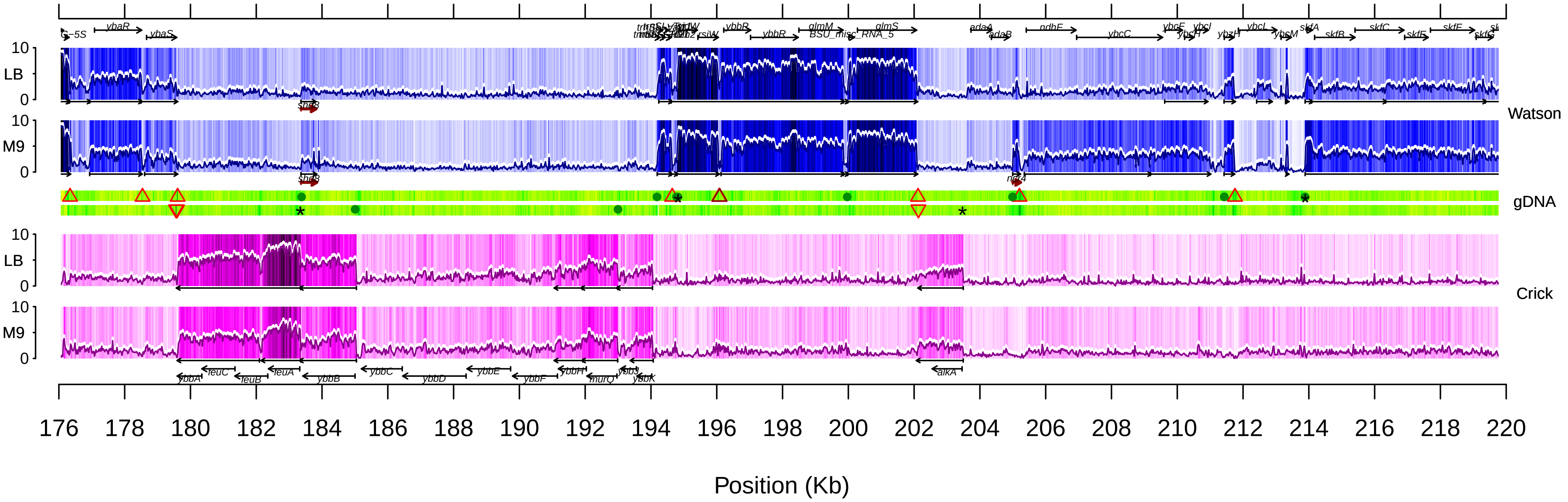

220 − 264 Kb

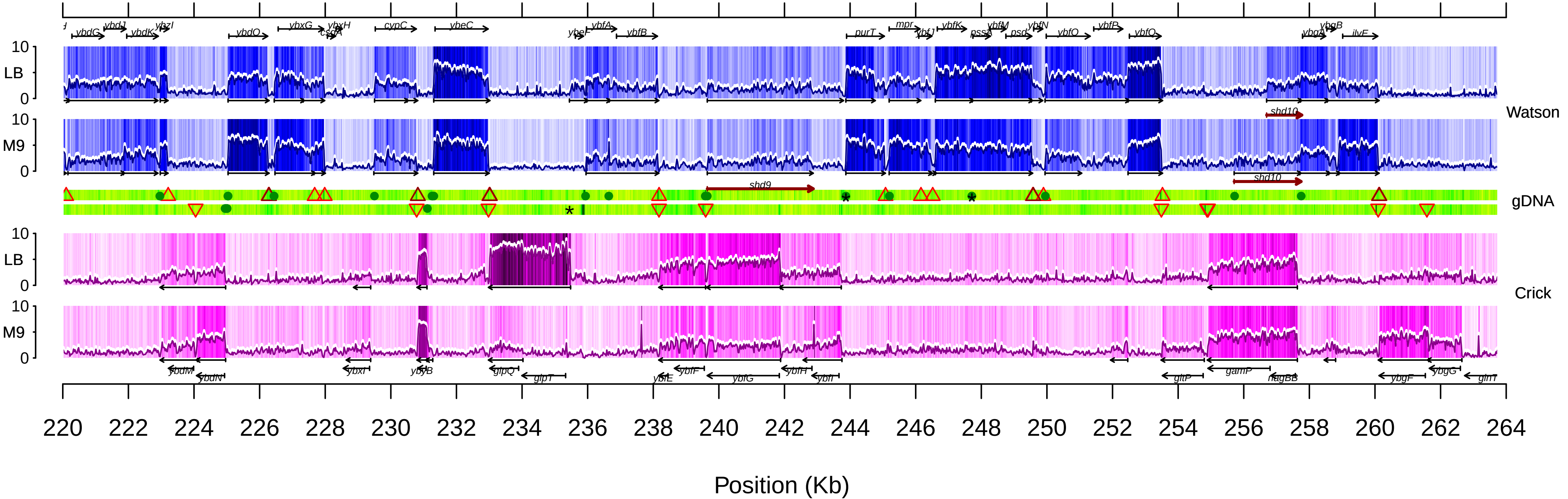

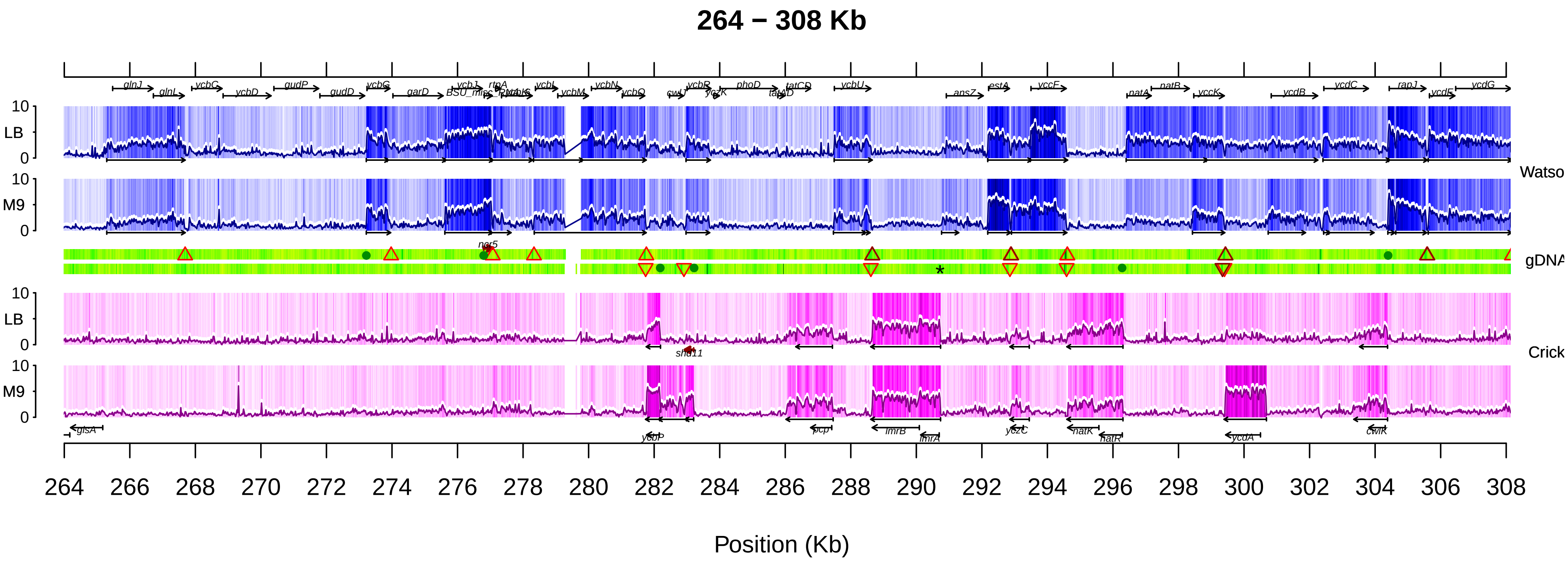
264 − 308 Kb
LB
M9
Watso
gDNA
Crick
264 266 268 270 272 274 276 278 280 282 284 286 288 290 292 294 296 298 300 302 304 306 308
Position (Kb)

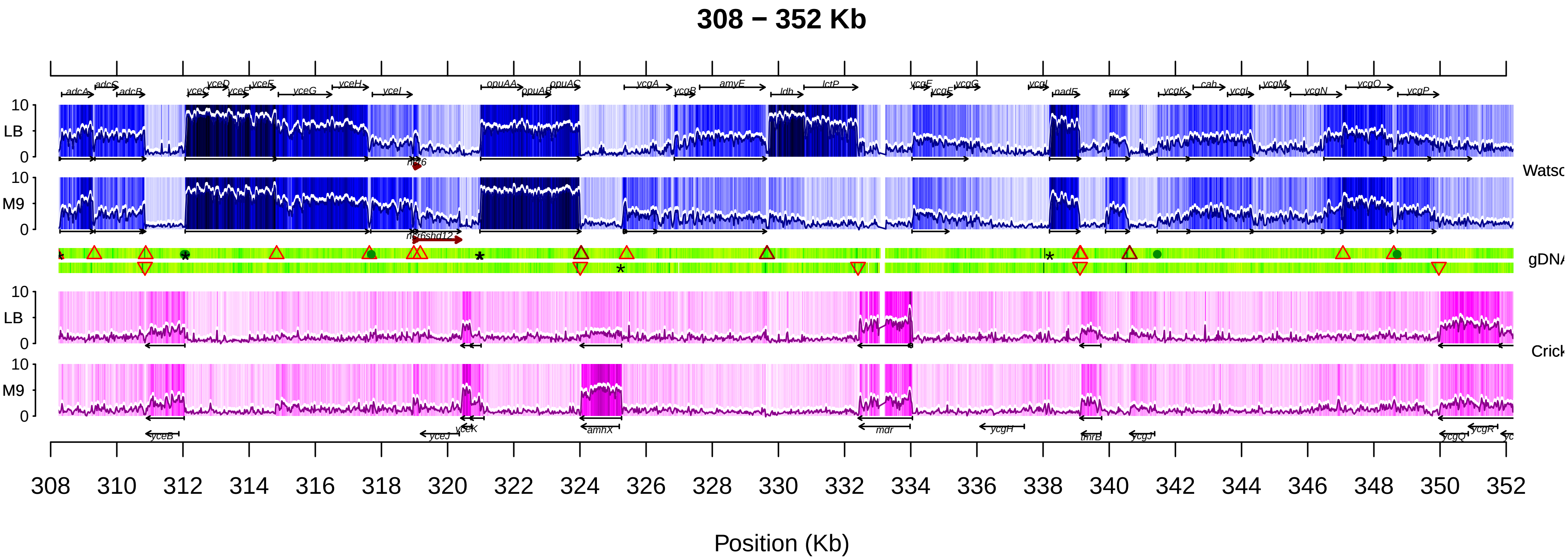
308 − 352 Kb
LB
M9
Watsc
gDNA
Crick
308 310 312 314 316 318 320 322 324 326 328 330 332 334 336 338 340 342 344 346 348 350 352
Position (Kb)

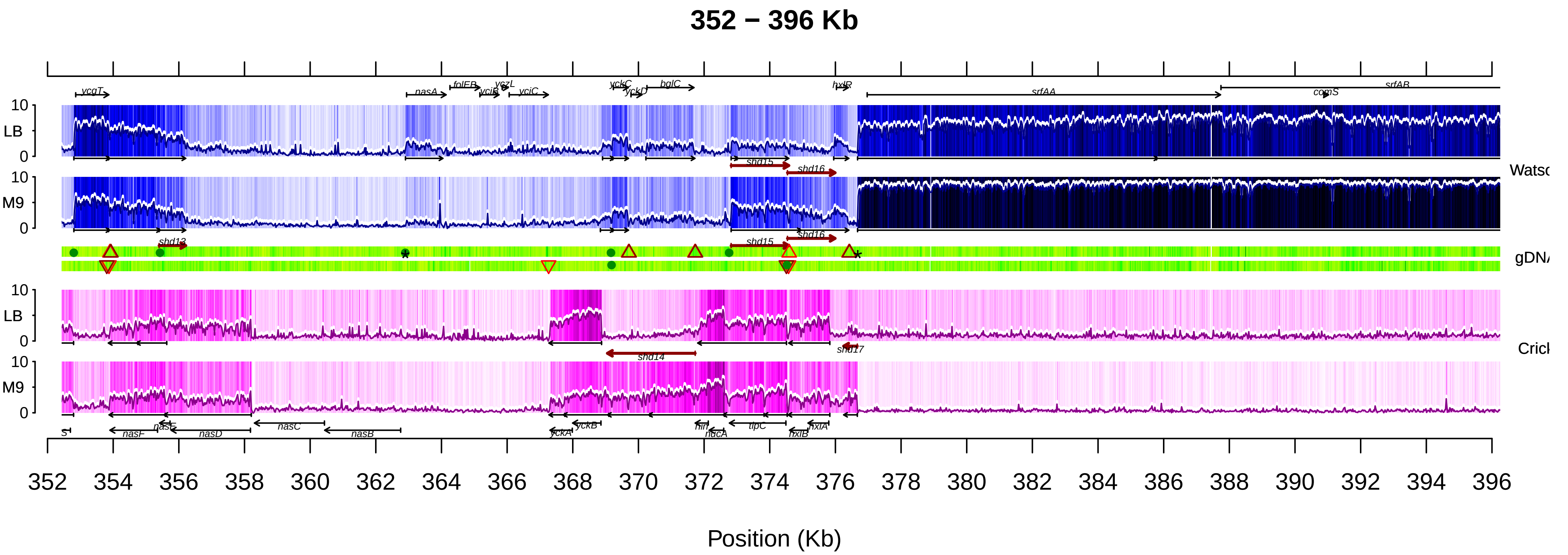
352 − 396 Kb
LB
M9
Watsc
gDNA
Crick
shd13
shd14
shd15
shd16
shd17
352
354
356
358
360
362
364
366
368
370
372
374
376
378
380
382
384
386
388
390
392
394
396
Position (Kb)

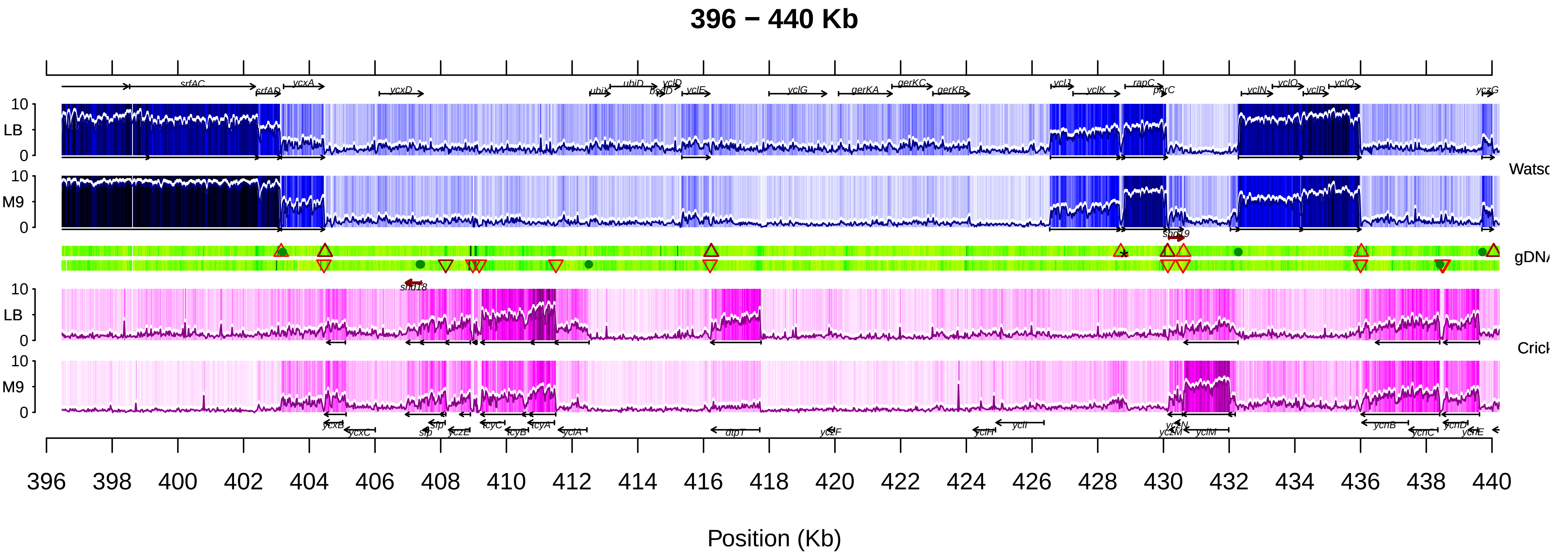
396 − 440 Kb
LB
M9
Watsc
gDNA
Crick
shd18
shd19
396
398
400
402
404
406
408
410
412
414
416
418
420
422
424
426
428
430
432
434
436
438
440
Position (Kb)

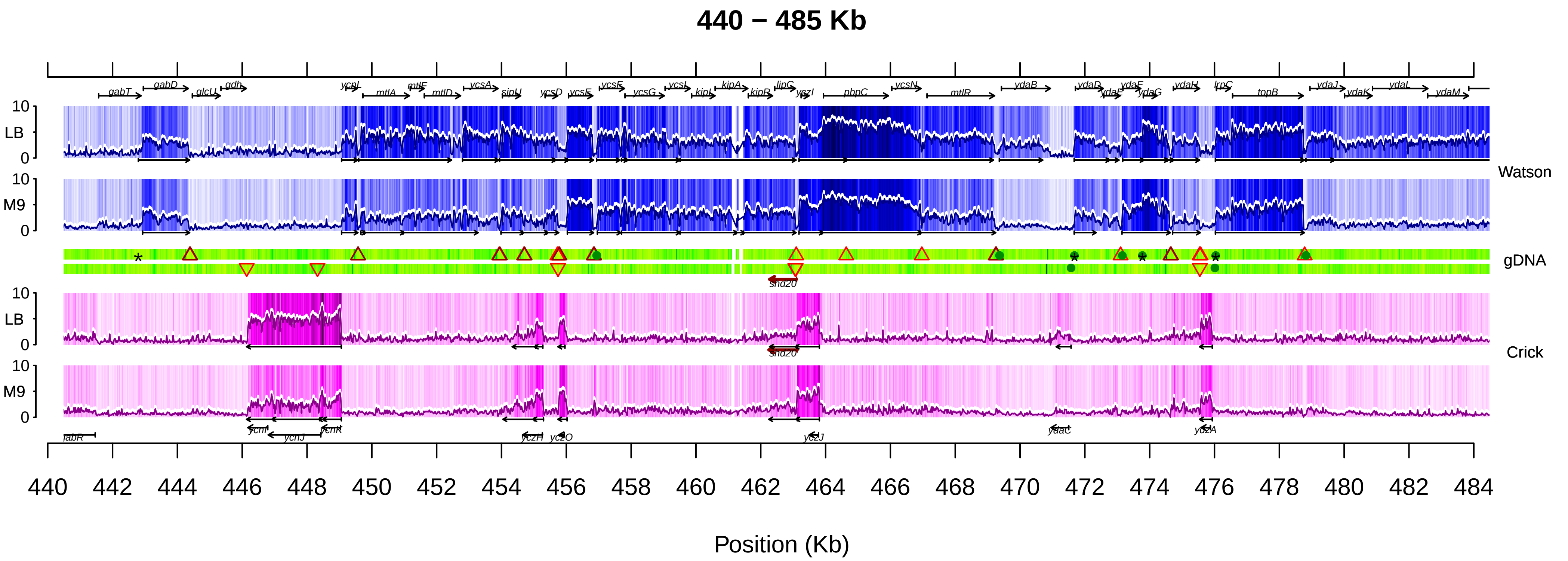
440 − 485 Kb
LB
M9
Watson
gDNA
Crick
440 442 444 446 448 450 452 454 456 458 460 462 464 466 468 470 472 474 476 478 480 482 484
Position (Kb)

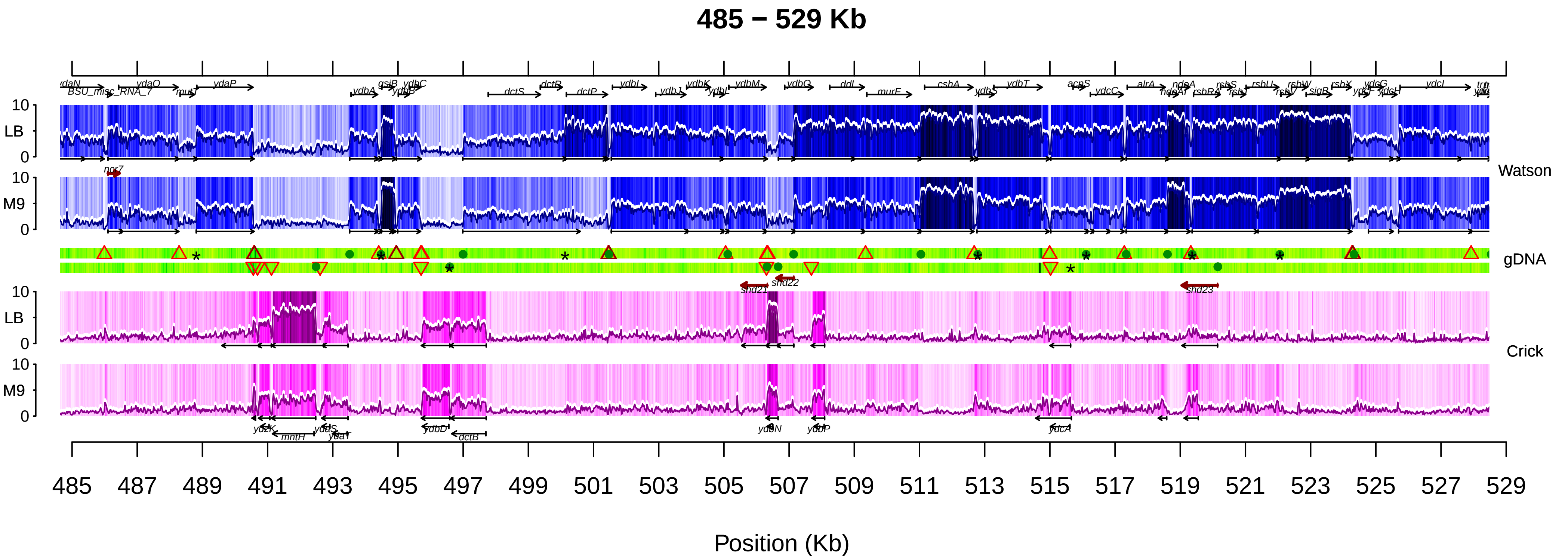
485 − 529 Kb
LB
M9
Watson
gDNA
Crick
485 487 489 491 493 495 497 499 501 503 505 507 509 511 513 515 517 519 521 523 525 527 529
Position (Kb)

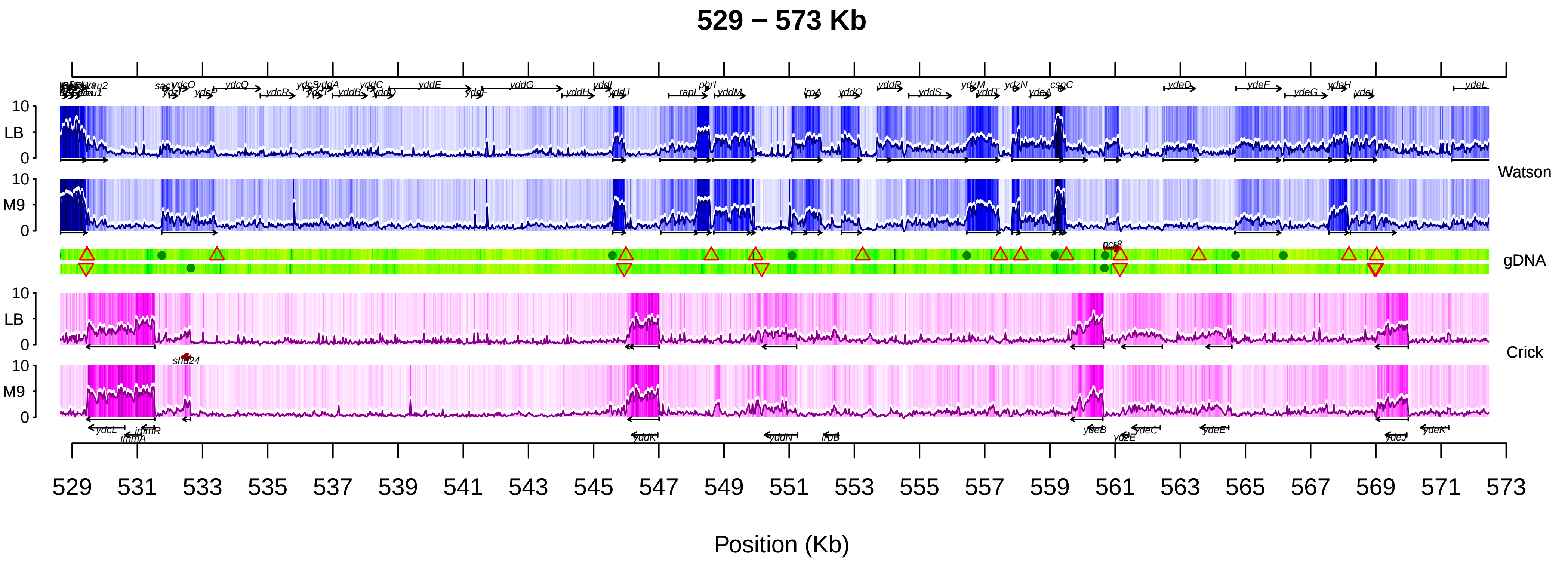

529 − 573 Kb
LB
M9
Watson
gDNA
Crick
10
0
529 531 533 535 537 539 541 543 545 547 549 551 553 555 557 559 561 563 565 567 569 571 573
Position (Kb)

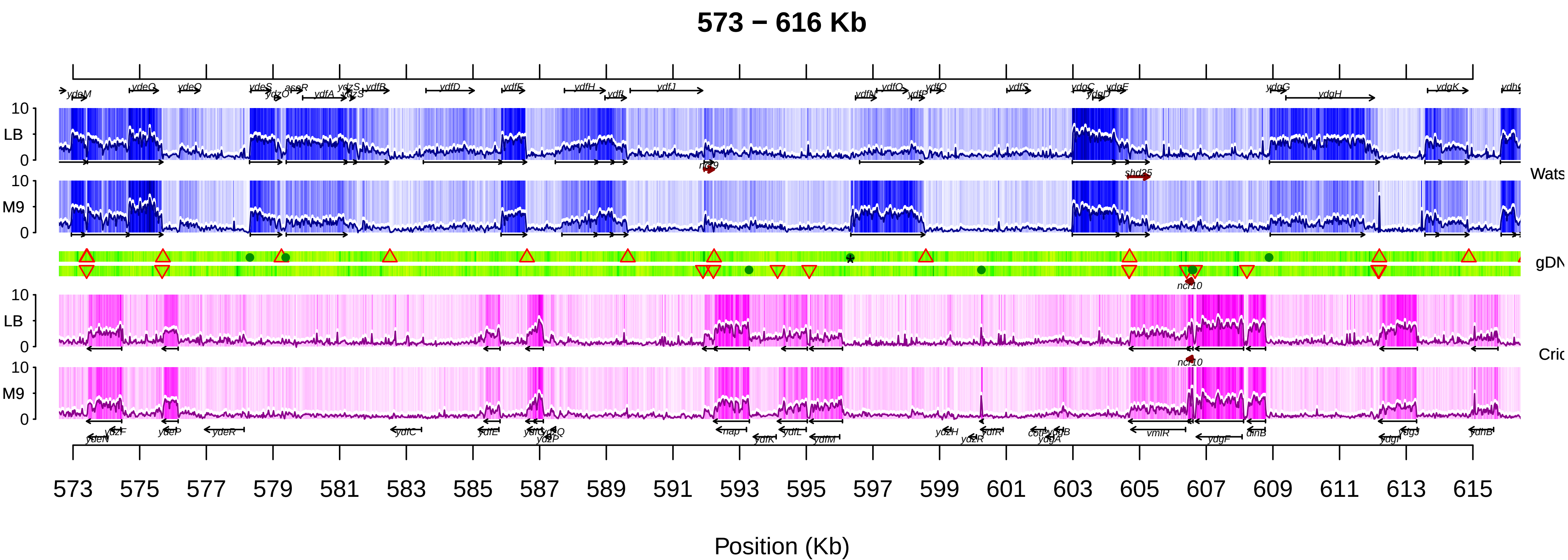

573 − 616 Kb
LB
M9
10
0
573 575 577 579 581 583 585 587 589 591 593 595 597 599 601 603 605 607 609 611 613 615
Position (Kb)

616 − 660 Kb

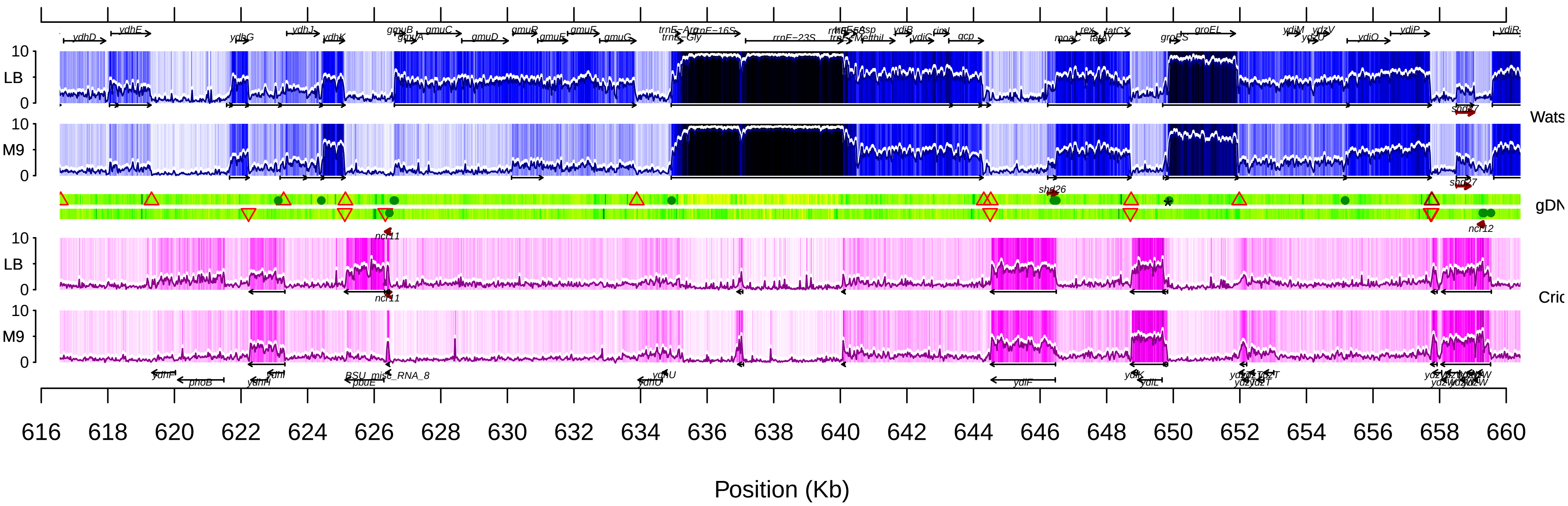

660 − 705 Kb

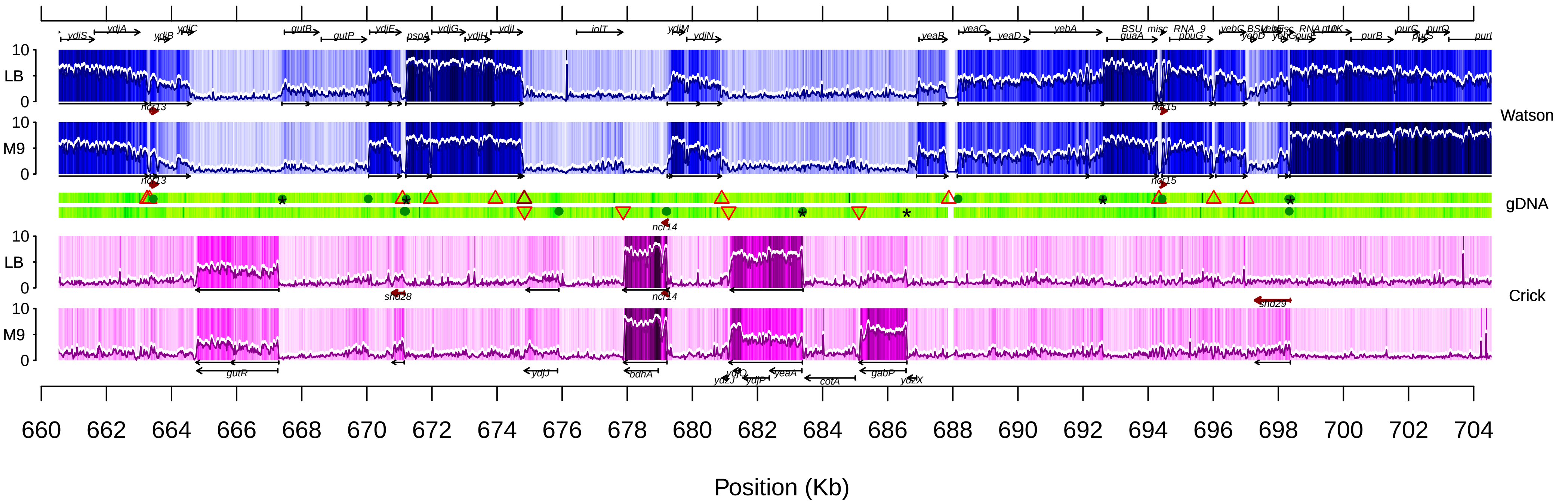

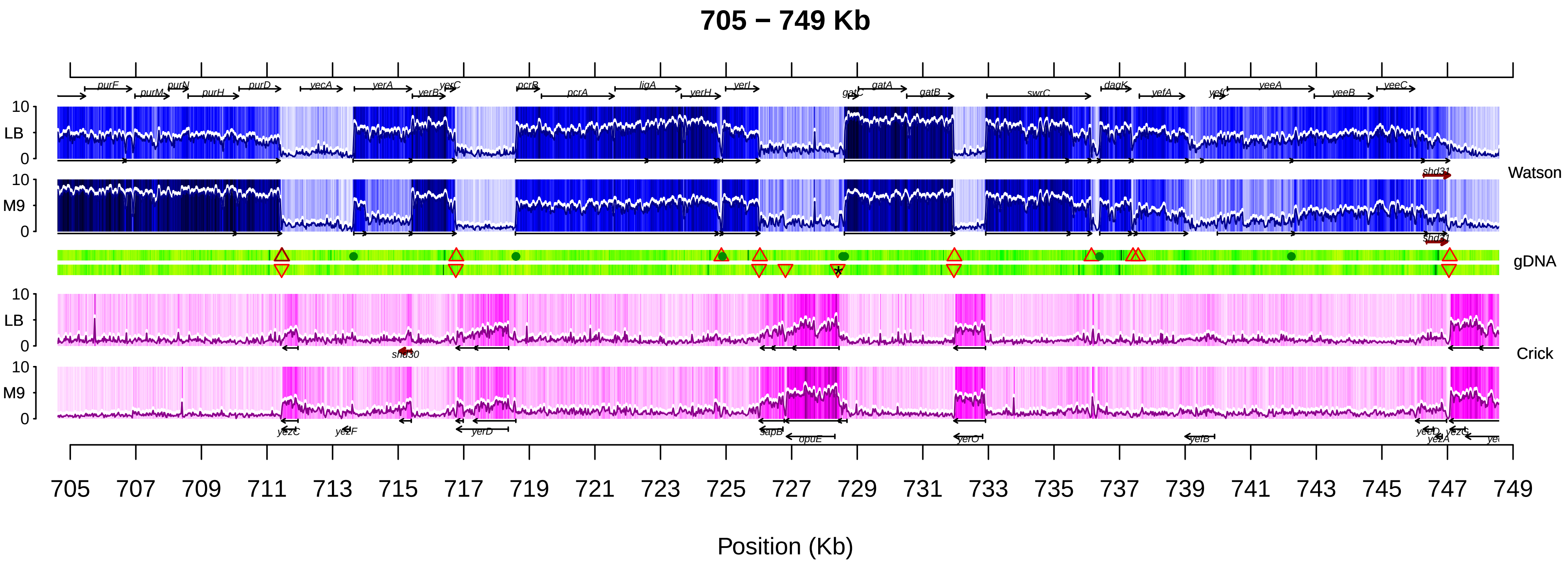

705 − 749 Kb
LB
M9
Watson
gDNA
Crick
705 707 709 711 713 715 717 719 721 723 725 727 729 731 733 735 737 739 741 743 745 747 749
Position (Kb)

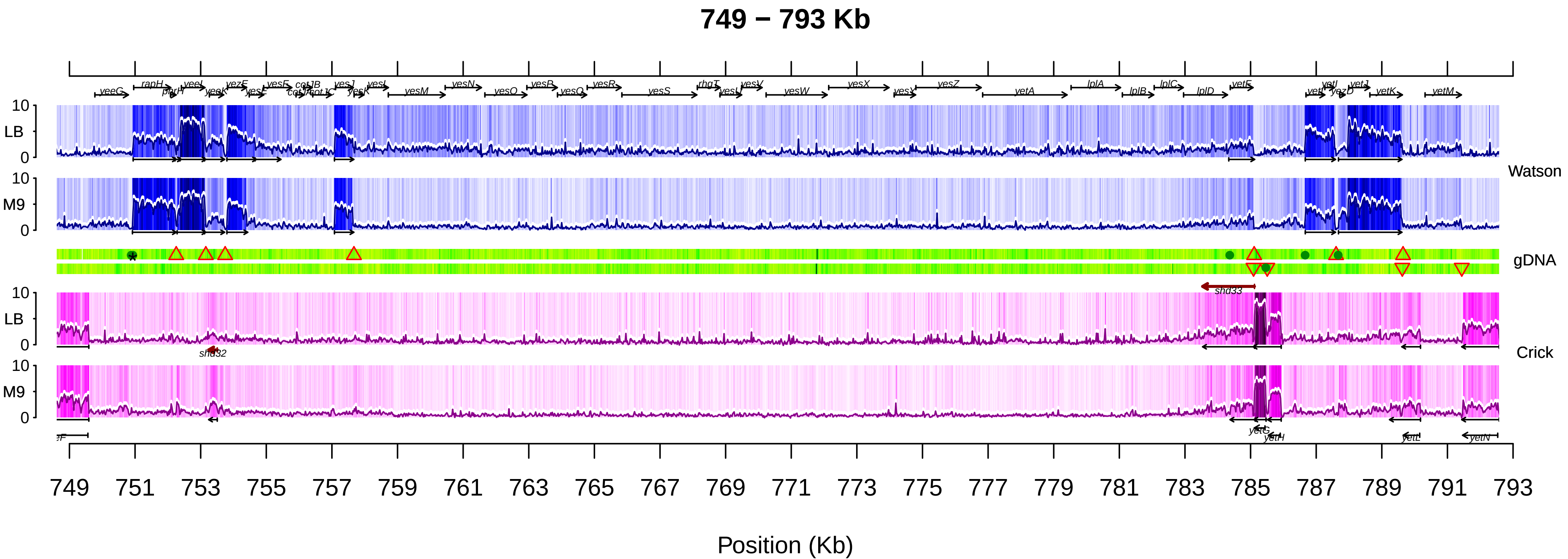

749 − 793 Kb
LB
M9
Watson
gDNA
Crick
749 751 753 755 757 759 761 763 765 767 769 771 773 775 777 779 781 783 785 787 789 791 793
Position (Kb)

793 − 837 Kb

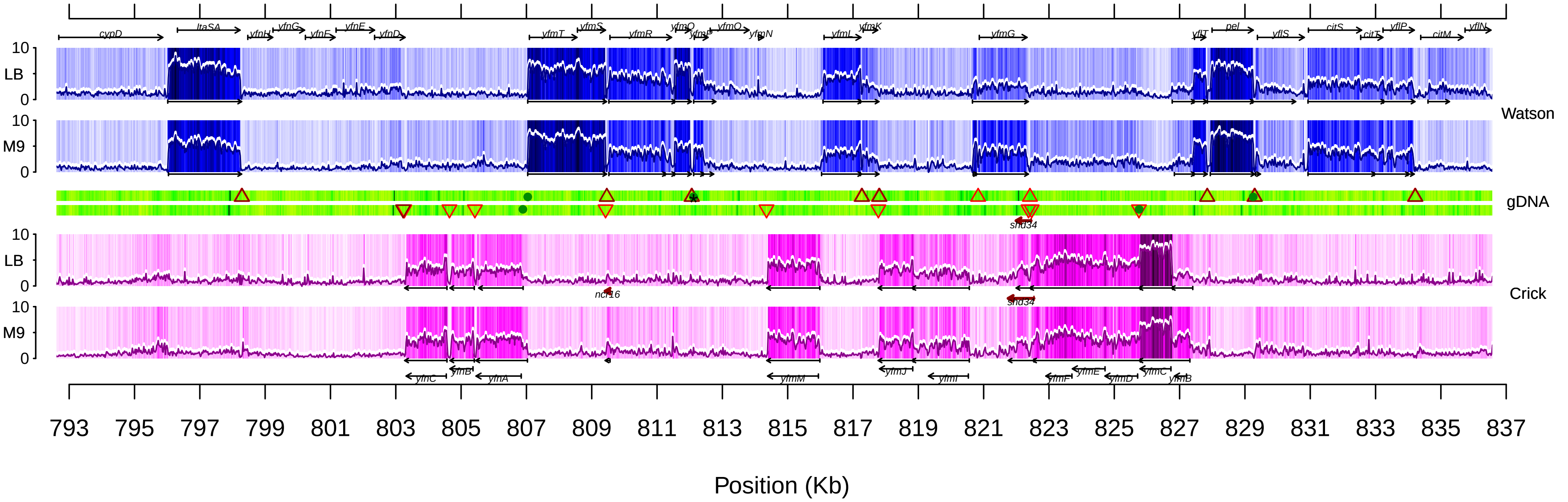

837 − 881 Kb

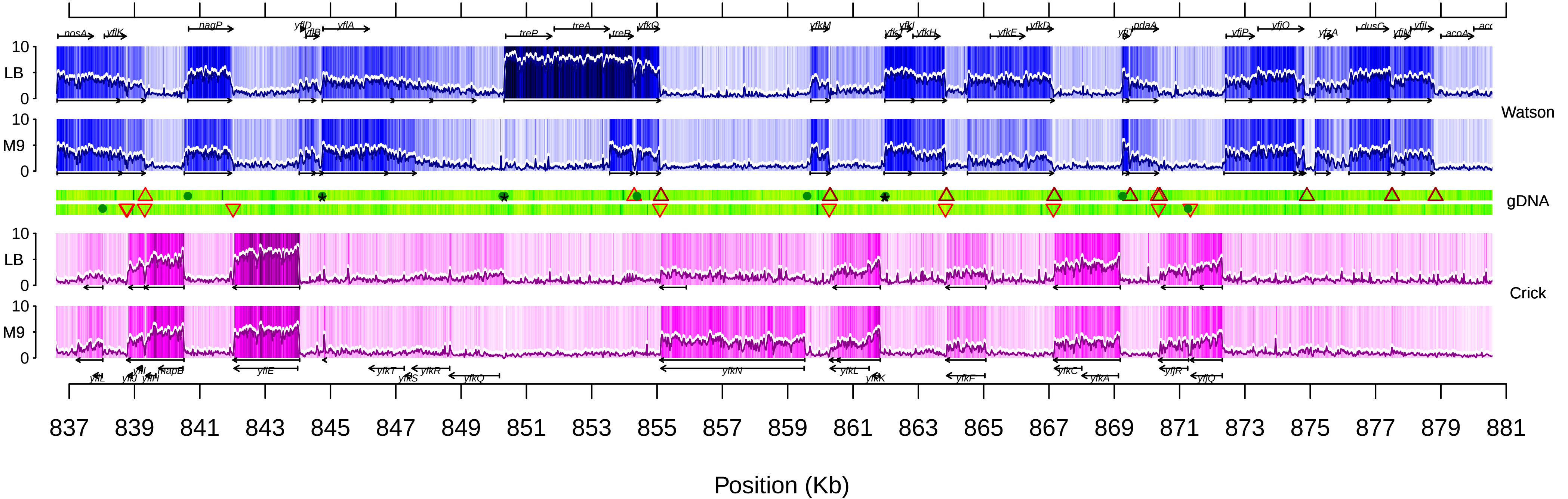

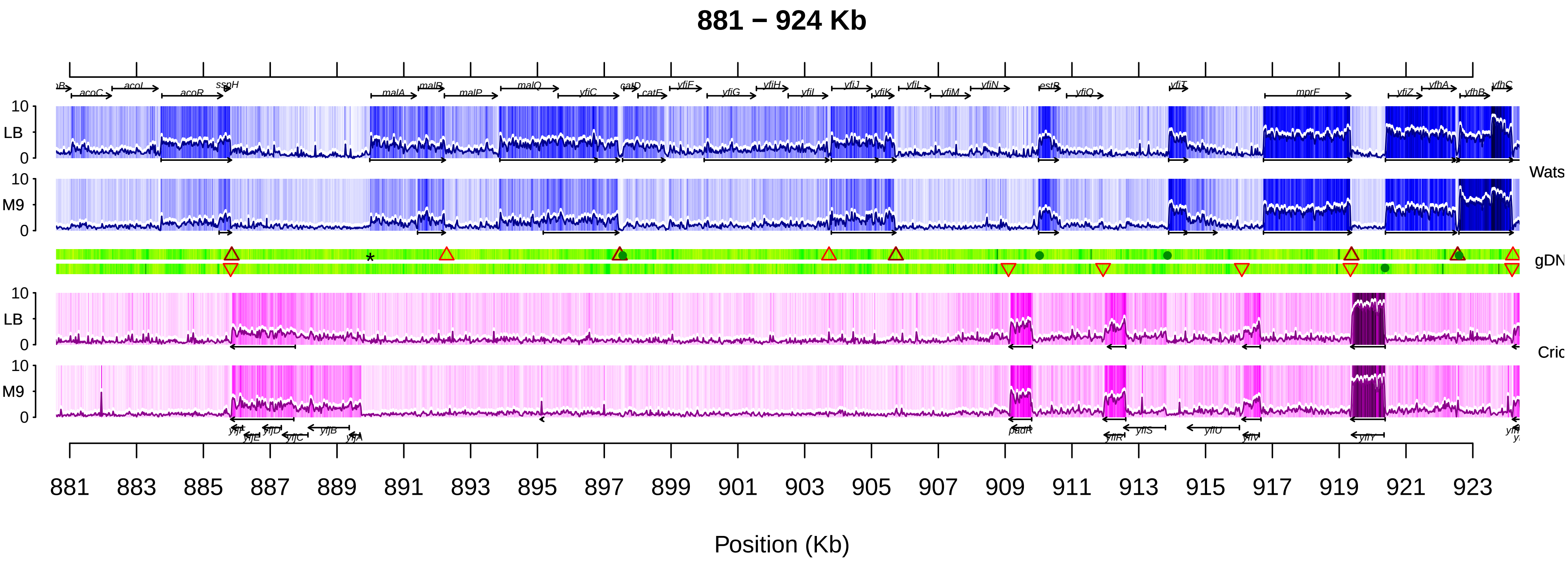
881 − 924 Kb
10
LB
0
10
M9
0
Wats
gDN
10
LB
0
10
M9
0
Cric
881 883 885 887 889 891 893 895 897 899 901 903 905 907 909 911 913 915 917 919 921 923
Position (Kb)

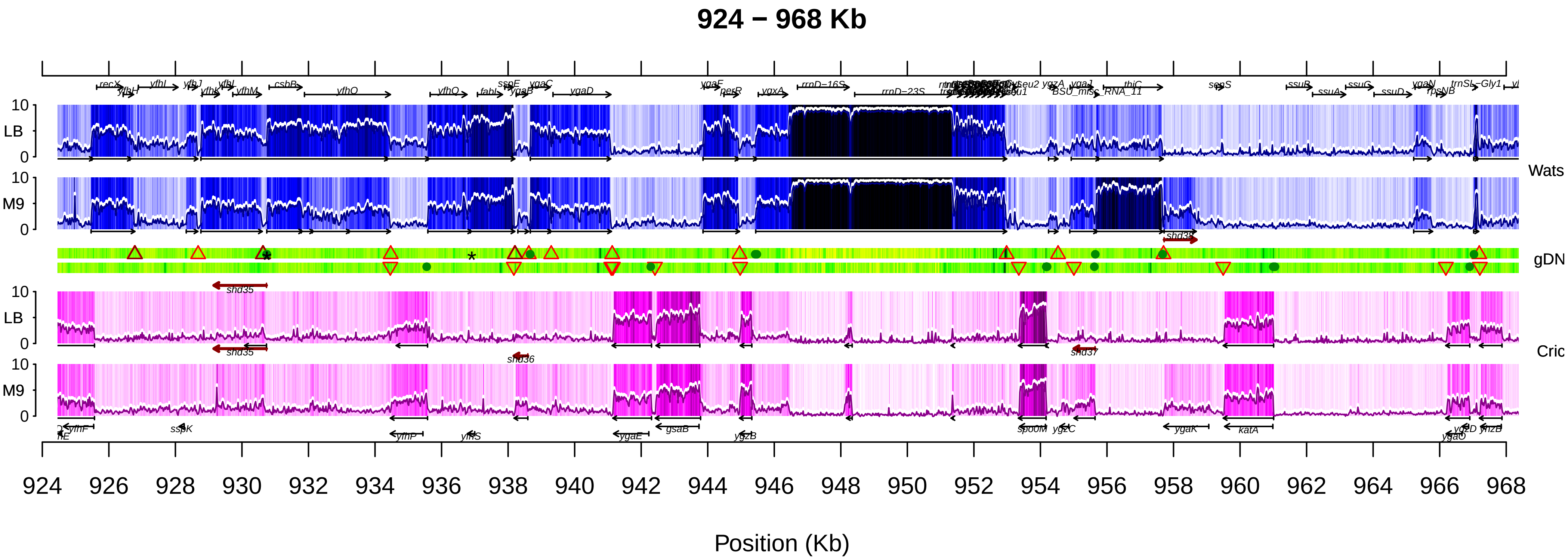
924 − 968 Kb
10
LB
0
10
M9
0
Wats
gDN
shd35
shd36
shd37
shd38
10
LB
0
10
M9
0
Cric
924 926 928 930 932 934 936 938 940 942 944 946 948 950 952 954 956 958 960 962 964 966 968
Position (Kb)

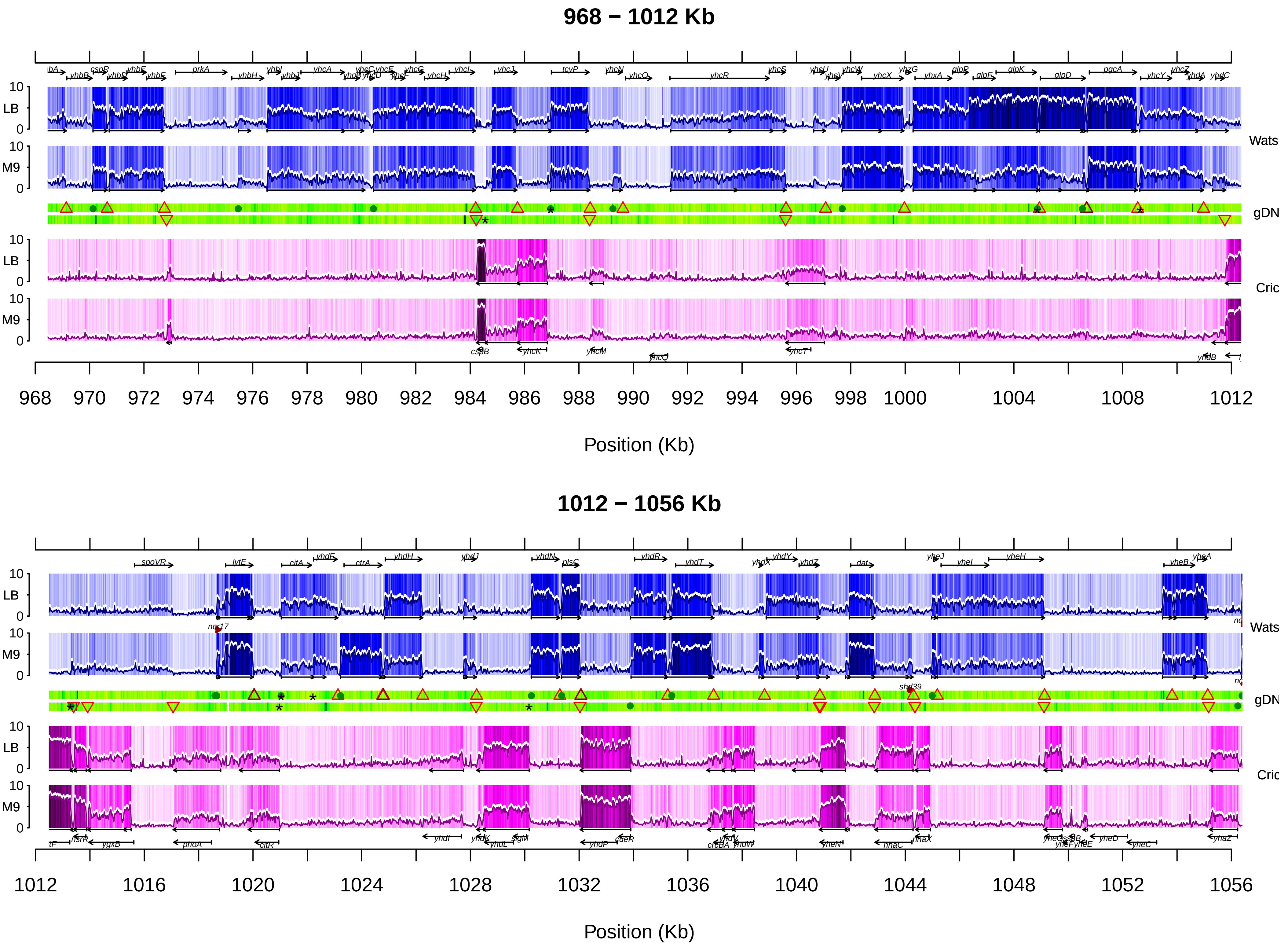
968 − 1012 Kb
LB
M9
Wats
gDN
Cric
968 970 972 974 976 978 980 982 984 986 988 990 992 994 996 998 1000 1004 1008 1012
Position (Kb)
1012 − 1056 Kb
LB
M9
Wats
gDN
Cric
1012 1016 1020 1024 1028 1032 1036 1040 1044 1048 1052 1056
Position (Kb)

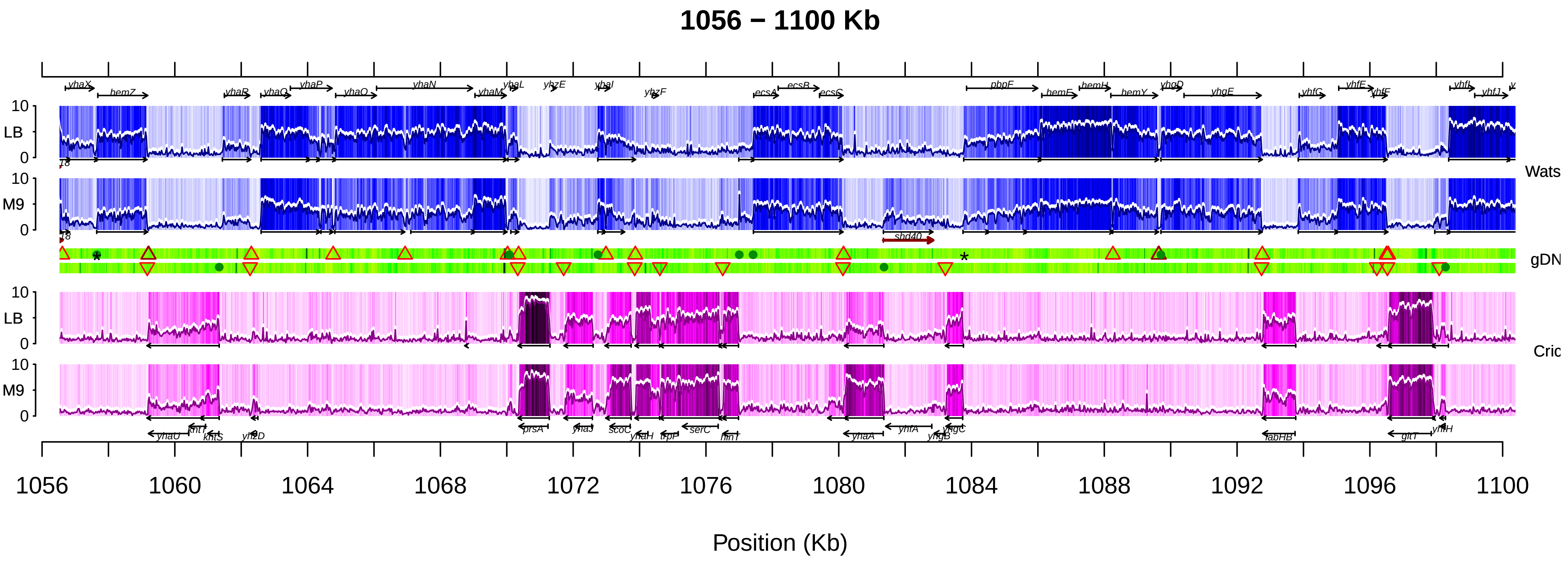
1056 − 1100 Kb
LB
M9
Wats
gDN
Cric
1056
1060
1064
1068
1072
1076
1080
1084
1088
1092
1096
1100
Position (Kb)

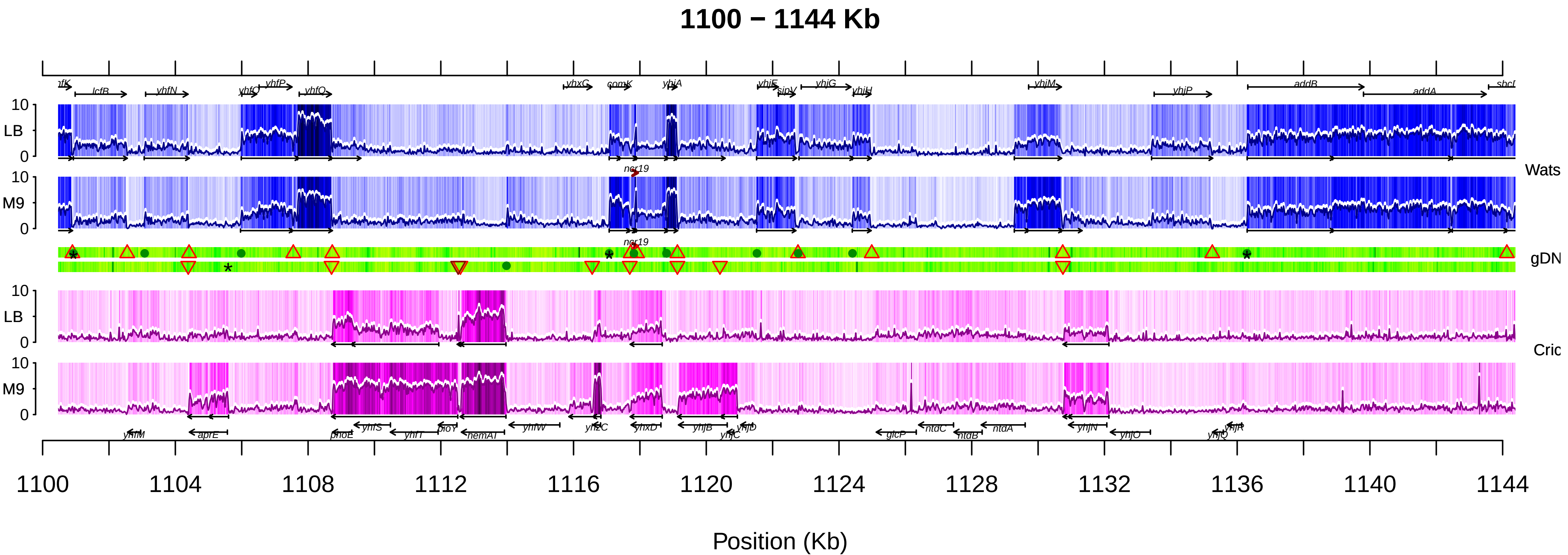
1100 − 1144 Kb
LB
M9
Wats
gDN
Cric
1100
1104
1108
1112
1116
1120
1124
1128
1132
1136
1140
1144
Position (Kb)

1144 − 1188 Kb

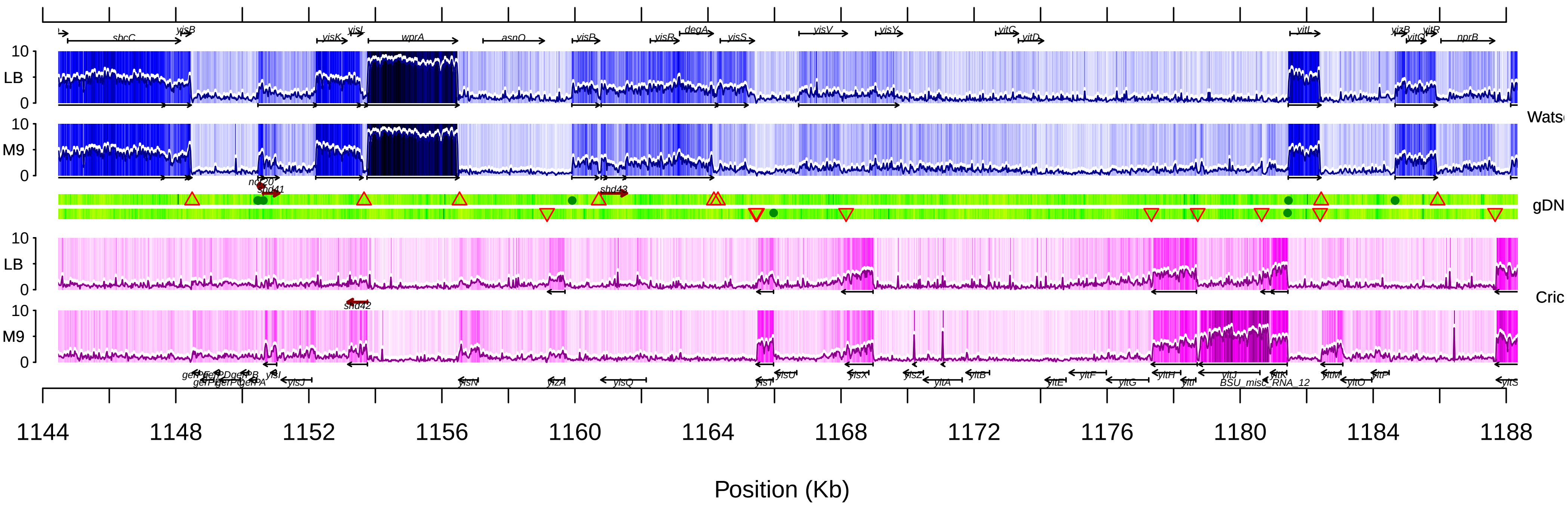

1188 − 1232 Kb

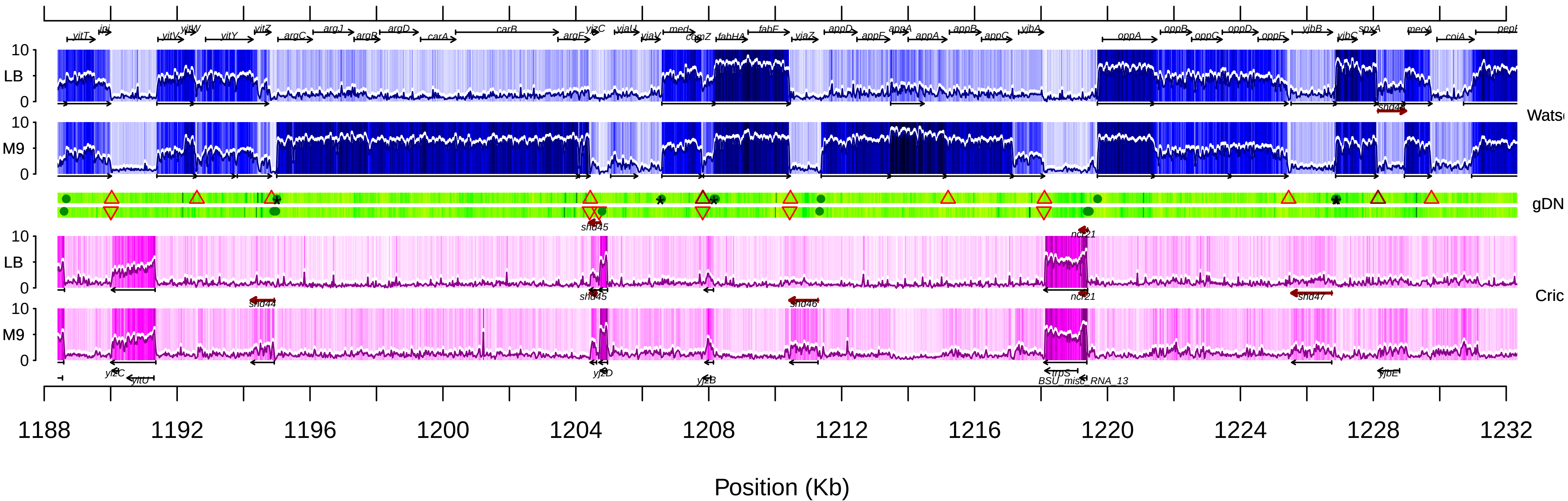

1232 − 1276 Kb

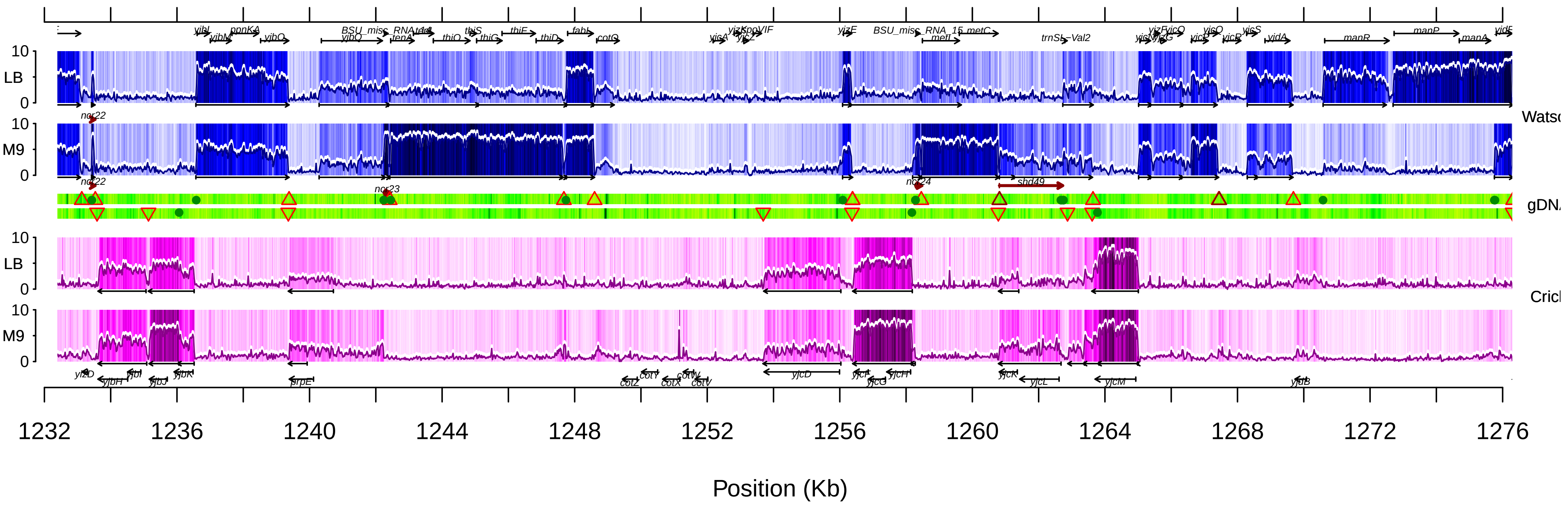

1276 − 1320 Kb

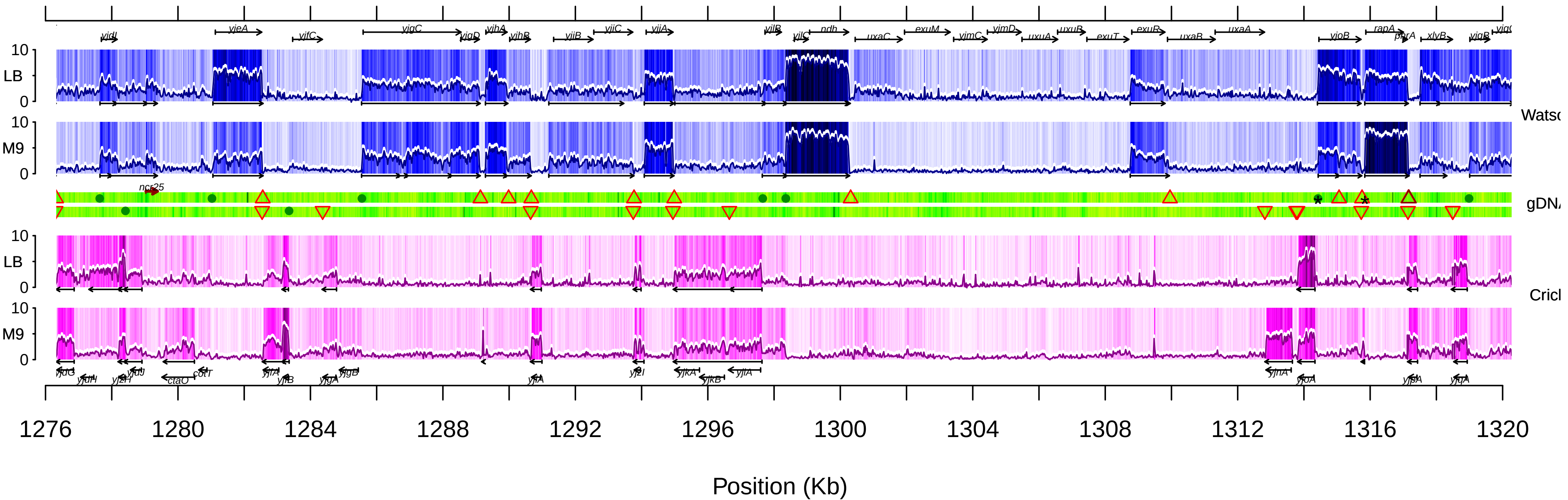

1320 − 1364 Kb

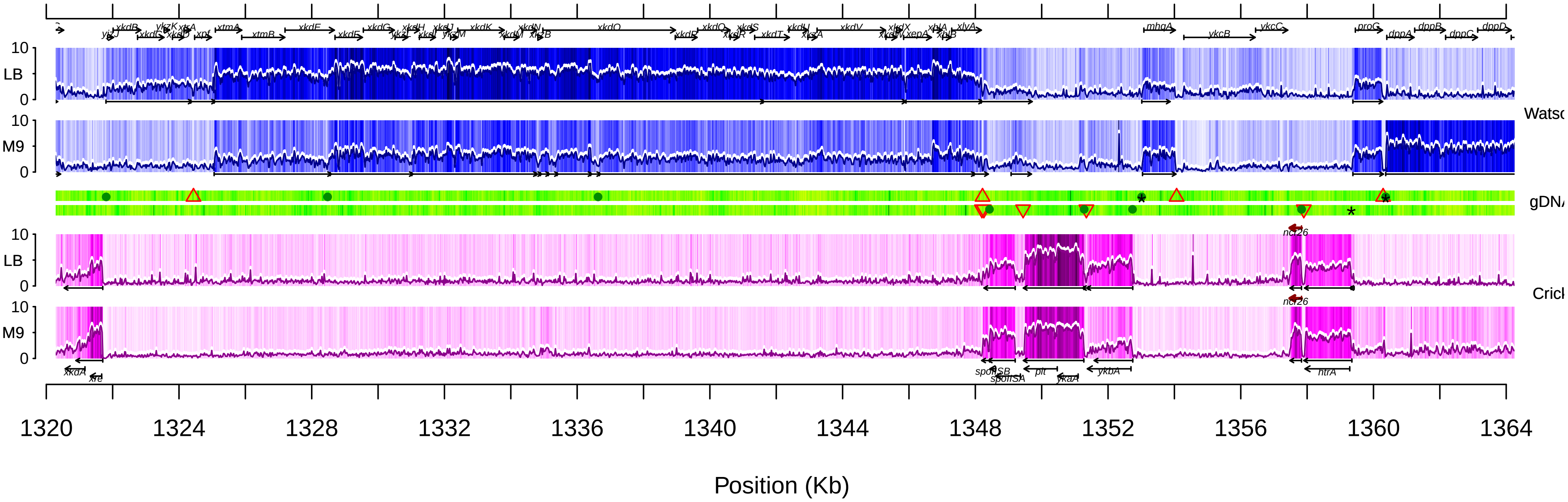

1364 − 1408 Kb

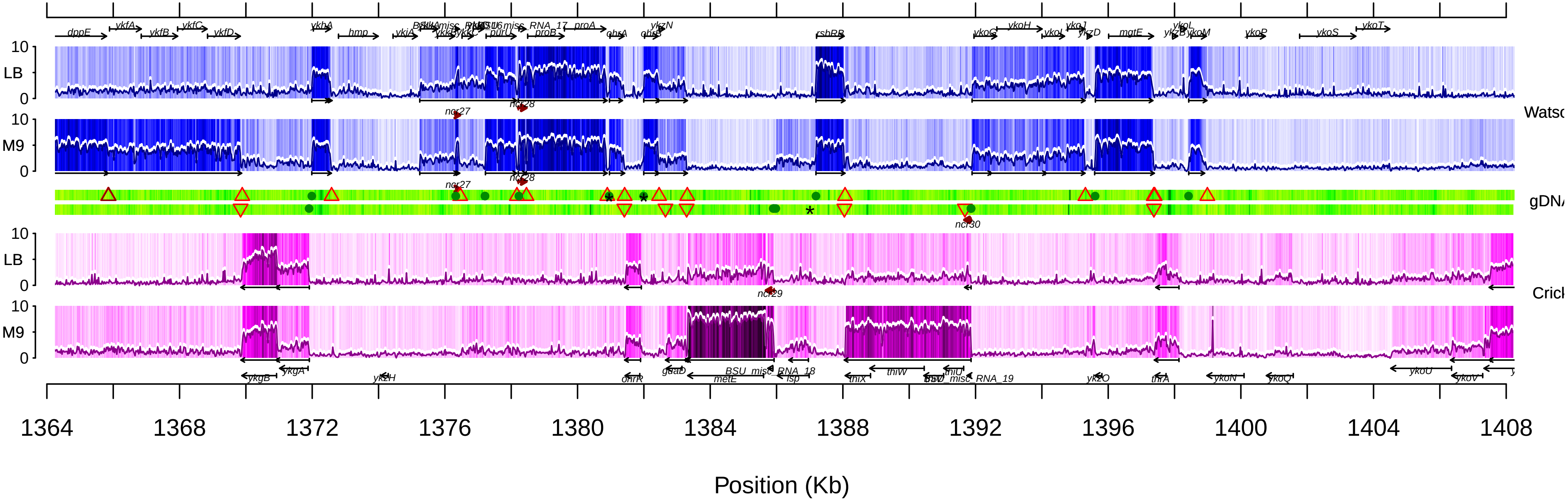

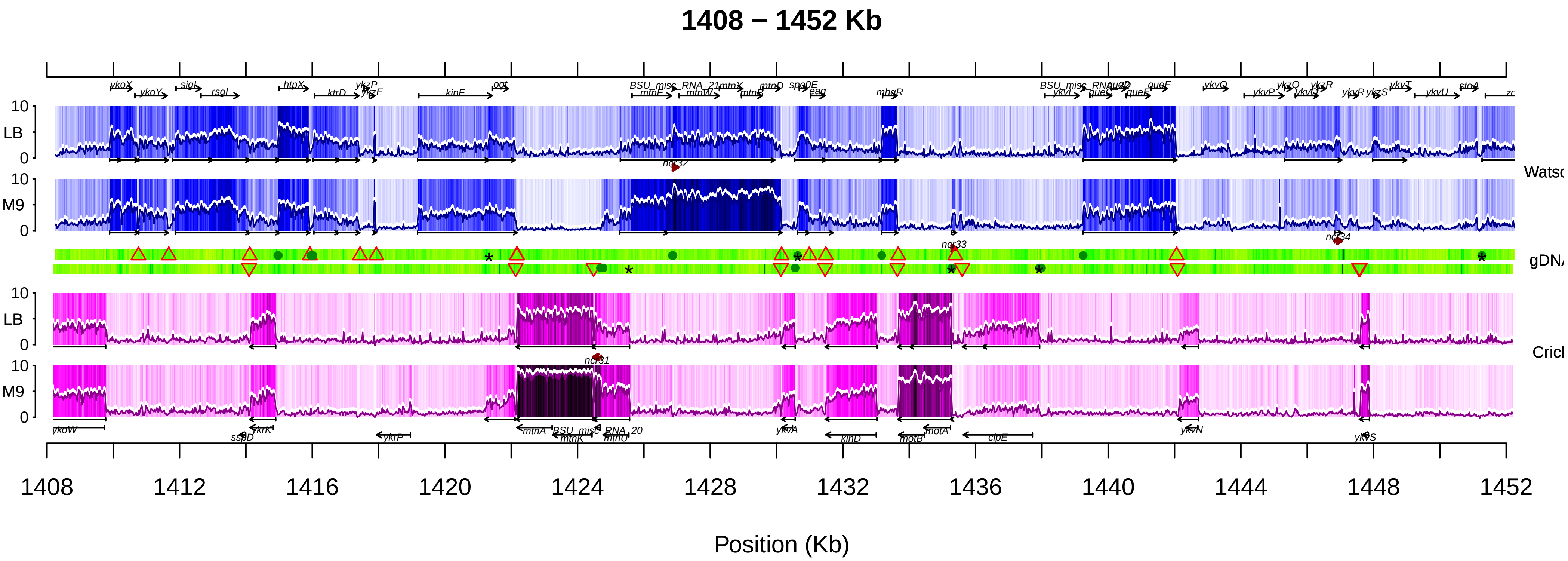

1408 − 1452 Kb
LB
M9
gDNA
Watson
Crick
Position (Kb)
1408
1412
1416
1420
1424
1428
1432
1436
1440
1444
1448
1452

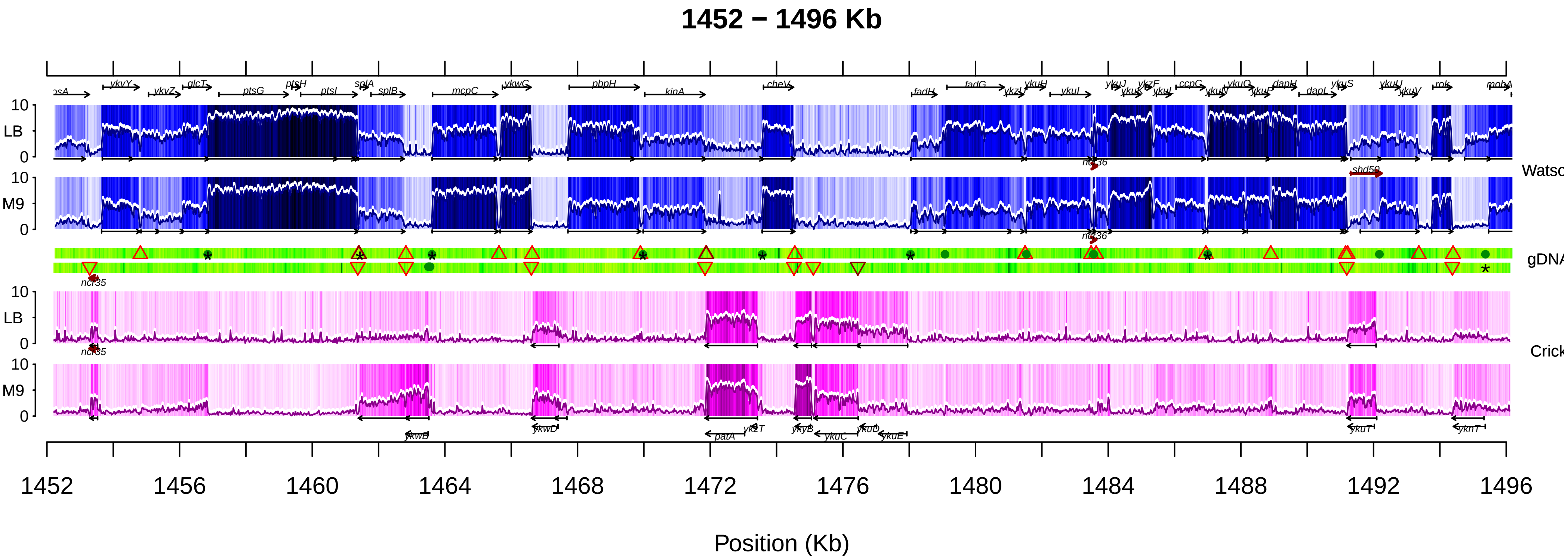

1452 − 1496 Kb
LB
M9
gDNA
Watson
Crick
Position (Kb)
1452
1456
1460
1464
1468
1472
1476
1480
1484
1488
1492
1496

1496 − 1540 Kb

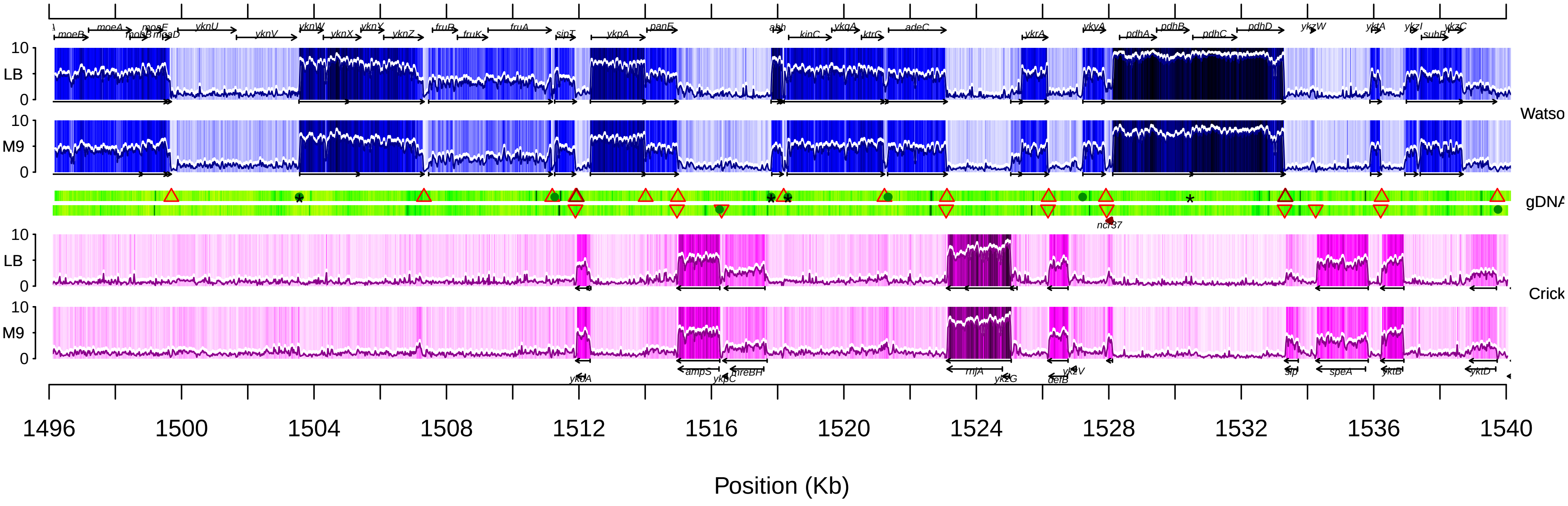

1540 − 1584 Kb

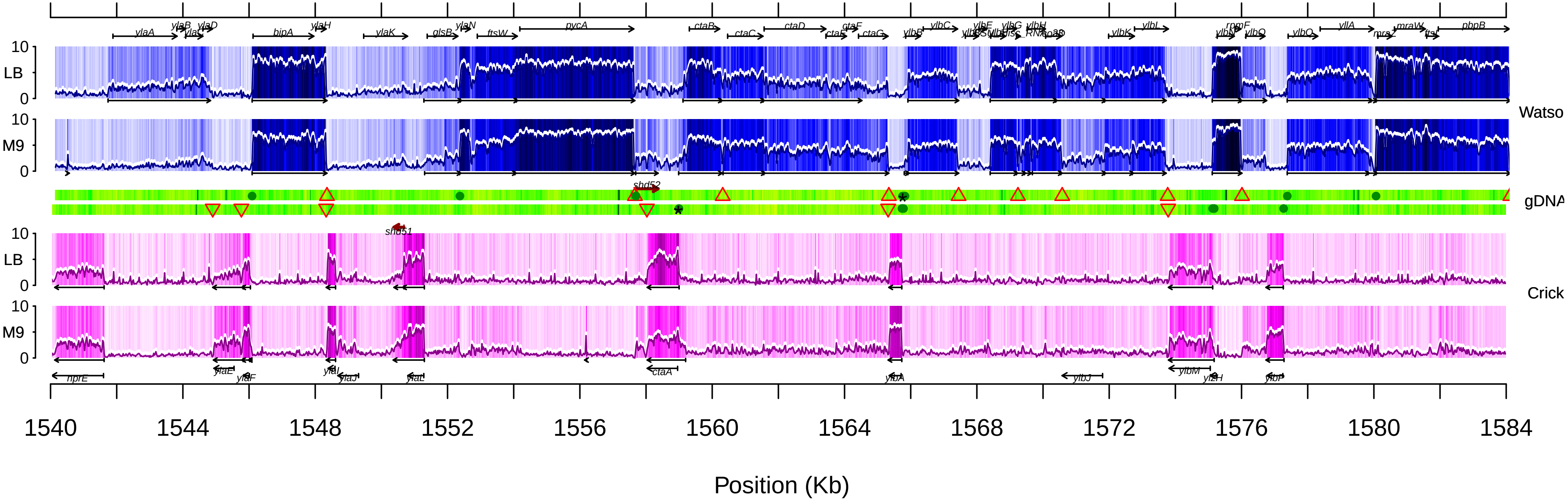

1584 − 1628 Kb

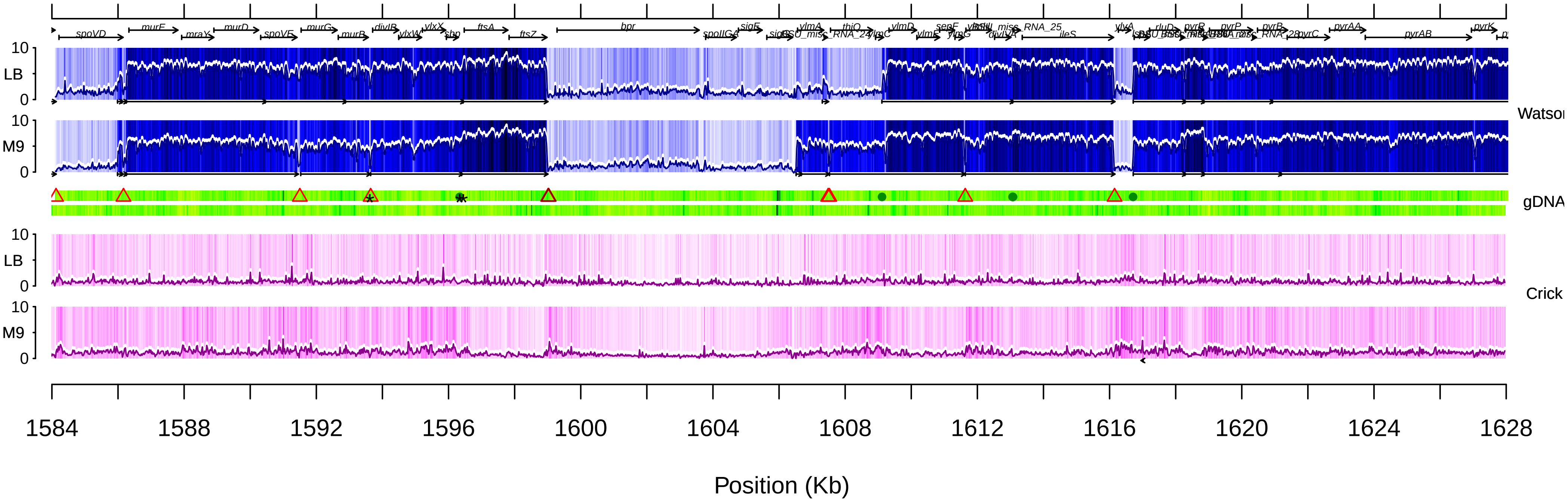

1628 − 1672 Kb

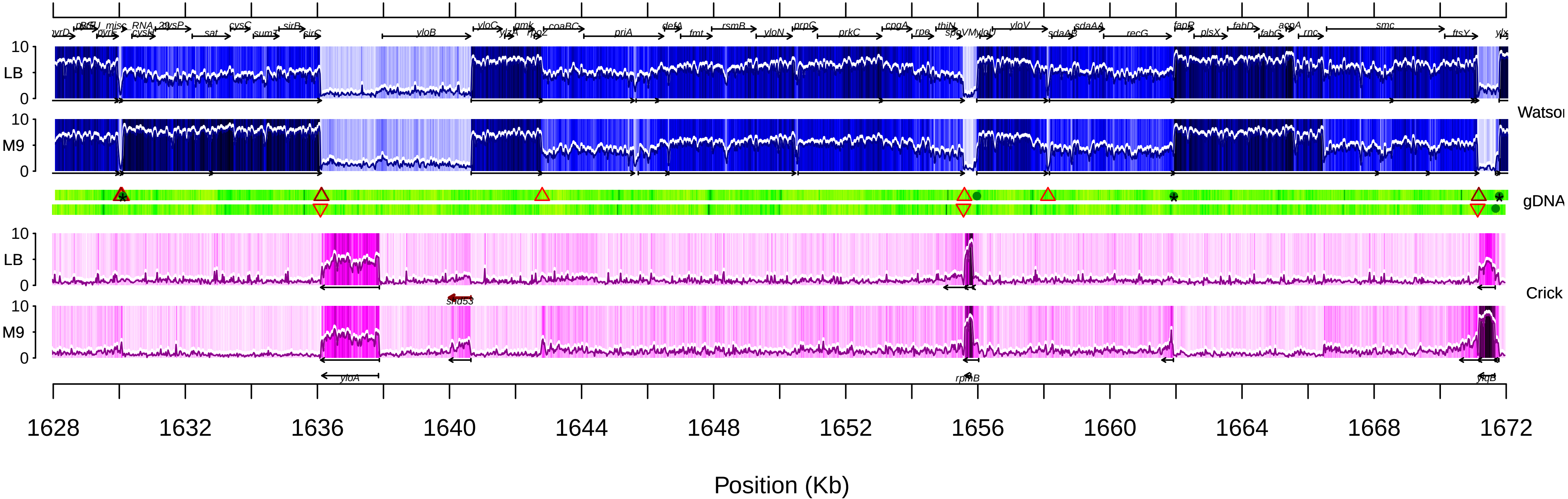

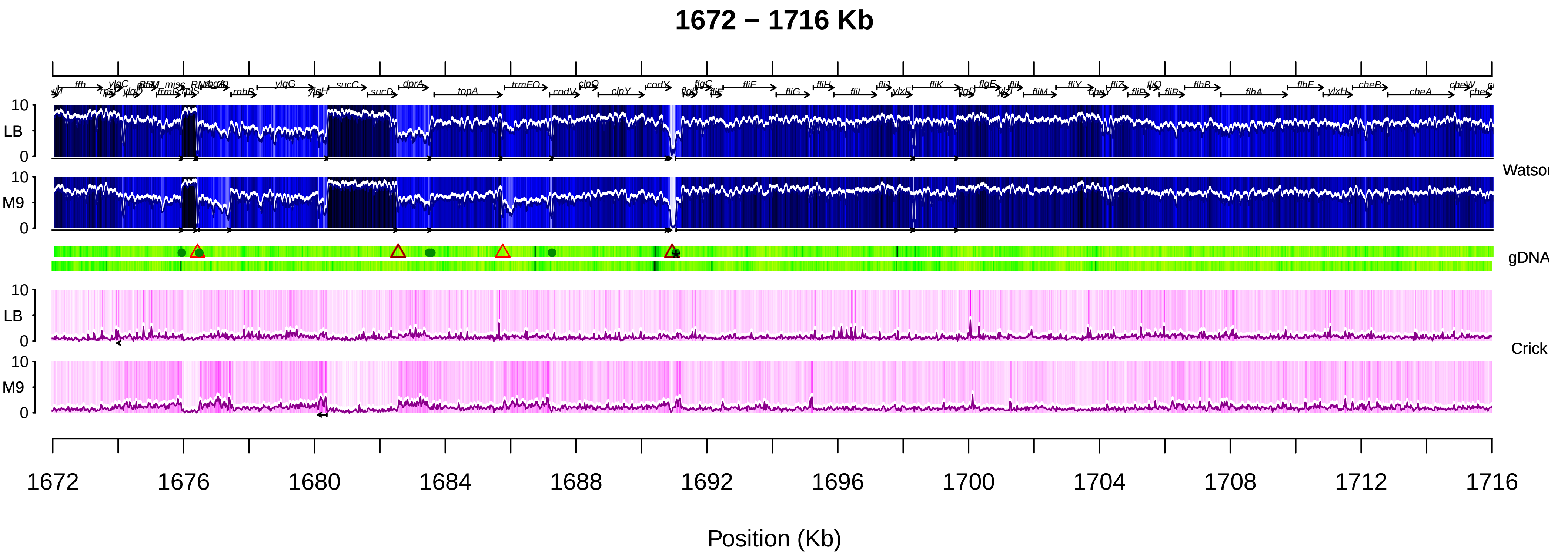
1672 − 1716 Kb
LB
M9
Watson
gDNA
Crick
10
0
1672
1676
1680
1684
1688
1692
1696
1700
1704
1708
1712
1716
Position (Kb)

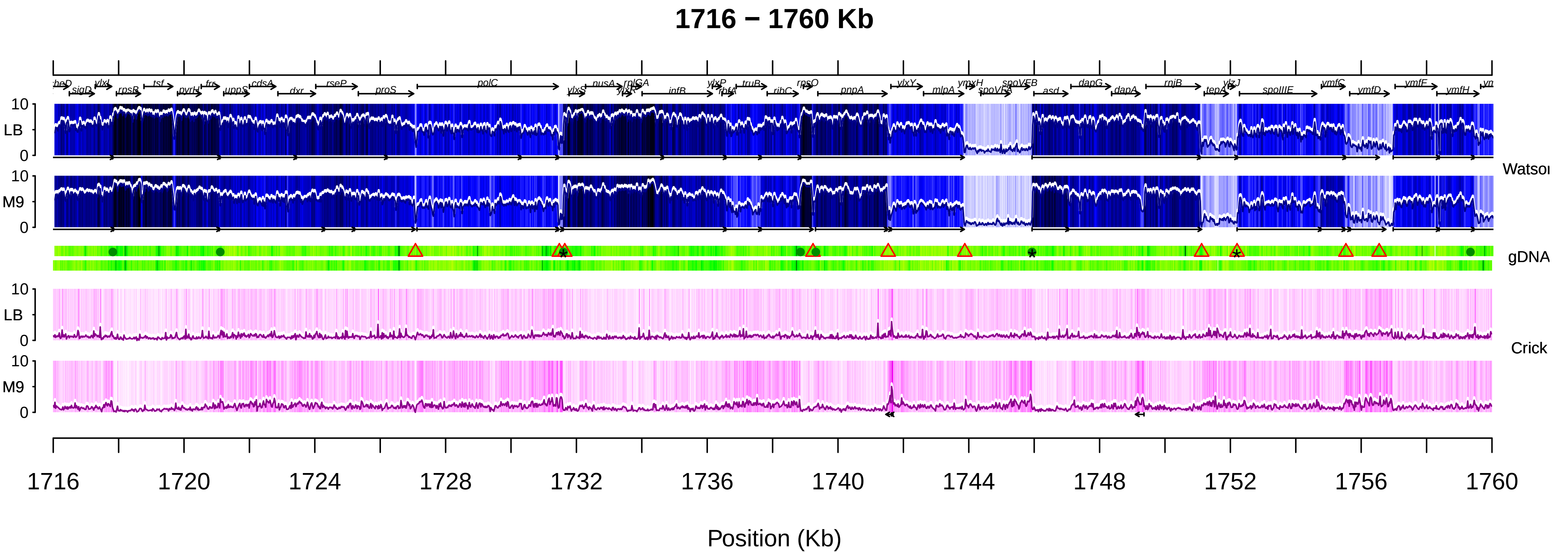
1716 − 1760 Kb
LB
M9
Watson
gDNA
Crick
10
0
1716
1720
1724
1728
1732
1736
1740
1744
1748
1752
1756
1760
Position (Kb)

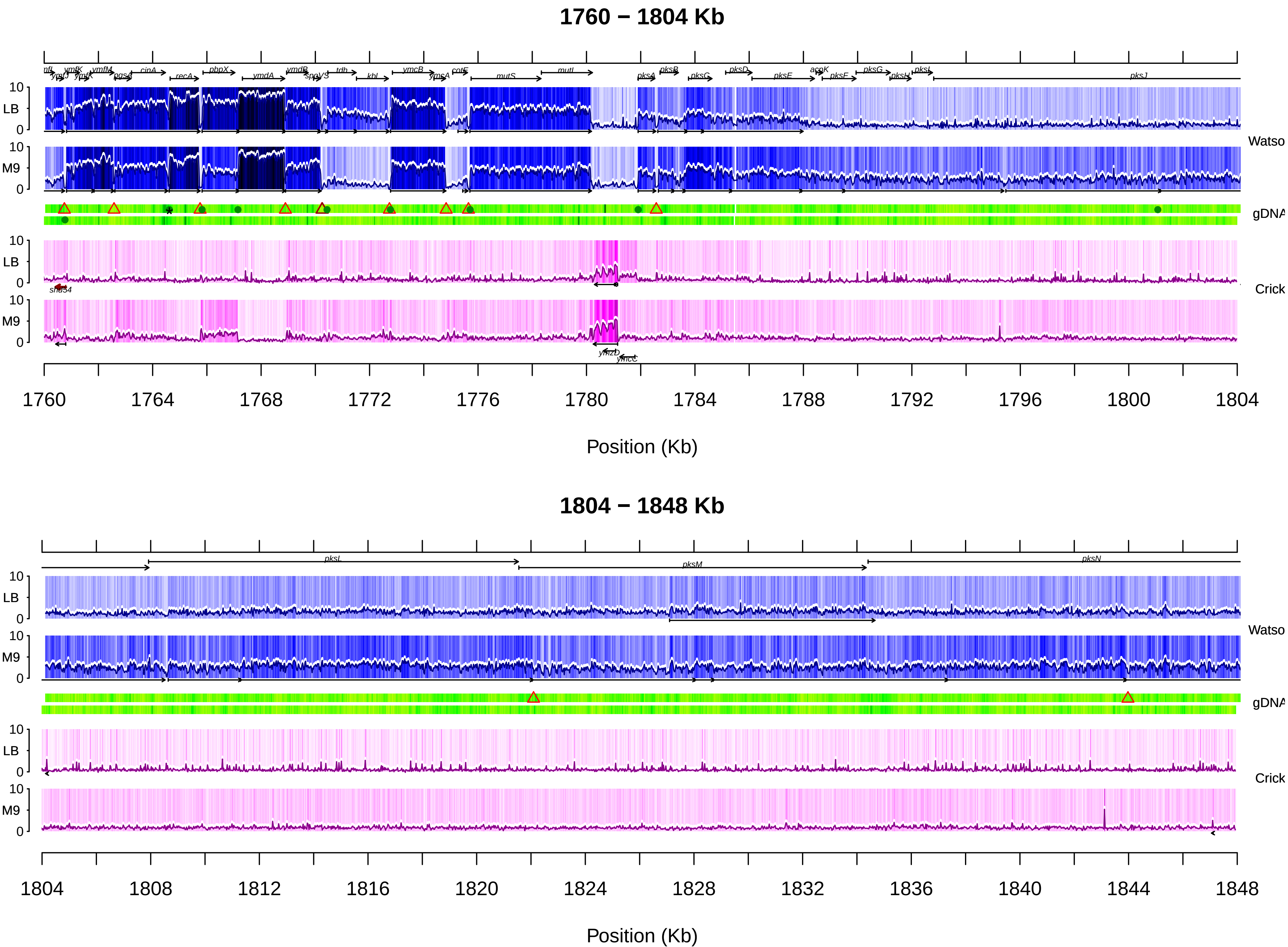

1760 − 1804 Kb
ymfJ
ymfK
ymfM
pgsA
cinA
recA
pbpX
ymdA
ymdB
spoVS
tdh
kbl
ymcB
ymcA
cotE
mutS
mutL
pksA
pksB
pksC
pksD
pksE
acpK
pksF
pksG
pksH
pksI
pksJ
10
LB
0
10
M9
0
Watso
gDNA
Crick
ymcC
1760
1764
1768
1772
1776
1780
1784
1788
1792
1796
1800
1804
Position (Kb)
1804 − 1848 Kb
pksL
pksM
pksN
10
LB
0
10
M9
0
Watso
gDNA
Crick
1804
1808
1812
1816
1820
1824
1828
1832
1836
1840
1844
1848
Position (Kb)

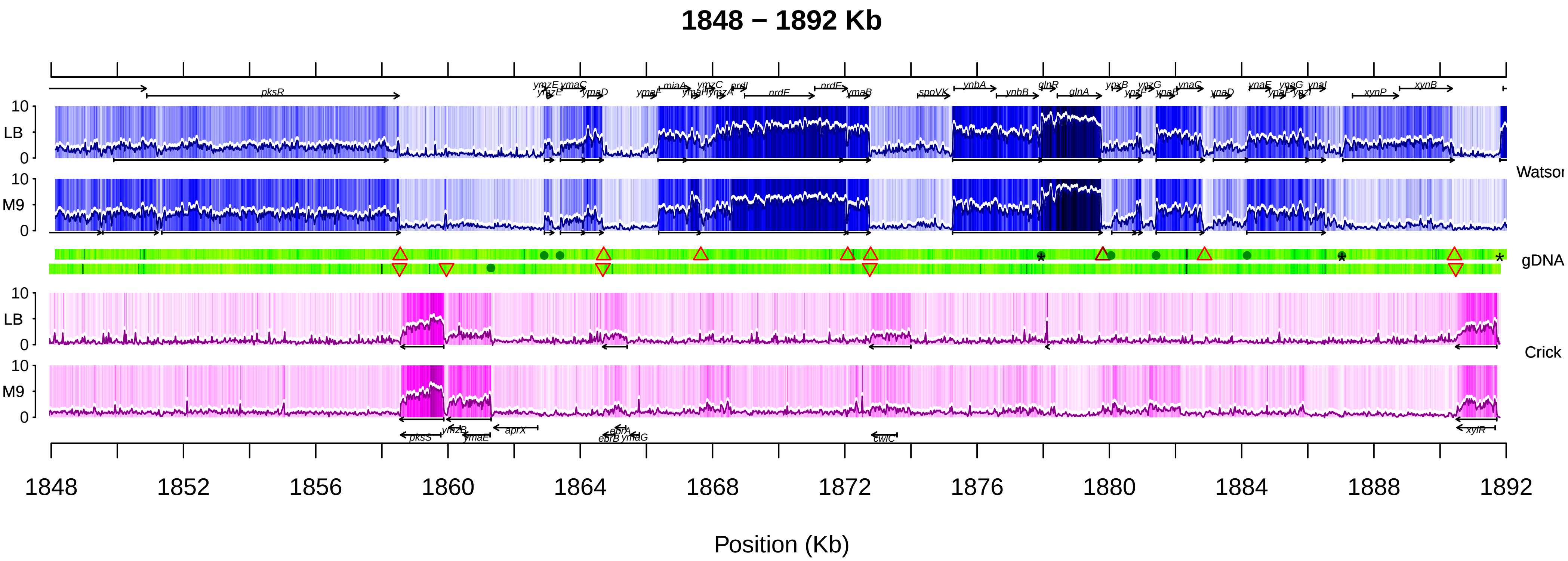
1848 − 1892 Kb
10
LB
0
10
M9
0
Watson
gDNA
Crick
pksR
1848
1852
1856
1860
1864
1868
1872
1876
1880
1884
1888
1892
Position (Kb)

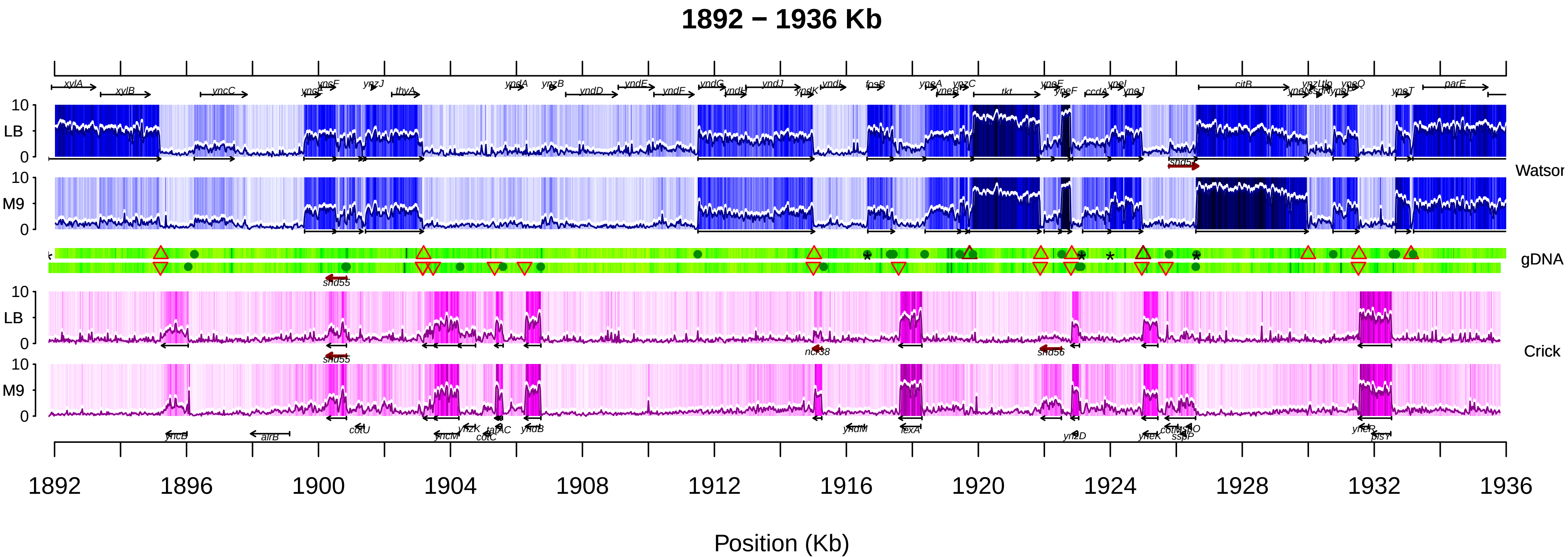
1892 − 1936 Kb
10
LB
0
10
M9
0
Watson
gDNA
Crick
1892
1896
1900
1904
1908
1912
1916
1920
1924
1928
1932
1936
Position (Kb)

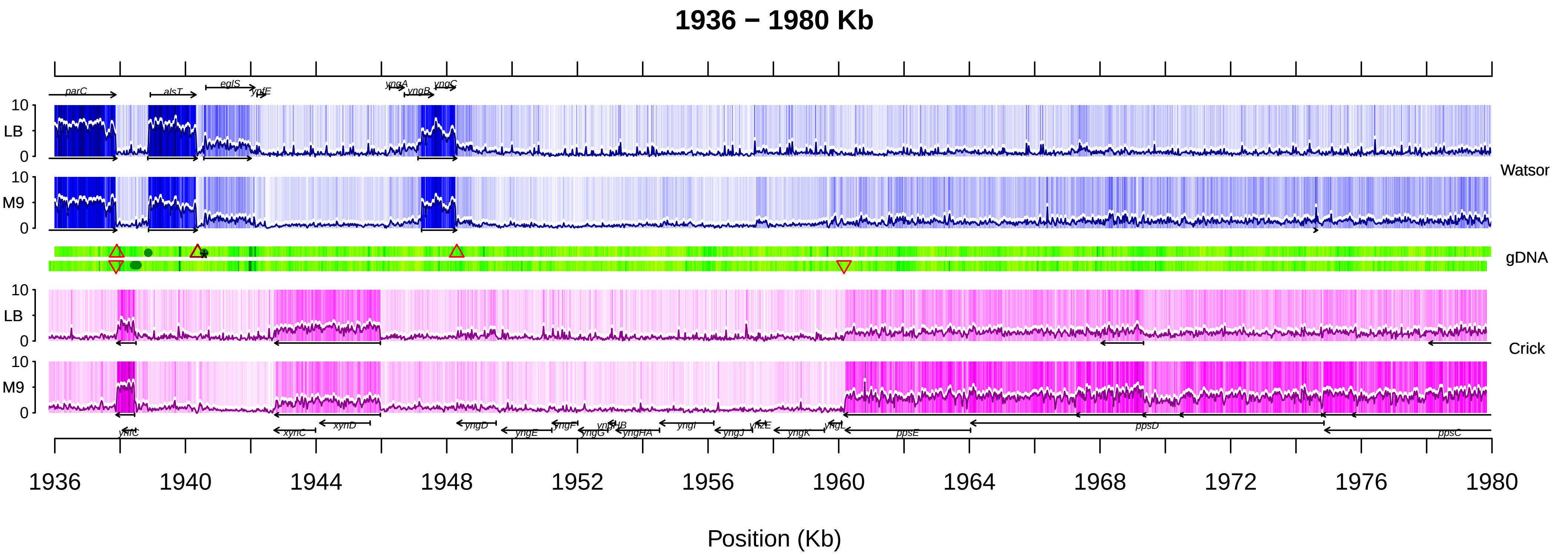

1936 − 1980 Kb
parC
alsT
eglS
yqfE
yngA
yngB
yngC
10
LB
0
10
M9
0
Watsor
gDNA
Crick
ynfC
xynC
xynD
yngD
yngE
yngF
yngG
yngHB
yngHA
yngI
yngJ
ynzE
yngK
yngL
ppsE
ppsD
ppsC
1936
1940
1944
1948
1952
1956
1960
1964
1968
1972
1976
1980
Position (Kb)

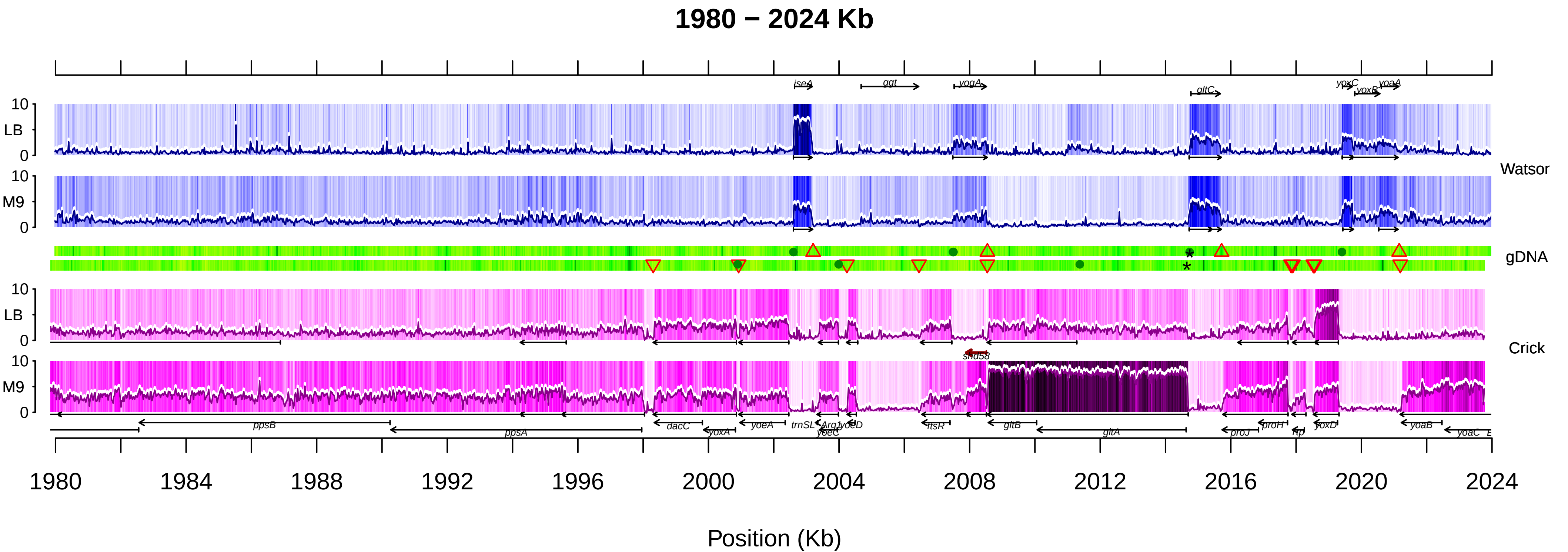

1980 − 2024 Kb
iseA
ggt
yogA
gltC
yoxC
yoxB
yoaA
10
LB
0
10
M9
0
Watsor
gDNA
Crick
snd58
ppsB
ppsA
dacC
yoxA
yoeA
trnSL-Arg1
yoeC
yoeD
ftsR
gltB
gltA
proJ
proH
yoxD
yoaB
yoaC
1980
1984
1988
1992
1996
2000
2004
2008
2012
2016
2020
2024
Position (Kb)

2024 − 2068 Kb

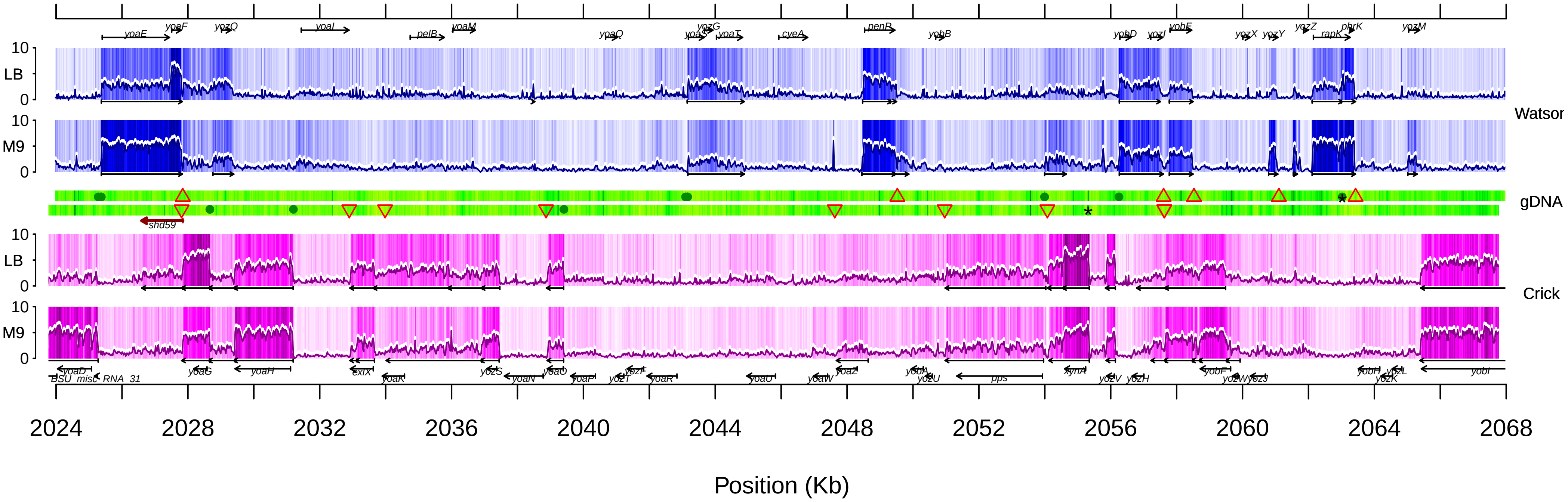

2068 − 2112 Kb

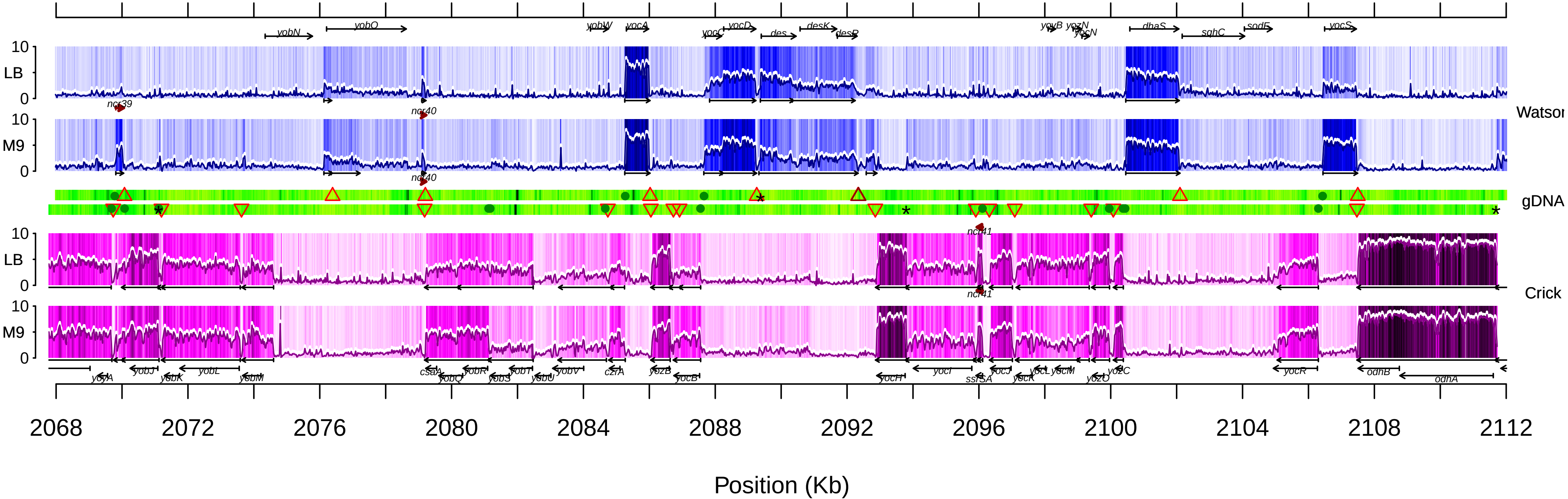

2112 – 2156 Kb

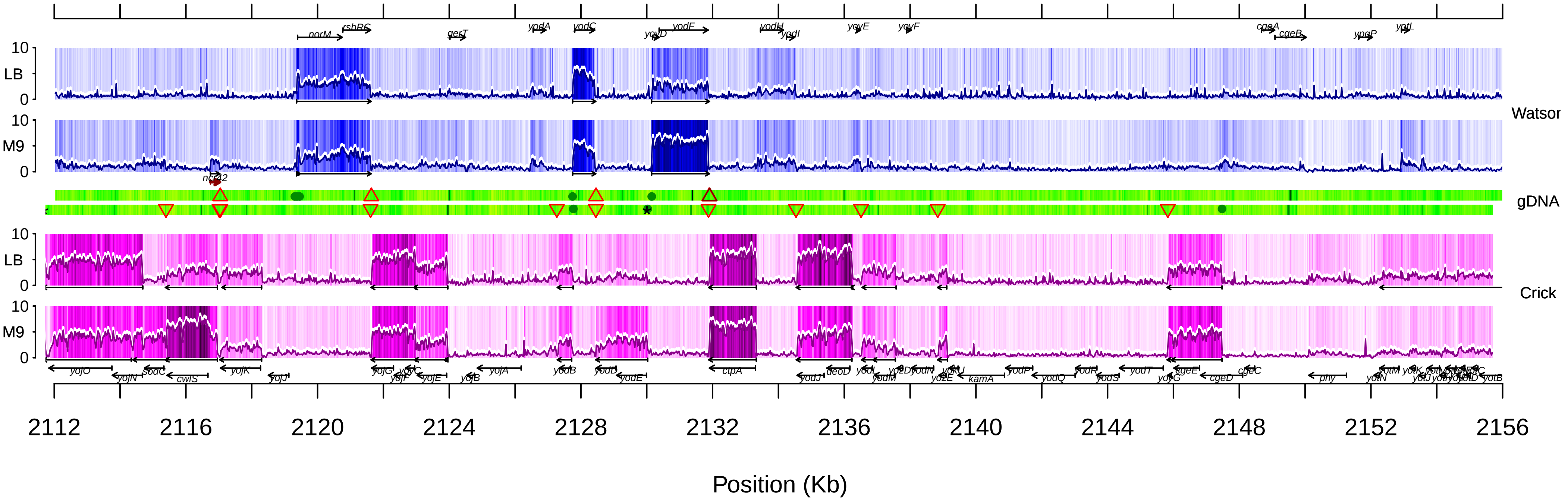

2156 – 2200 Kb

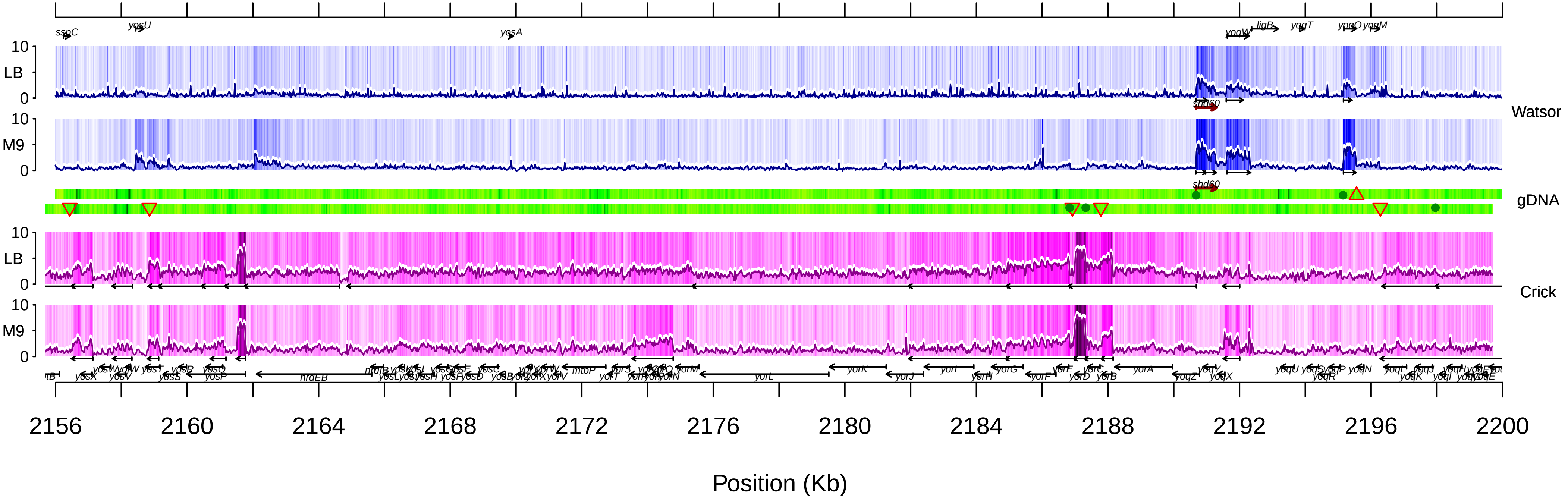

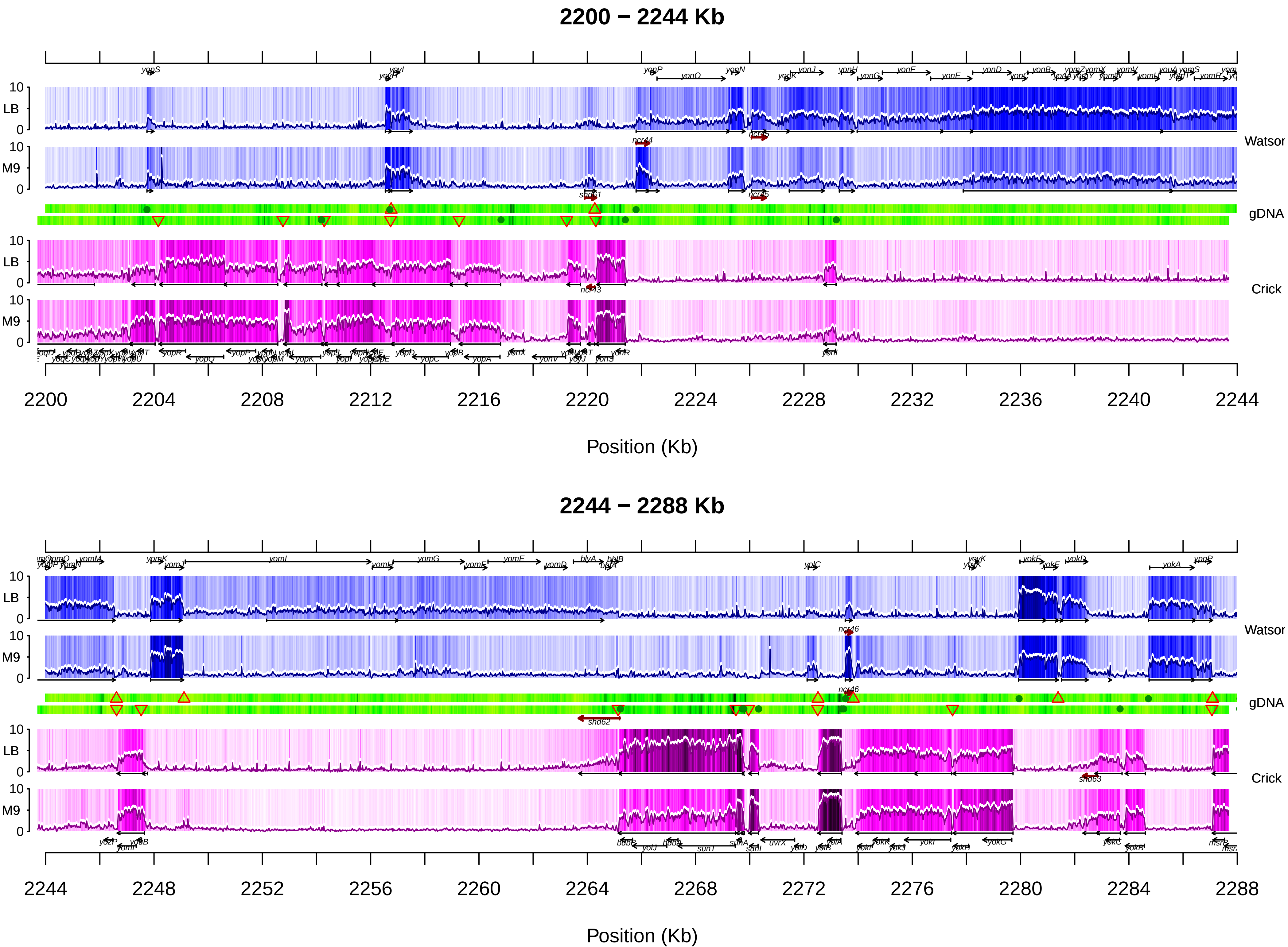
2200 – 2244 Kb
LB
M9
Watson
gDNA
Crick
2200
2204
2208
2212
2216
2220
2224
2228
2232
2236
2240
2244
Position (Kb)
2244 – 2288 Kb
LB
M9
Watson
gDNA
Crick
2244
2248
2252
2256
2260
2264
2268
2272
2276
2280
2284
2288
Position (Kb)

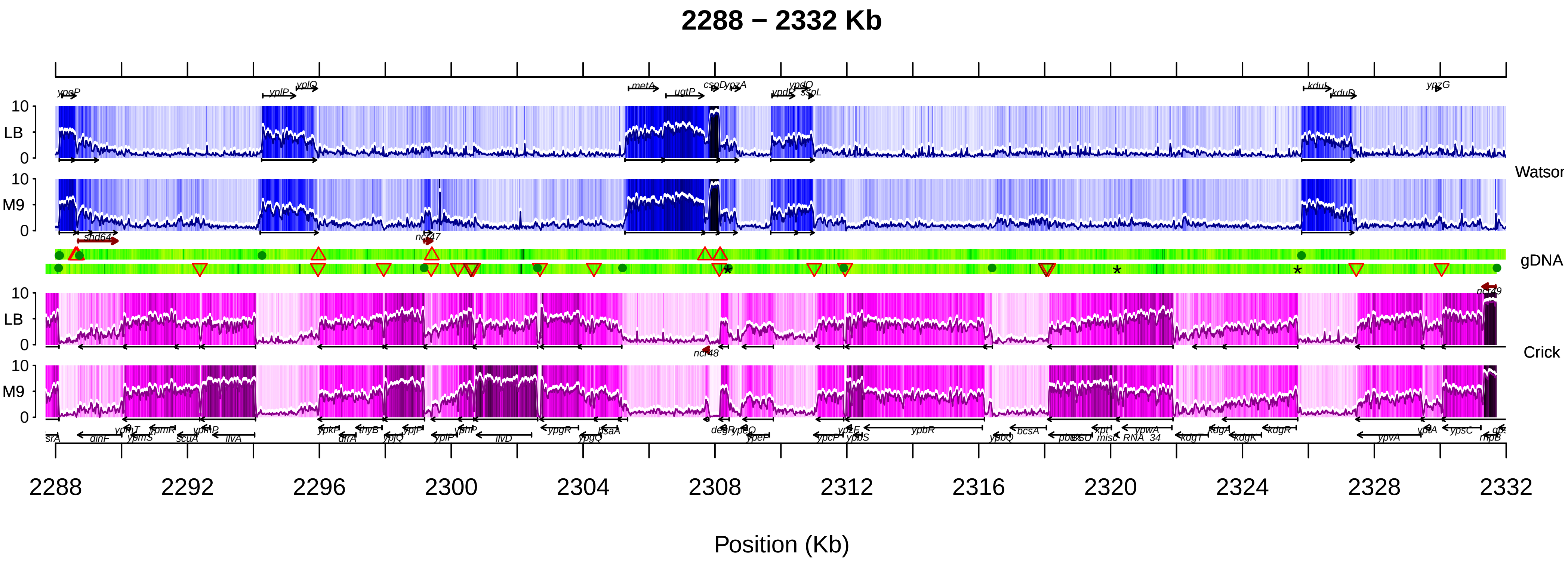

2288 – 2332 Kb
LB
M9
Watsor
gDNA
Crick
2288
2292
2296
2300
2304
2308
2312
2316
2320
2324
2328
2332
Position (Kb)

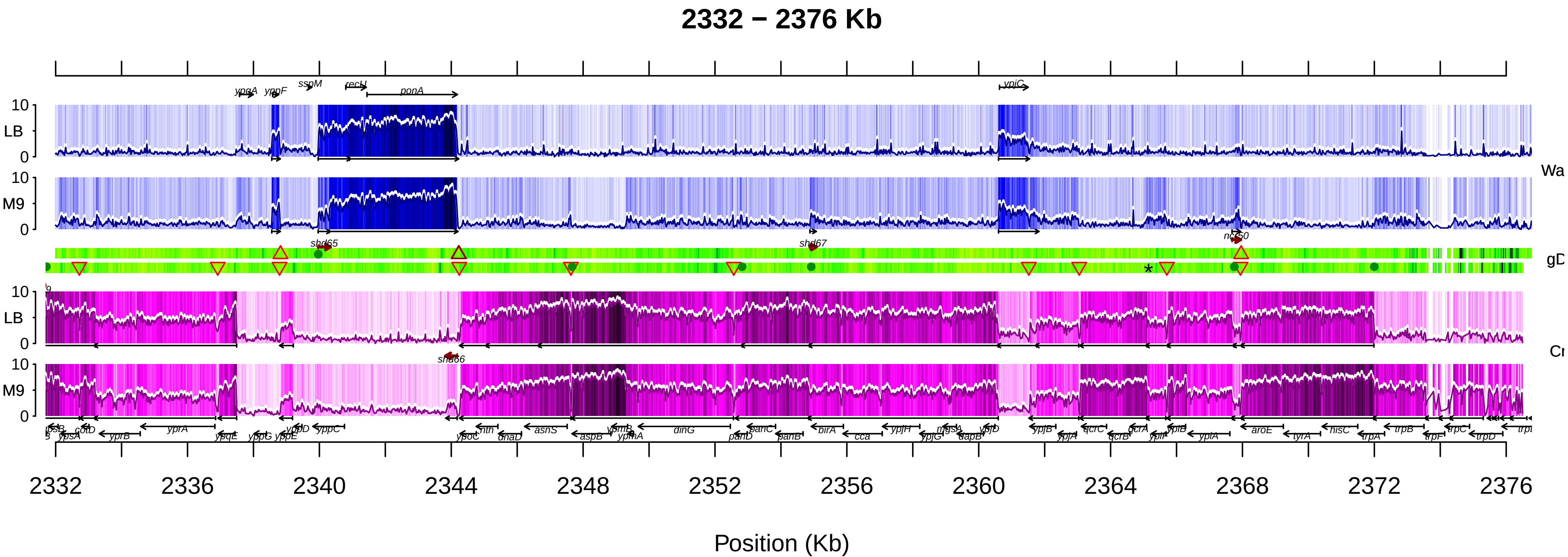

2332 – 2376 Kb
LB
M9
Wa
gD
Cr
2332
2336
2340
2344
2348
2352
2356
2360
2364
2368
2372
2376
Position (Kb)

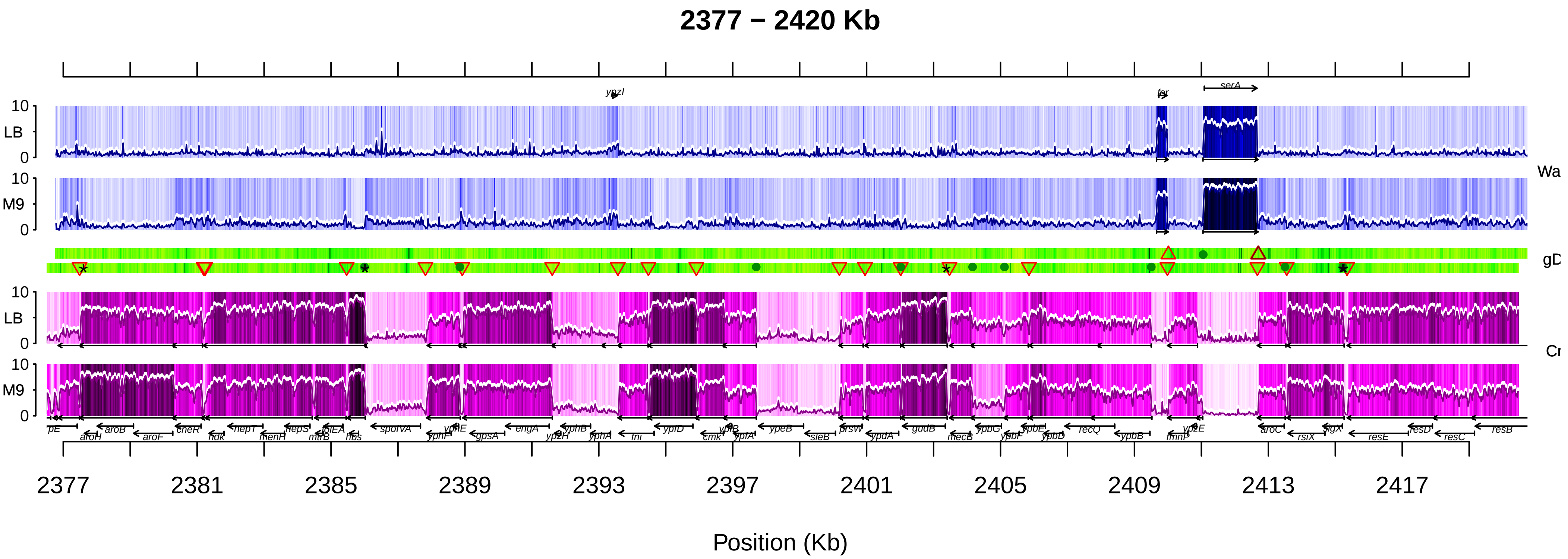
2377 − 2420 Kb
10
LB
0
10
M9
0
Wa
gD
Cr
2377
2381
2385
2389
2393
2397
2401
2405
2409
2413
2417
Position (Kb)

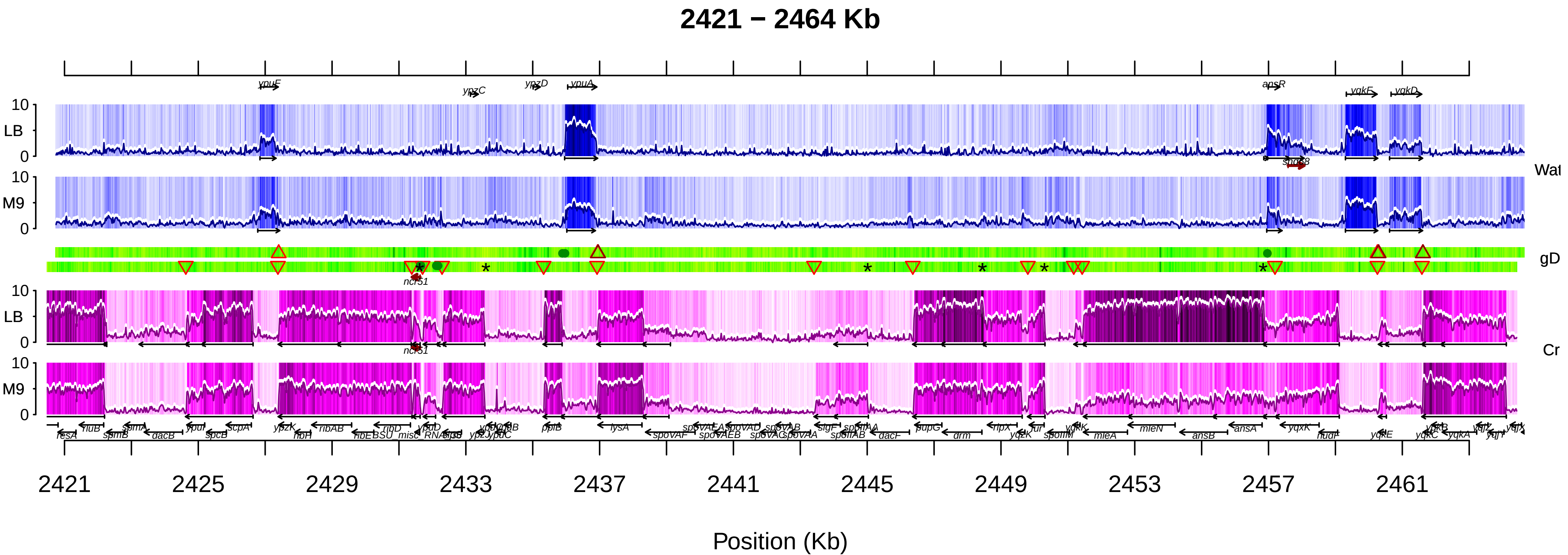
2421 − 2464 Kb
10
LB
0
10
M9
0
Wat
gD
Cr
2421
2425
2429
2433
2437
2441
2445
2449
2453
2457
2461
Position (Kb)

2465 – 2508 Kb

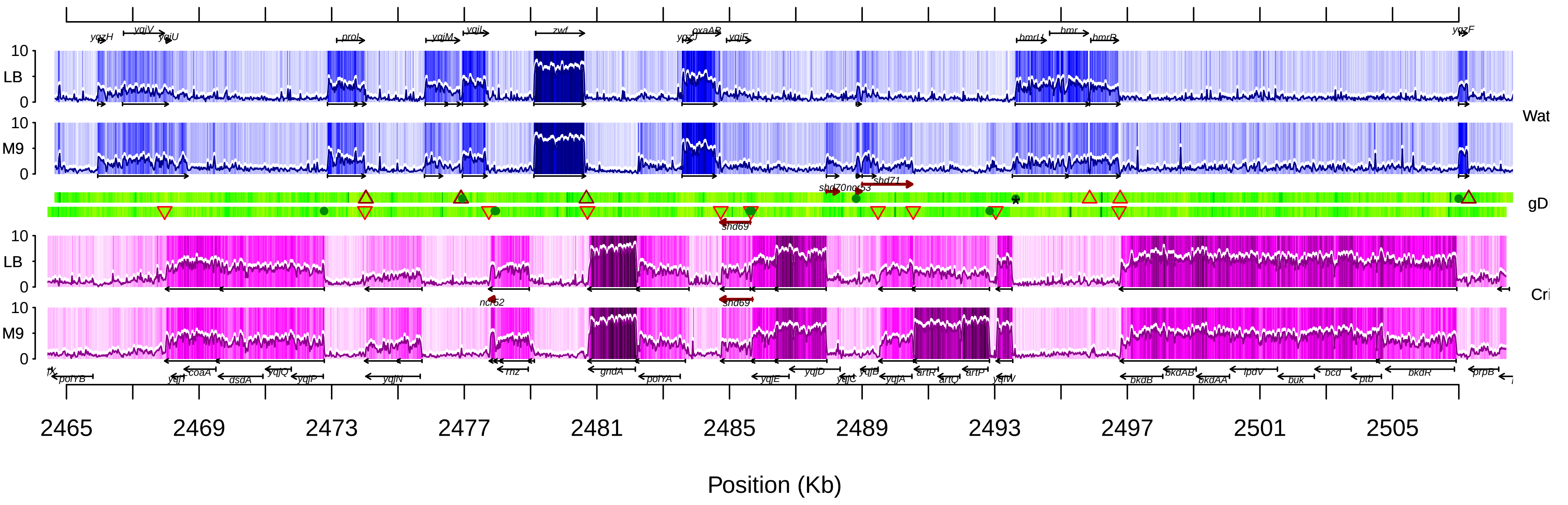

2509 – 2552 Kb

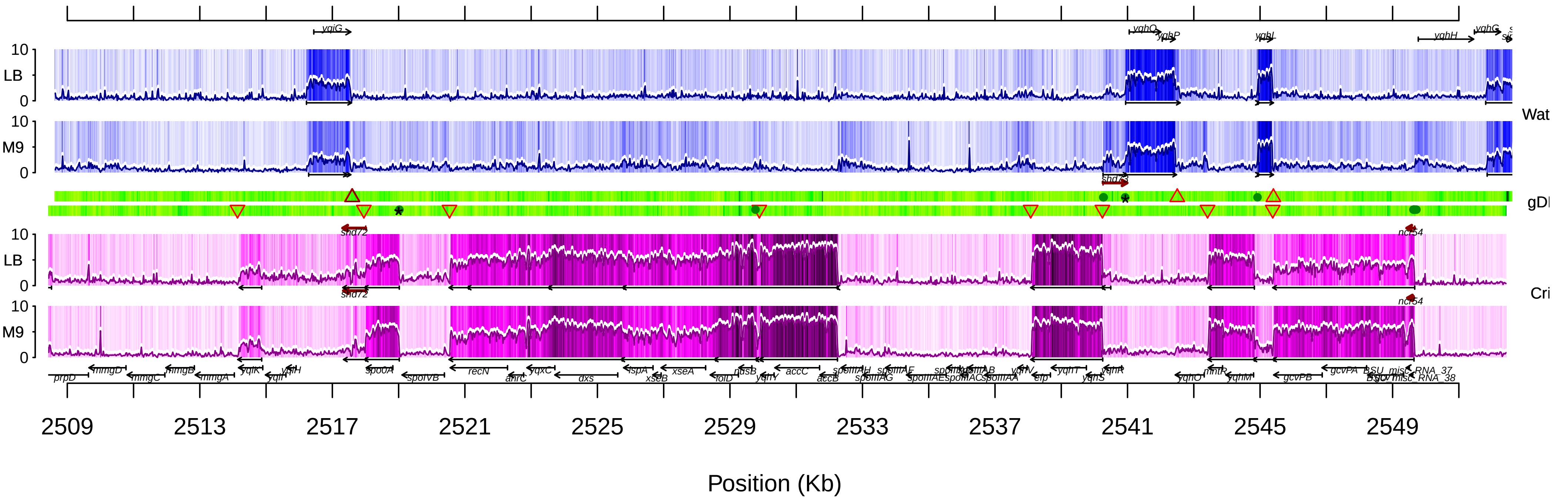

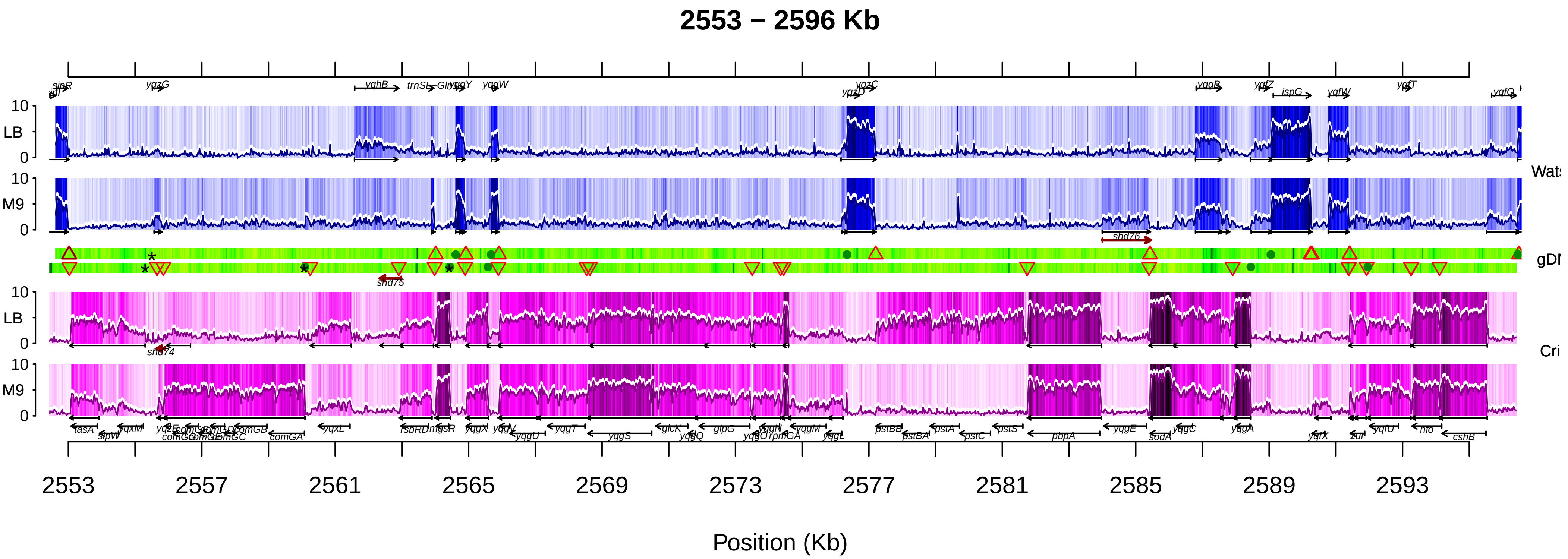
2553 – 2596 Kb
LB
M9
Wats
gDN
Cri
2553
2557
2561
2565
2569
2573
2577
2581
2585
2589
2593
Position (Kb)

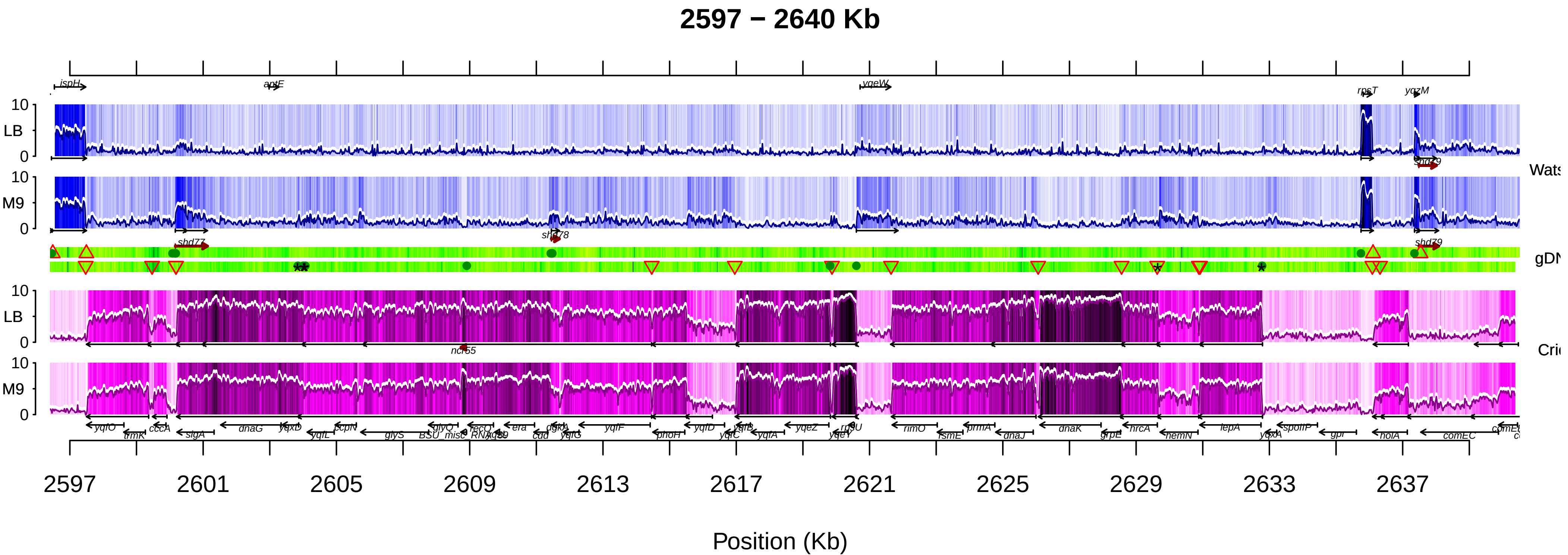
2597 – 2640 Kb
LB
M9
Wats
gDN
Cri
2597
2601
2605
2609
2613
2617
2621
2625
2629
2633
2637
Position (Kb)

2641 − 2684 Kb

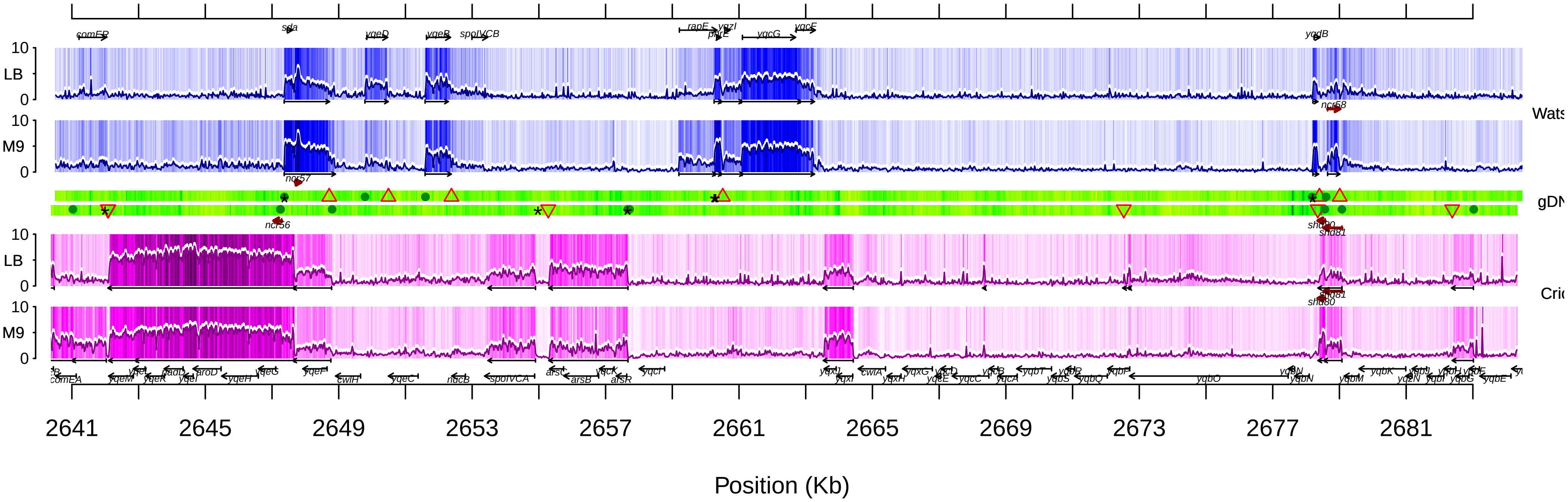

2685 − 2728 Kb

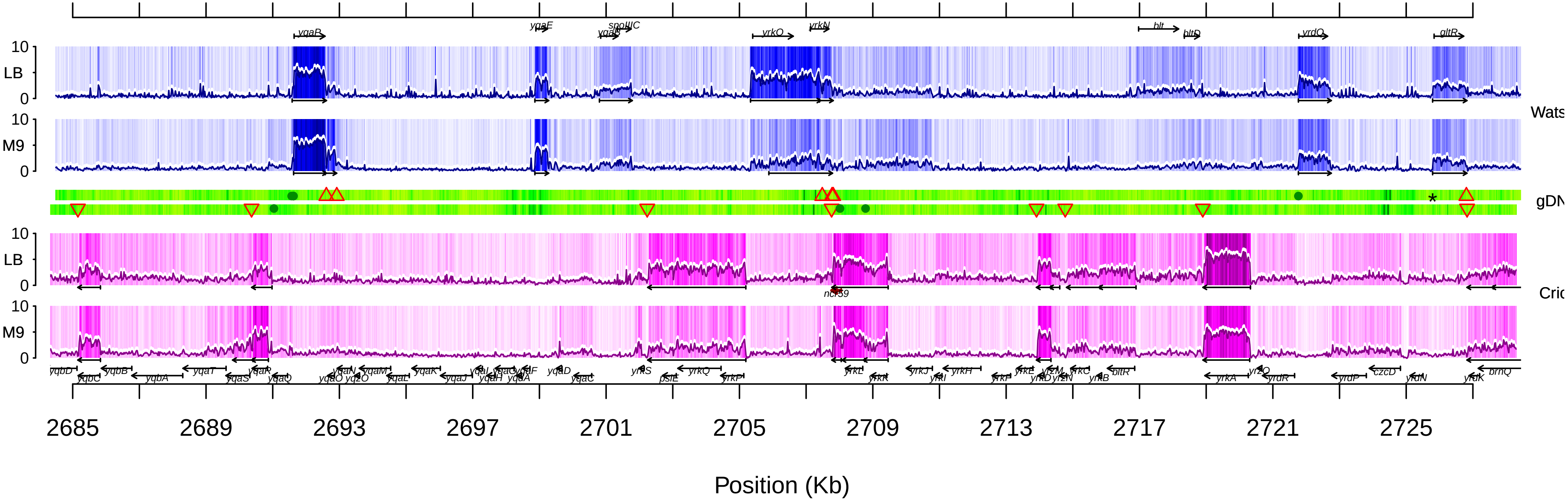

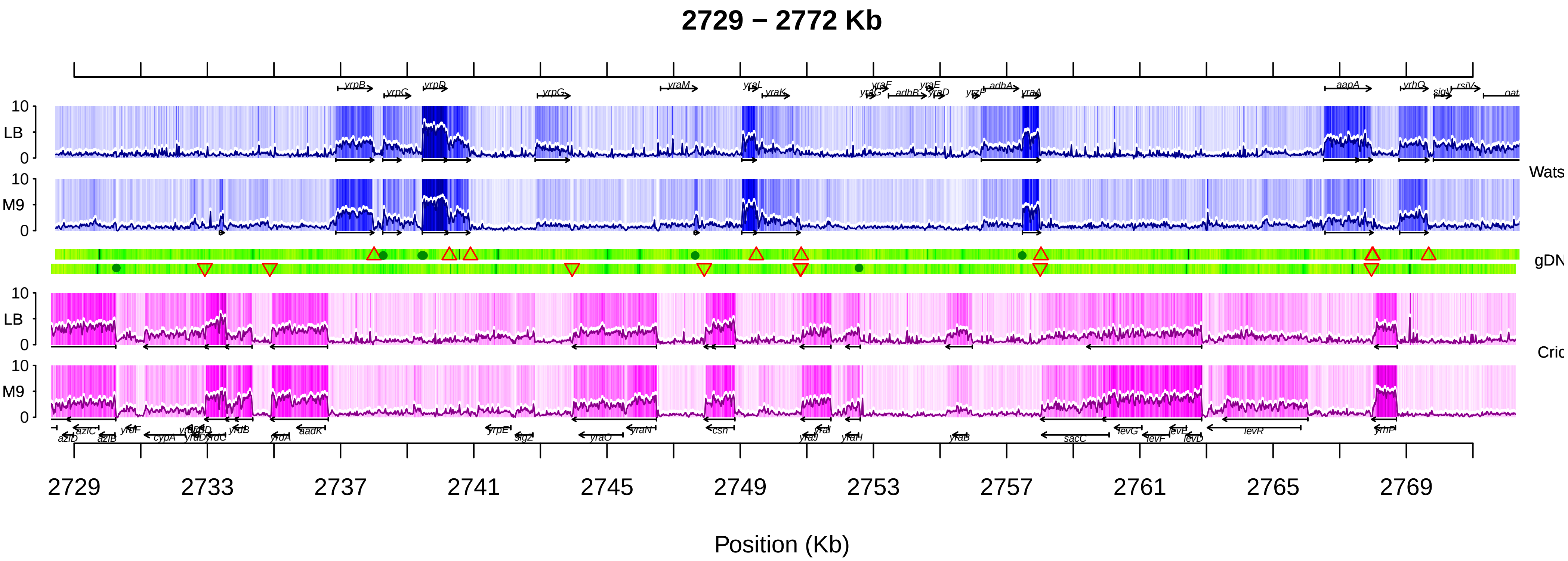
2729 − 2772 Kb
10
LB
0
10
M9
0
Wats
gDN
10
LB
0
Cric
10
M9
0
2729
2733
2737
2741
2745
2749
2753
2757
2761
2765
2769
Position (Kb)

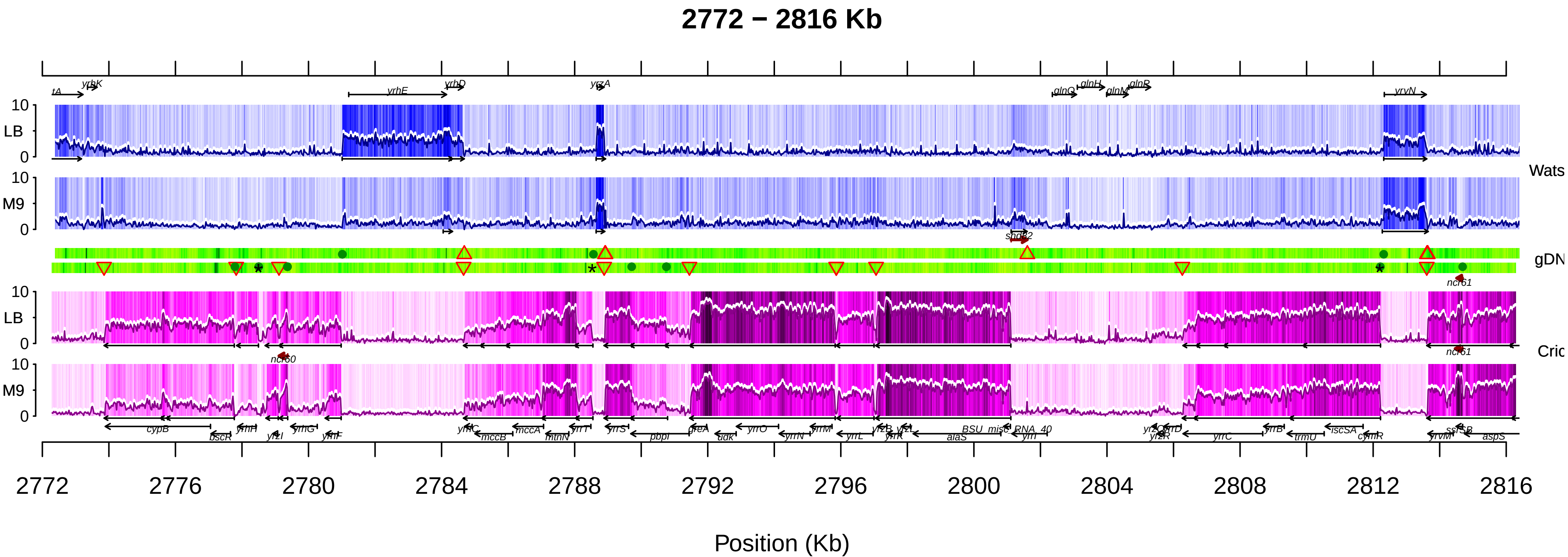
2772 − 2816 Kb
10
LB
0
10
M9
0
Wats
gDN
10
LB
0
Cric
10
M9
0
2772
2776
2780
2784
2788
2792
2796
2800
2804
2808
2812
2816
Position (Kb)

2816 − 2860 Kb

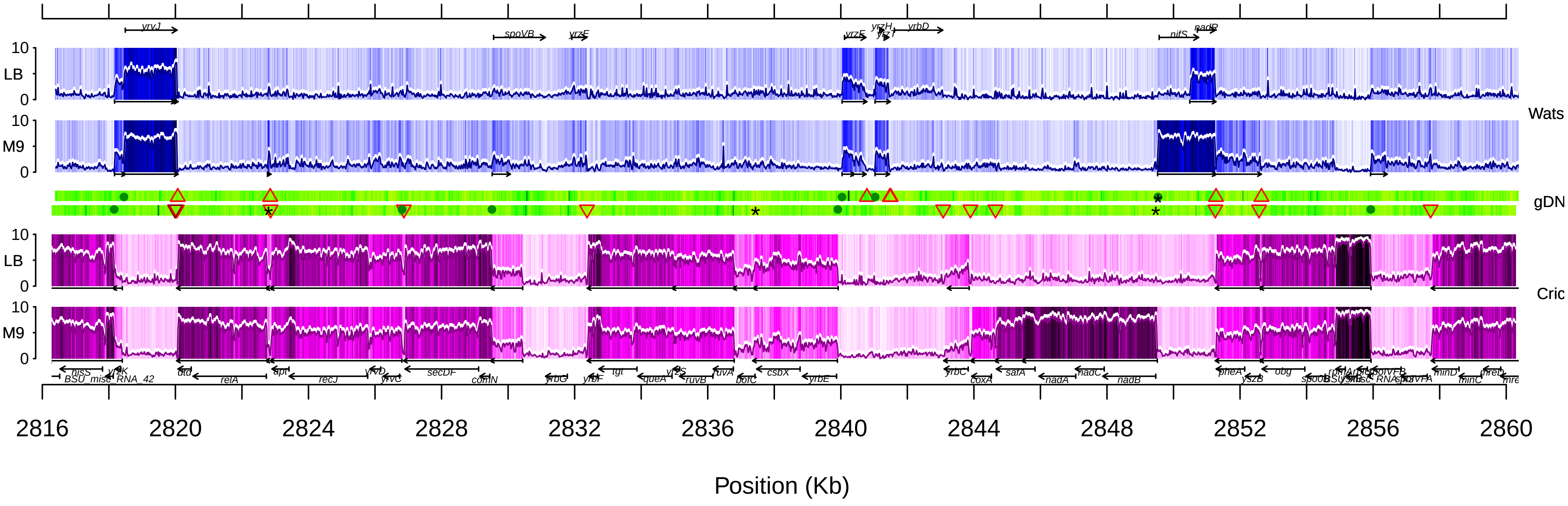

2860 − 2904 Kb

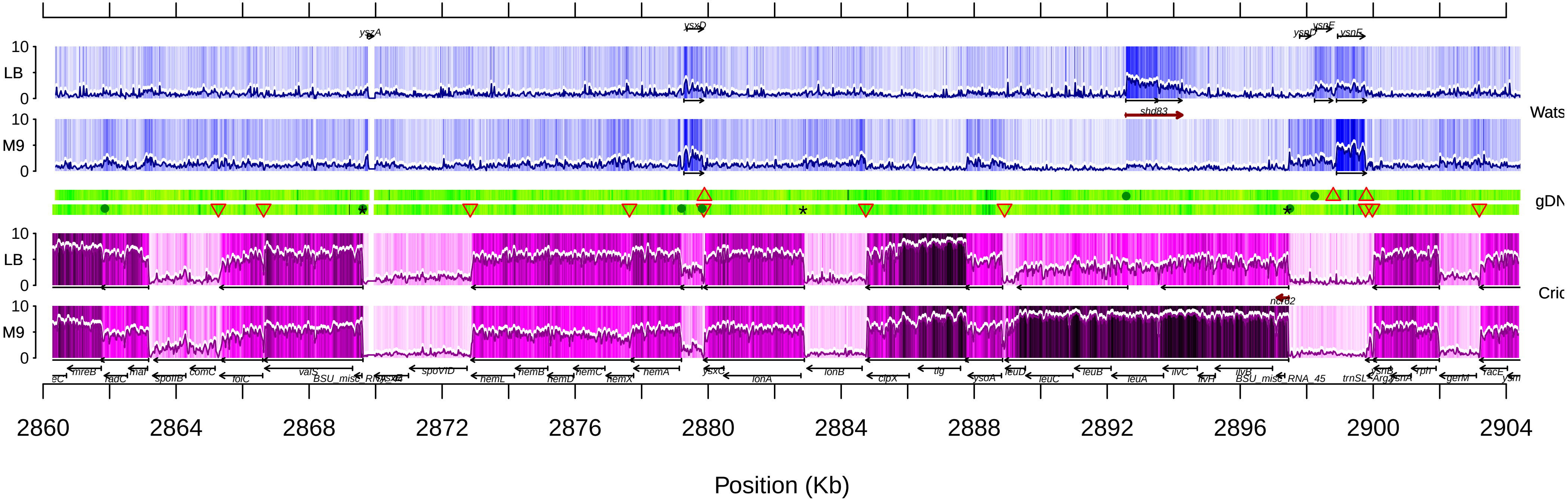

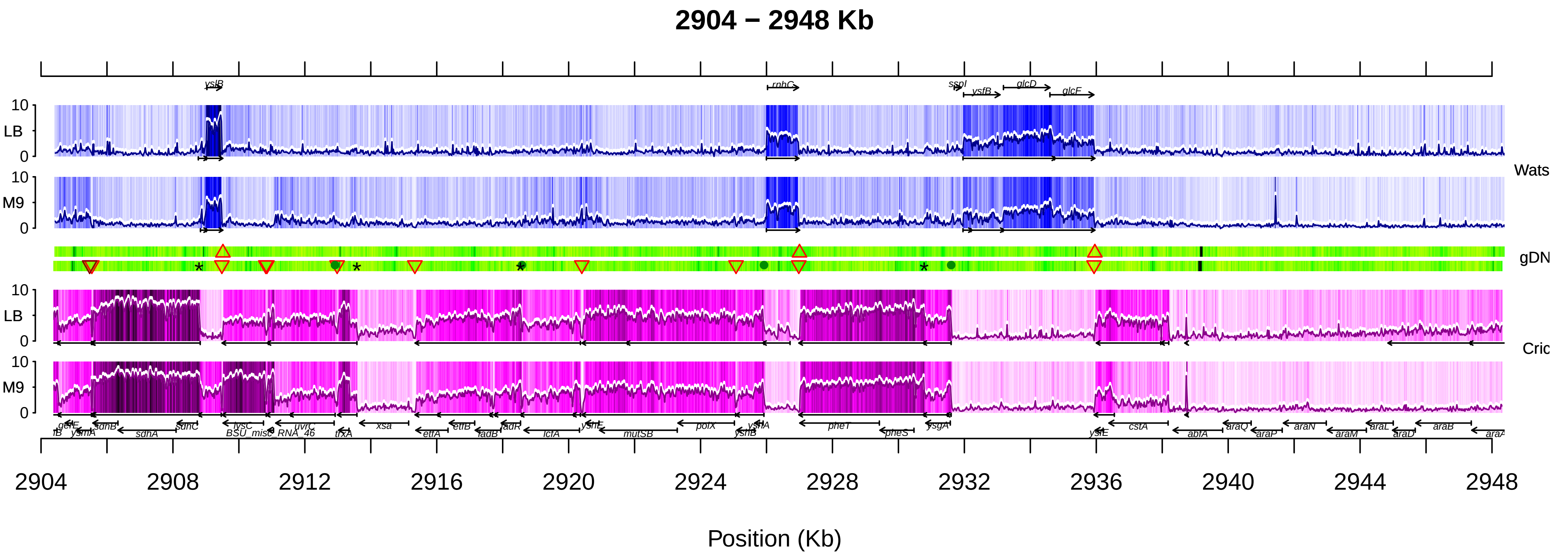
2904 – 2948 Kb
LB
M9
Wats
gDN
Cric
Position (Kb)

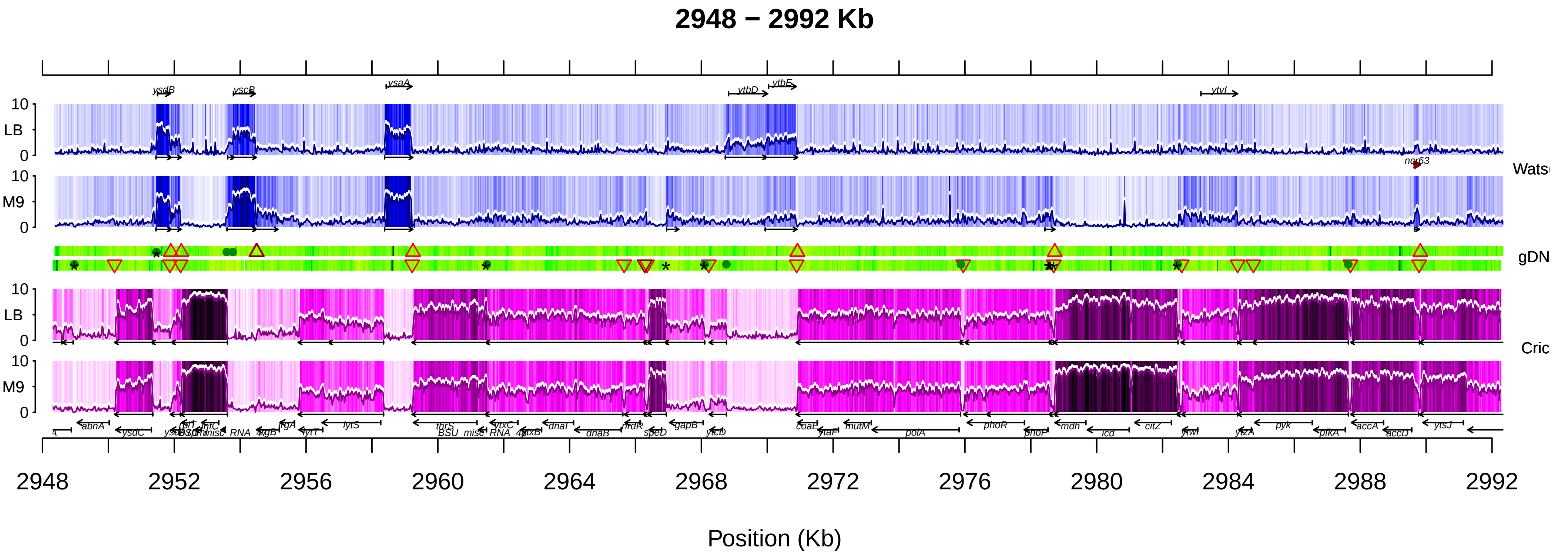
2948 – 2992 Kb
LB
M9
Wats
gDN
Cric
Position (Kb)

2992 − 3036 Kb

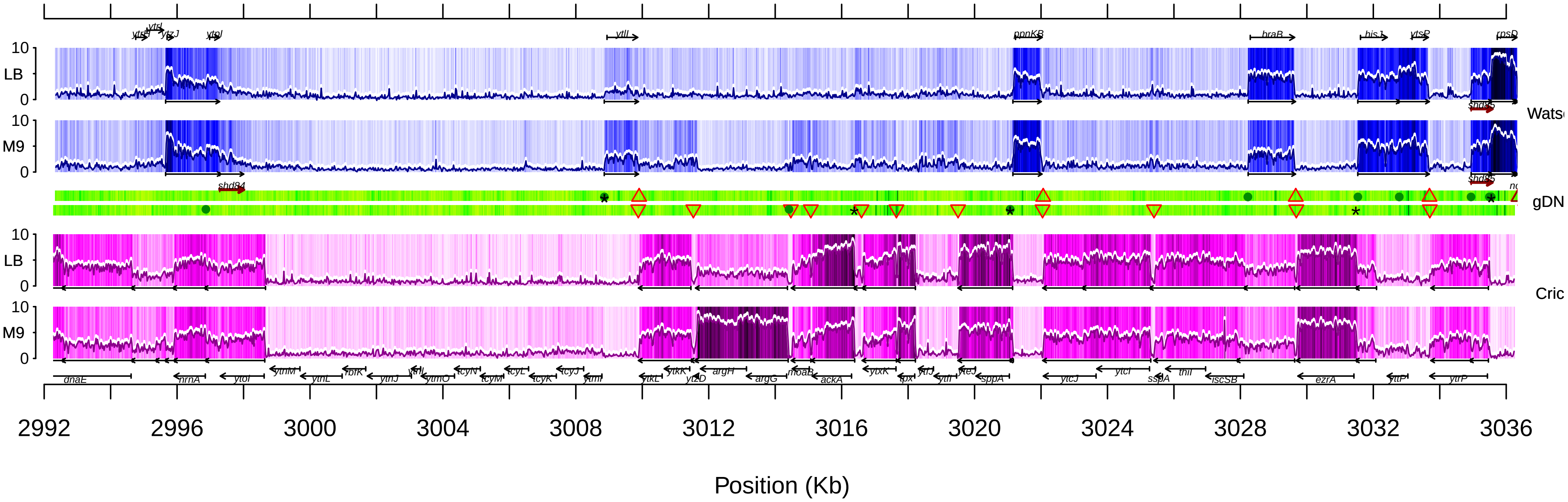

3036 − 3080 Kb

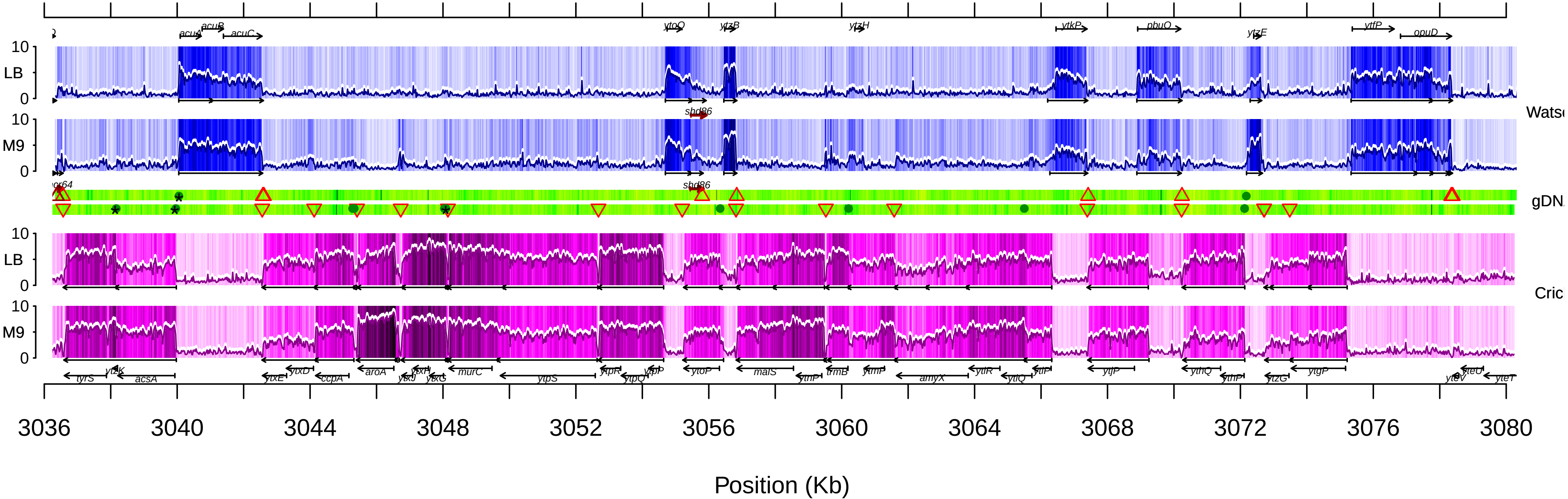

3080 − 3124 Kb

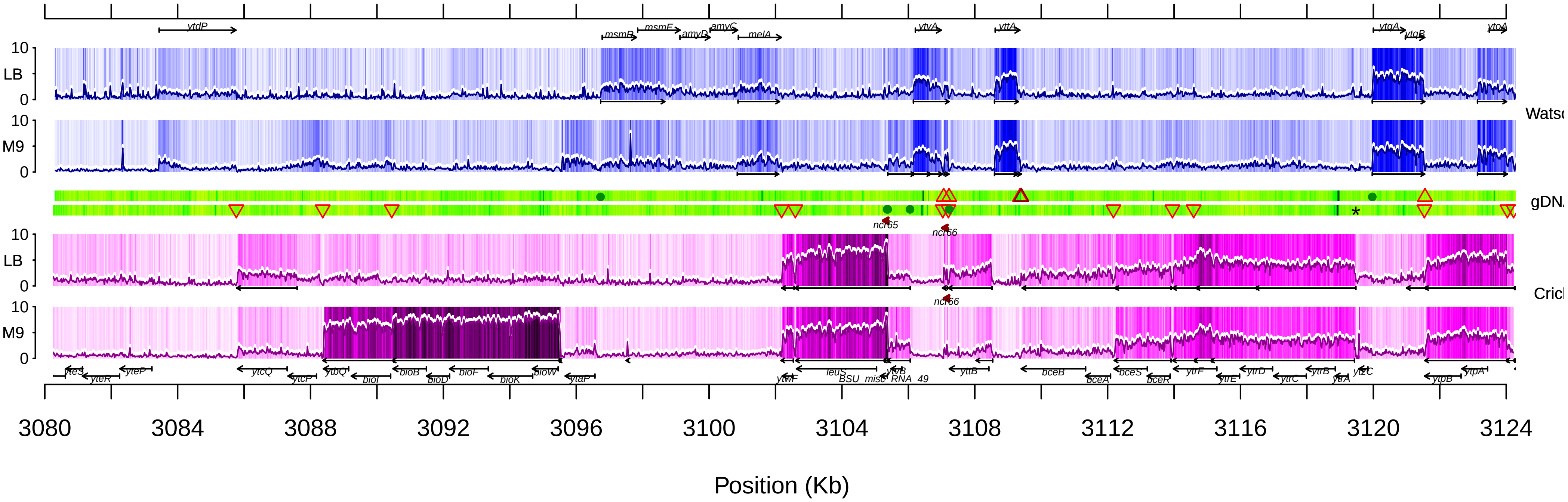

3124 − 3168 Kb

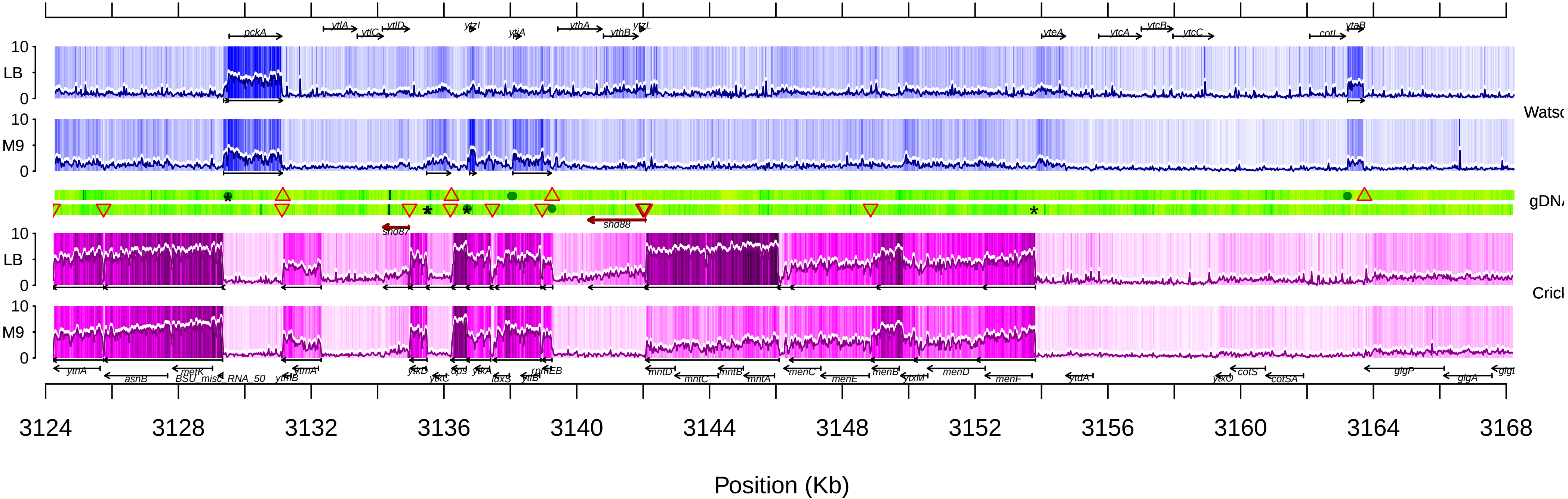

3168 − 3212 Kb

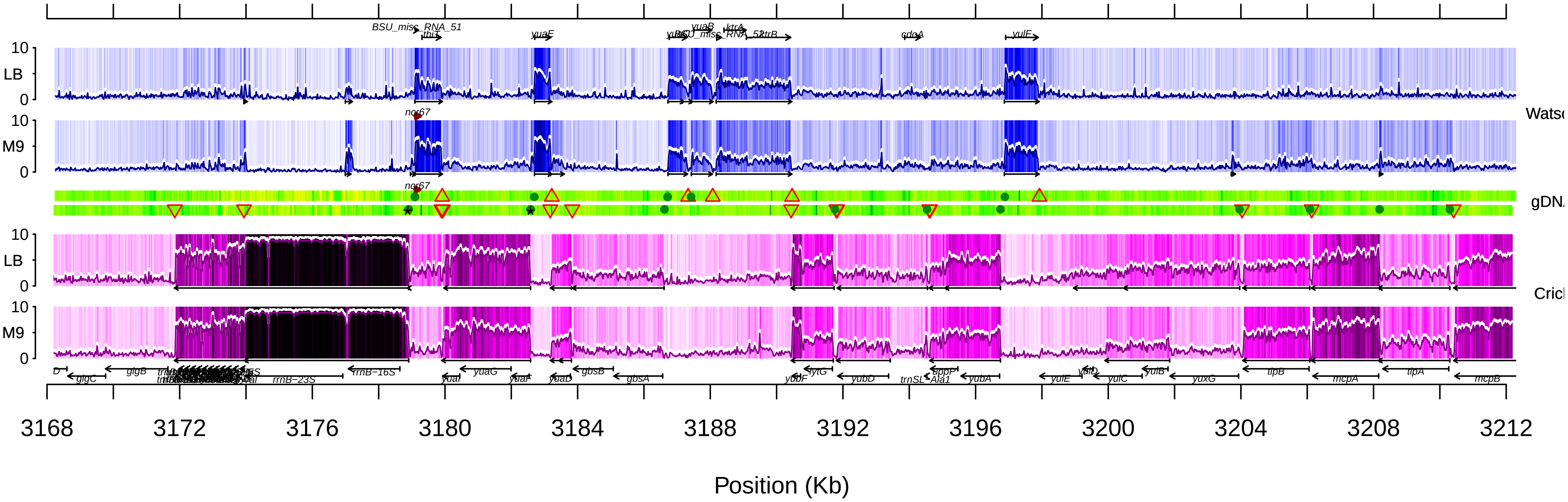

3212 − 3256 Kb

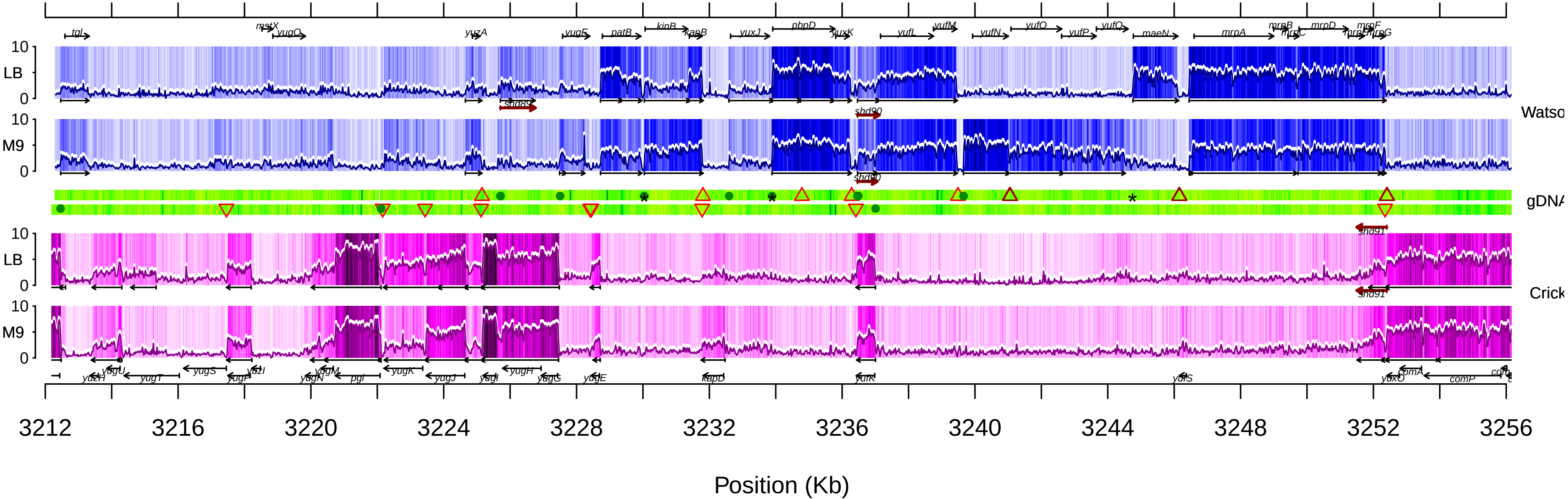

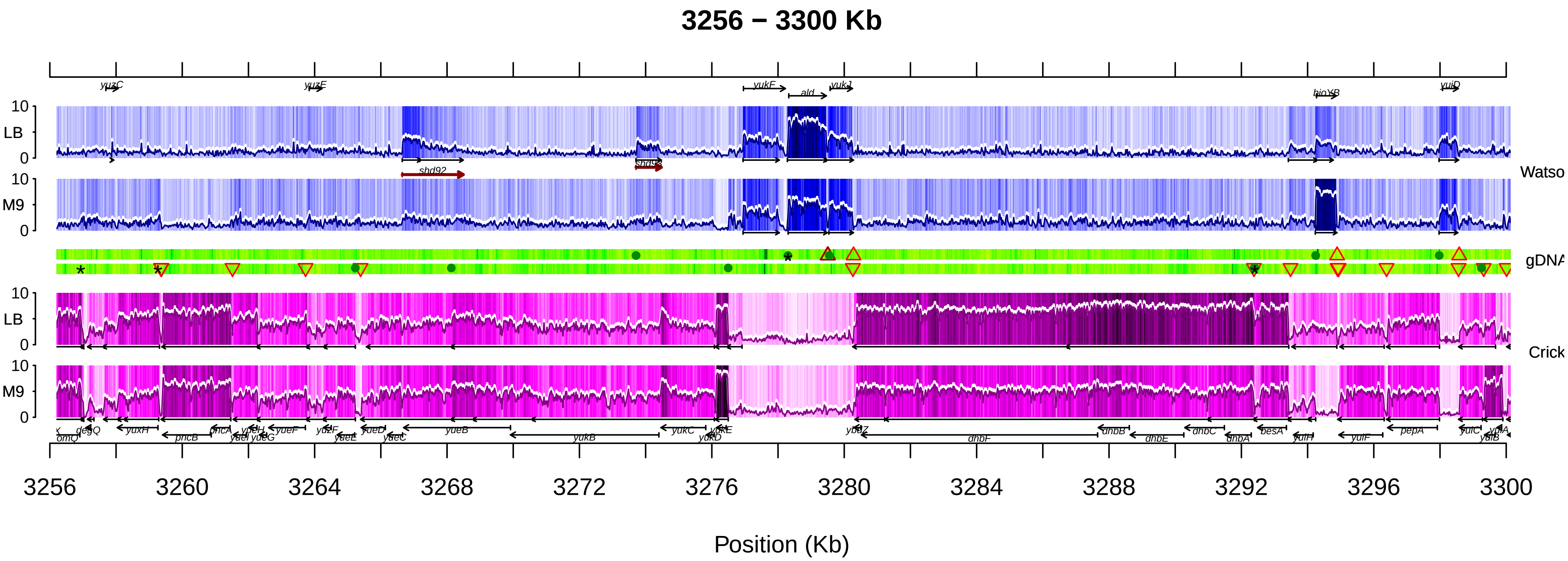
3256 − 3300 Kb
LB
M9
Watso
gDNA
Crick
3256
3260
3264
3268
3272
3276
3280
3284
3288
3292
3296
3300
Position (Kb)

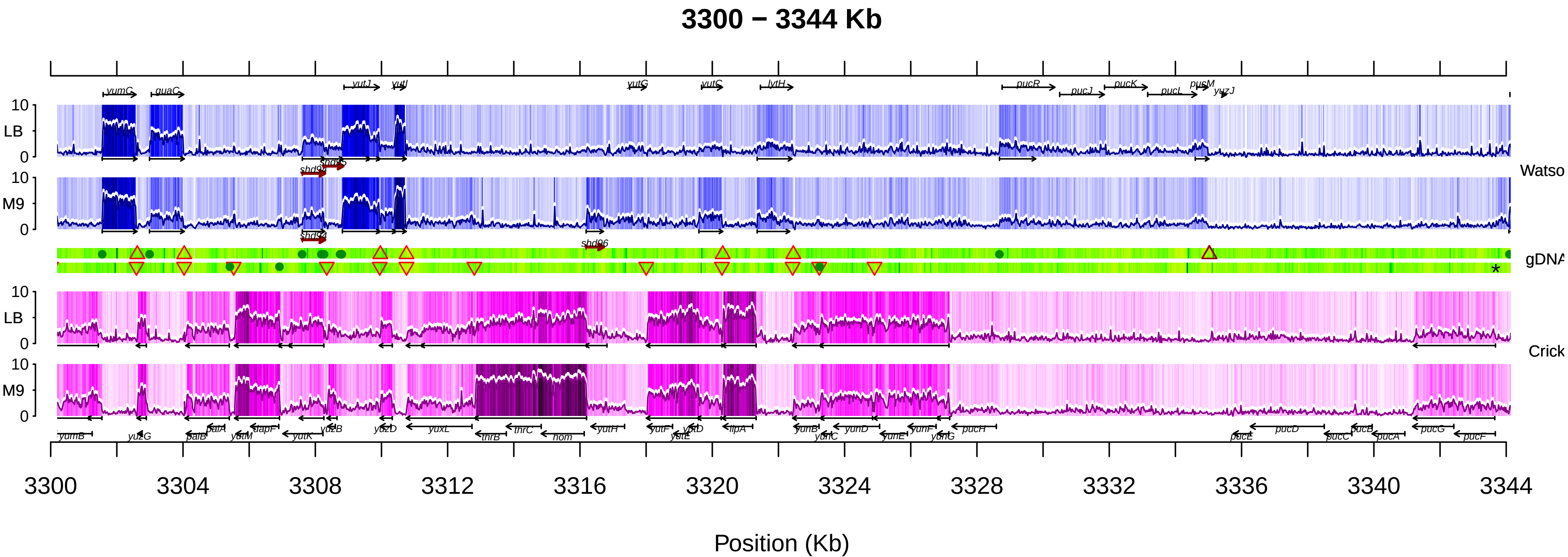
3300 − 3344 Kb
LB
M9
Watso
gDNA
Crick
3300
3304
3308
3312
3316
3320
3324
3328
3332
3336
3340
3344
Position (Kb)

3344 − 3388 Kb

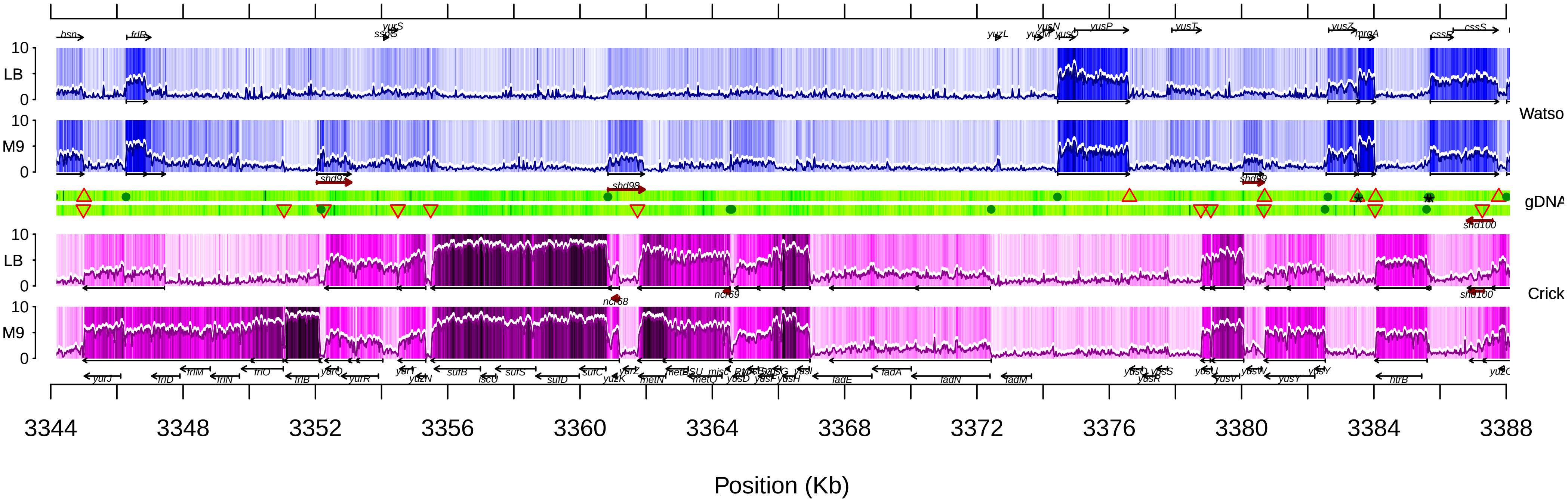

3388 − 3432 Kb

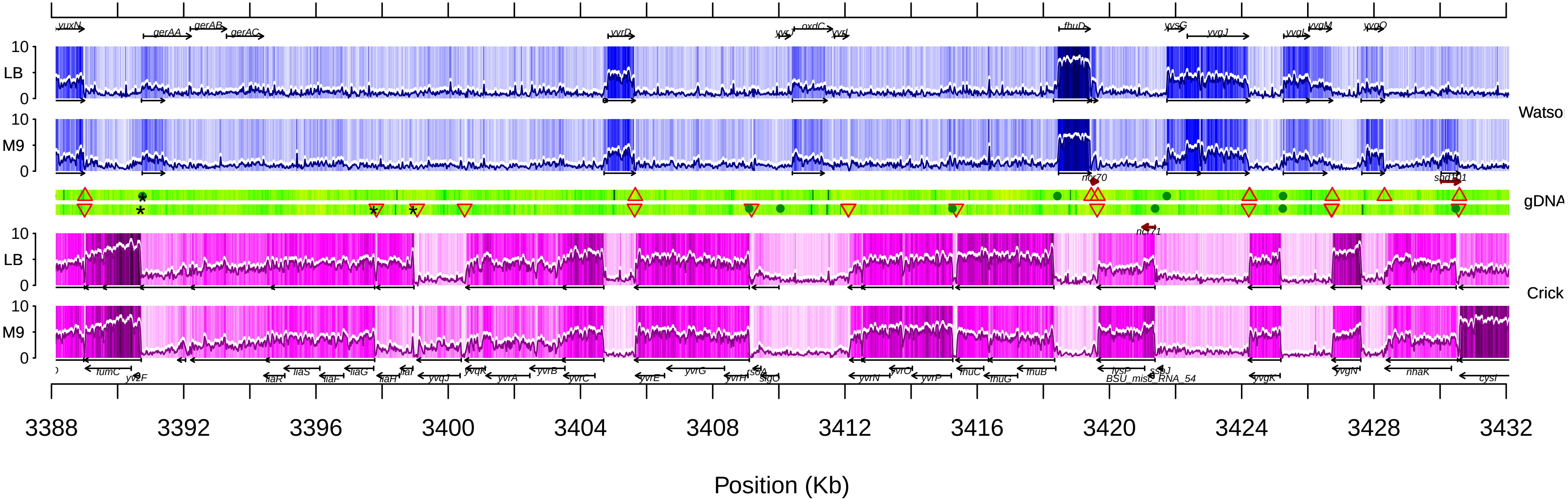

3432 − 3476 Kb

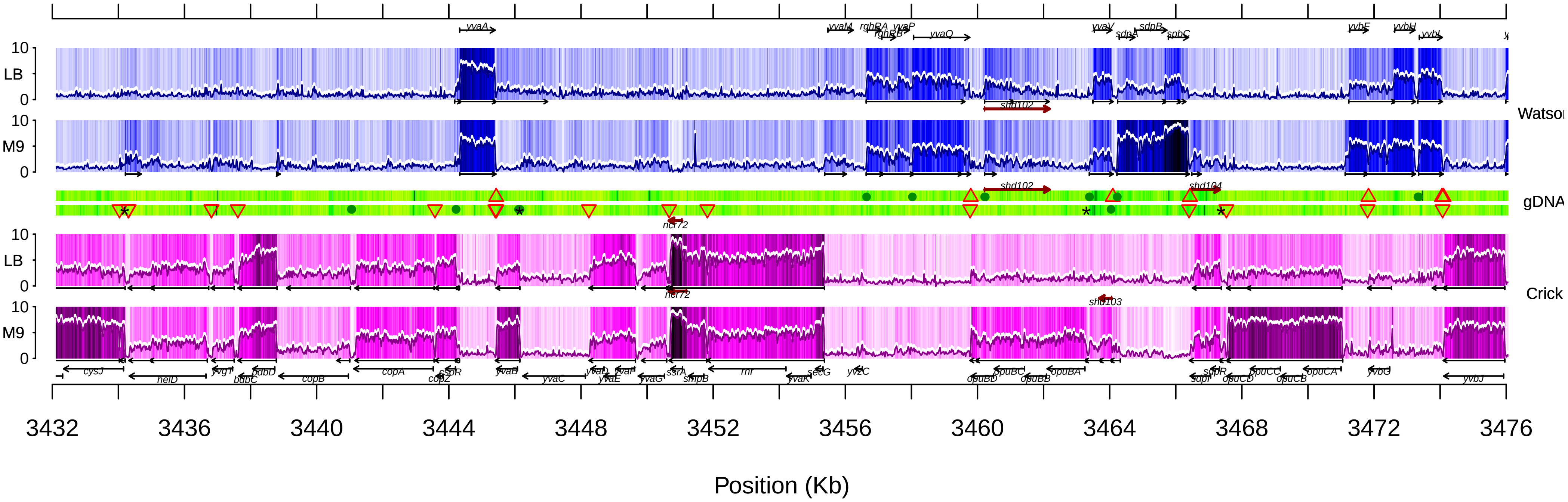

3476 − 3520 Kb

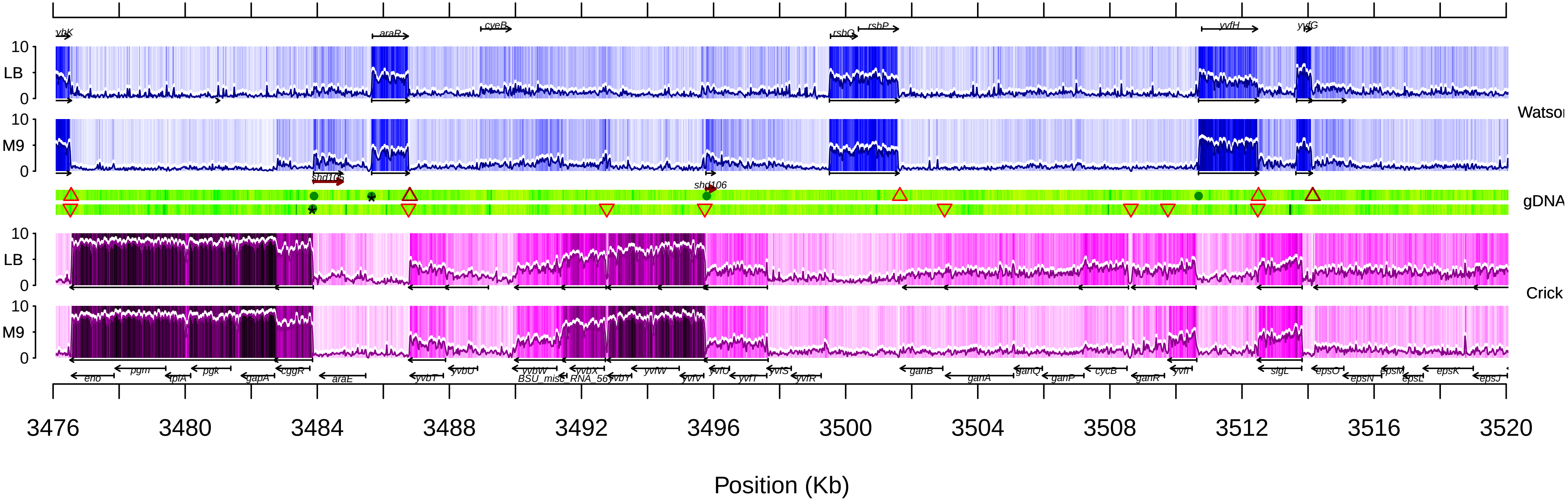

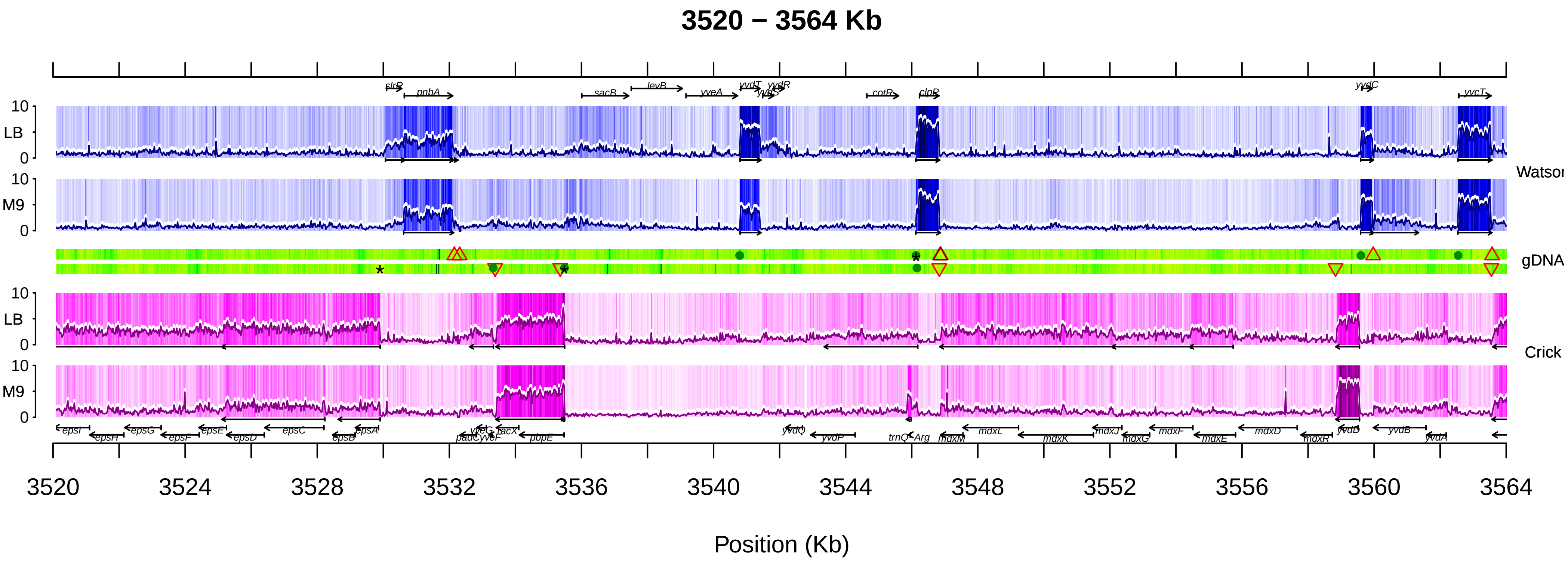
3520 − 3564 Kb
LB
M9
Watson
gDNA
Crick
10
0
3520
3524
3528
3532
3536
3540
3544
3548
3552
3556
3560
3564
Position (Kb)

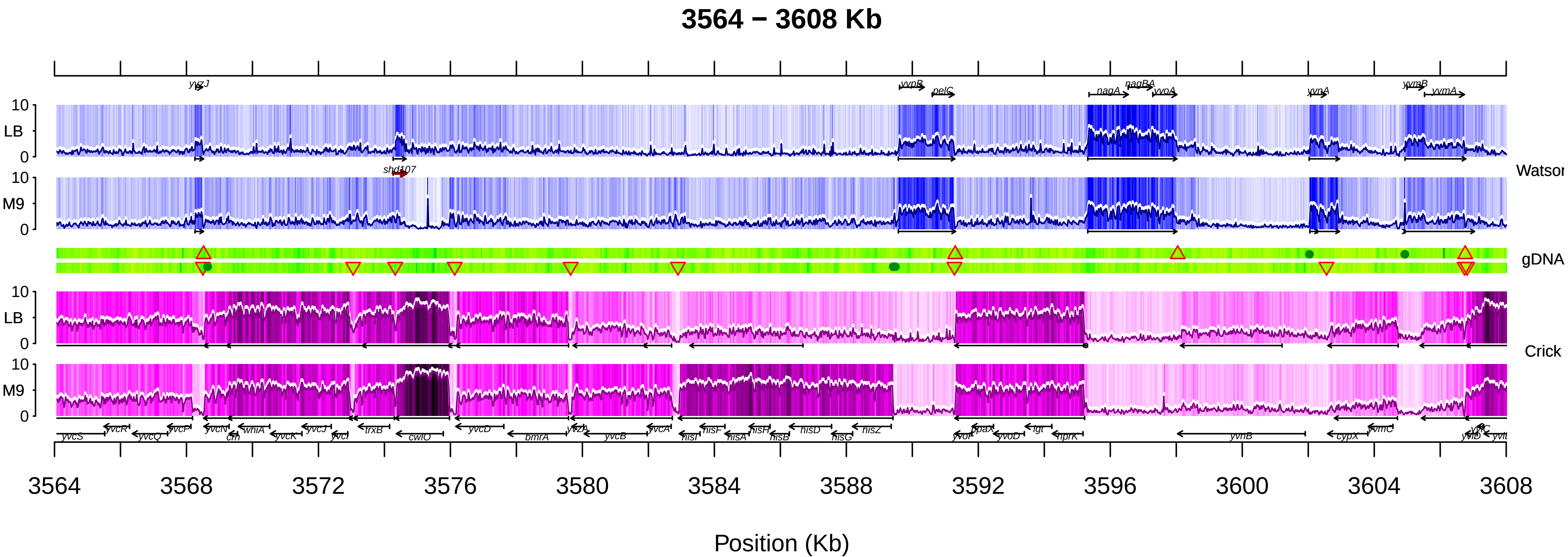
3564 − 3608 Kb
LB
M9
Watson
gDNA
Crick
10
0
shd107
3564
3568
3572
3576
3580
3584
3588
3592
3596
3600
3604
3608
Position (Kb)

3608 − 3652 Kb

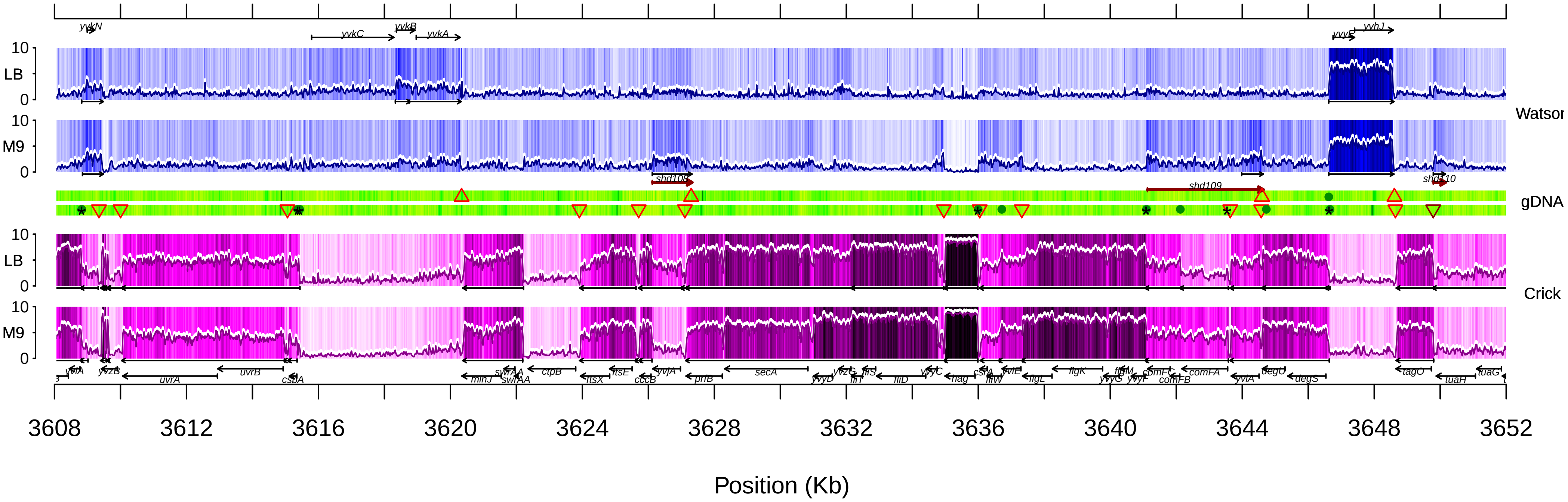

3652 − 3696 Kb

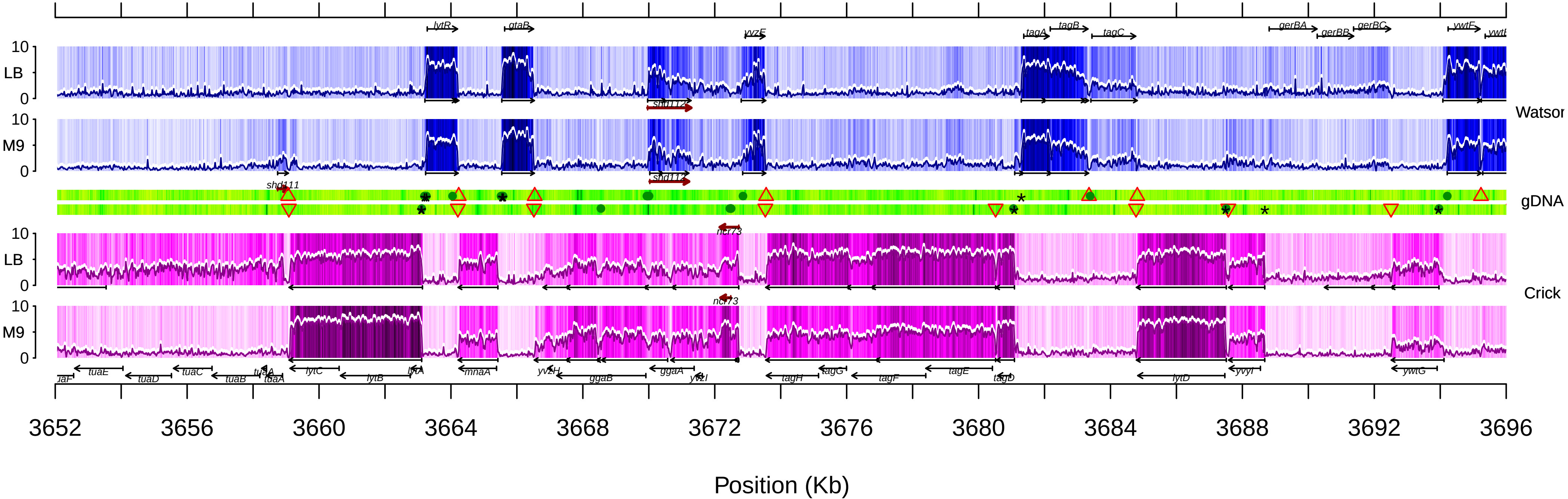

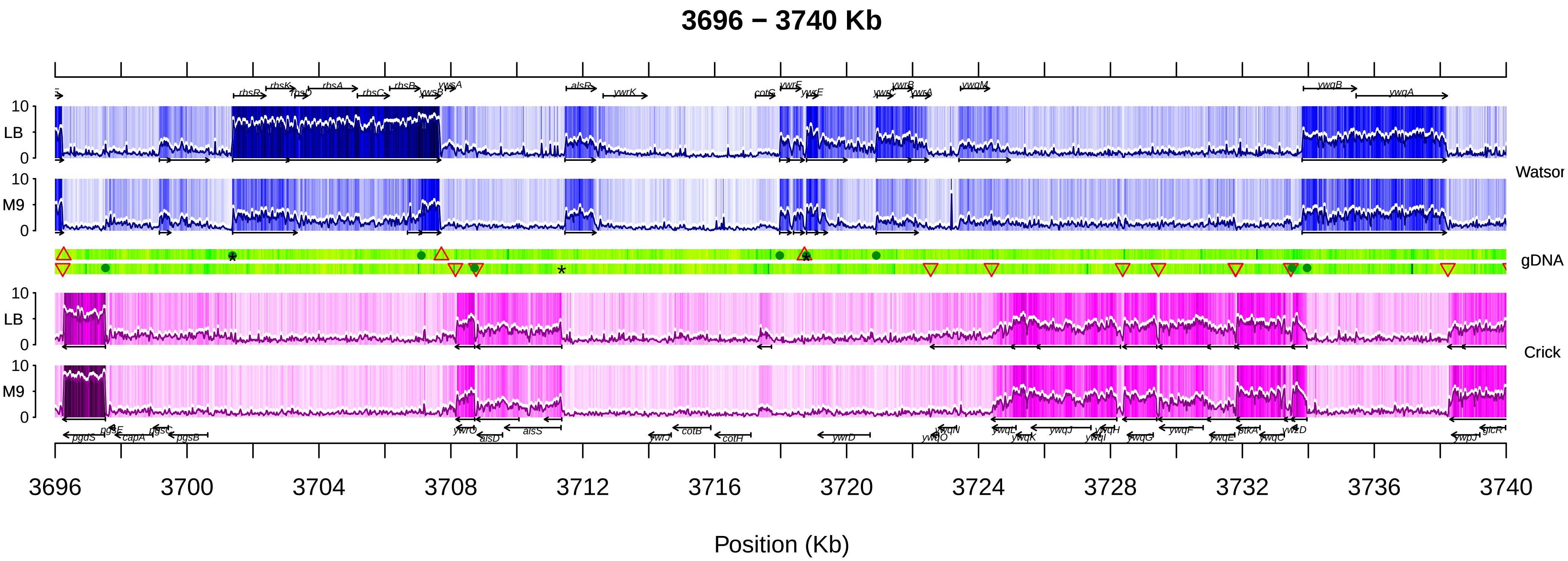
3696 − 3740 Kb
LB
M9
Watson
gDNA
LB
M9
Crick
3696
3700
3704
3708
3712
3716
3720
3724
3728
3732
3736
3740
Position (Kb)

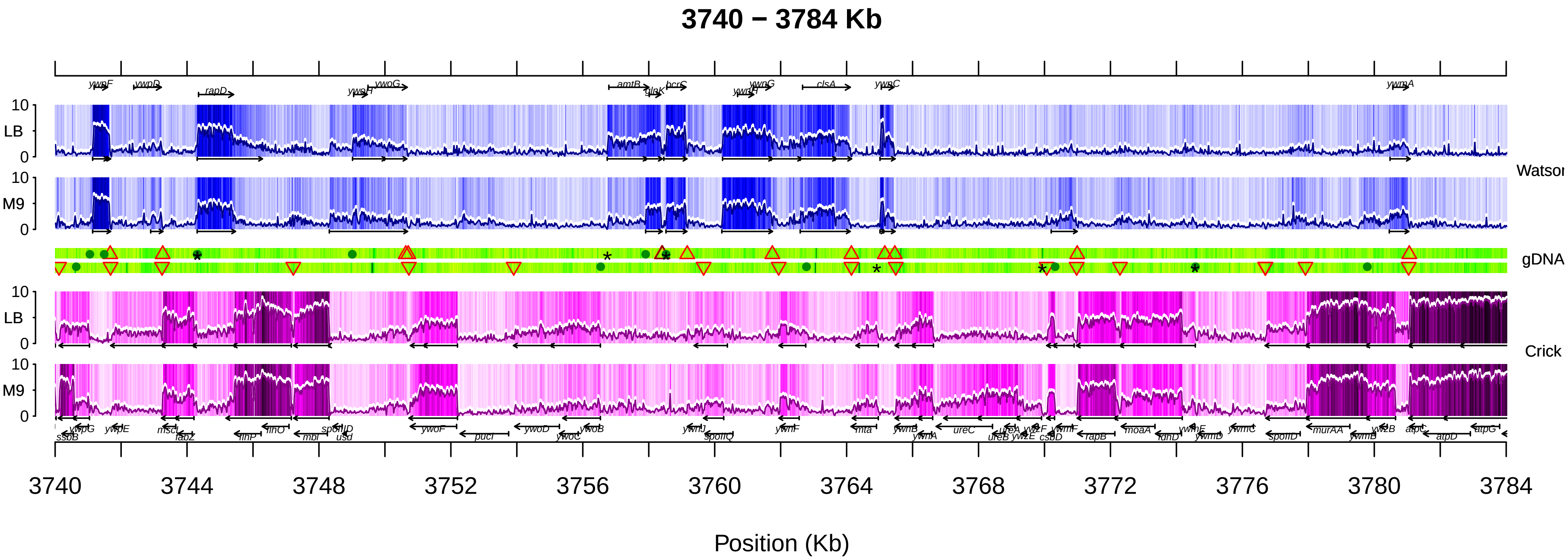
3740 − 3784 Kb
LB
M9
Watson
gDNA
LB
M9
Crick
3740
3744
3748
3752
3756
3760
3764
3768
3772
3776
3780
3784
Position (Kb)

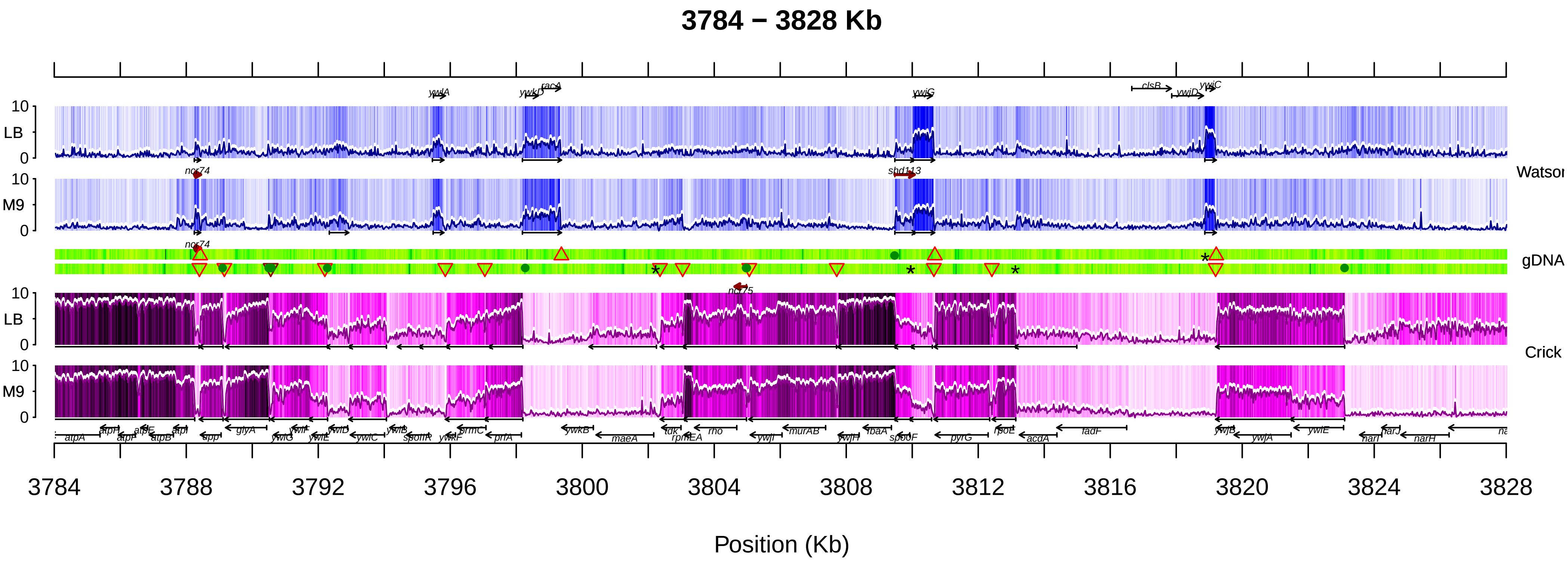
3784 − 3828 Kb
LB
M9
Watson
gDNA
Crick
ncr74
ncr75
shd113
3784
3788
3792
3796
3800
3804
3808
3812
3816
3820
3824
3828
Position (Kb)

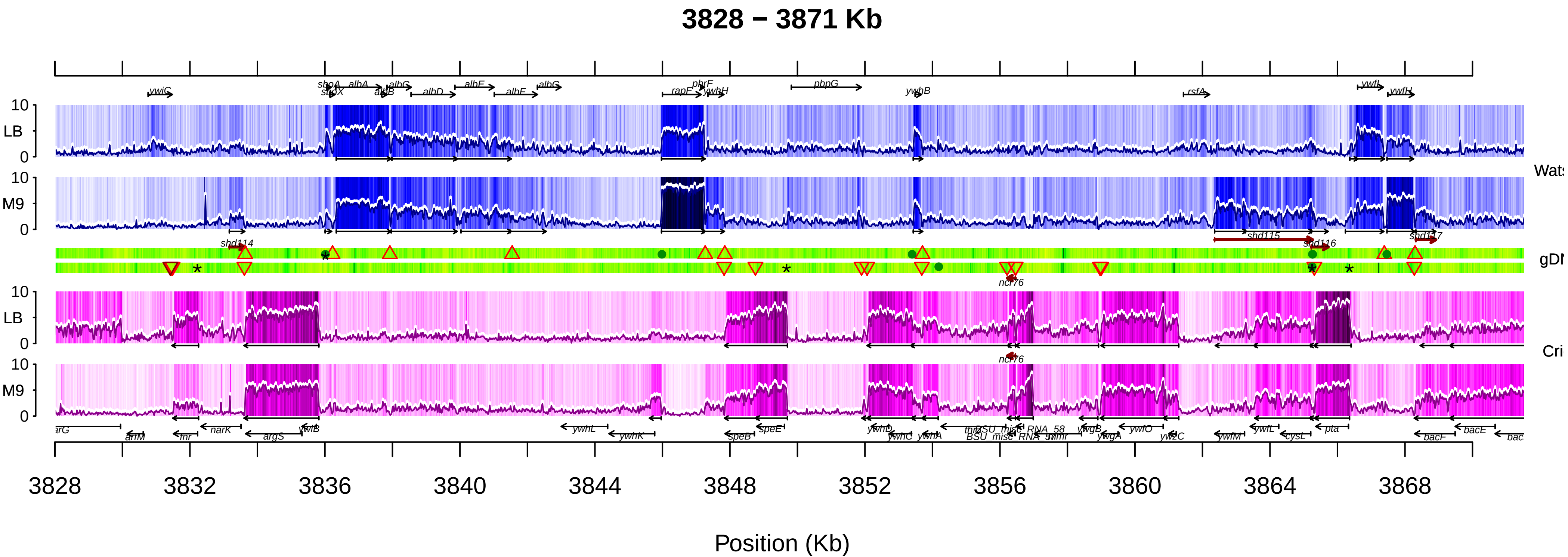
3828 − 3871 Kb
LB
M9
shd114
shd115
shd116
ncr76
3828
3832
3836
3840
3844
3848
3852
3856
3860
3864
3868
Position (Kb)

3872 – 3916 Kb

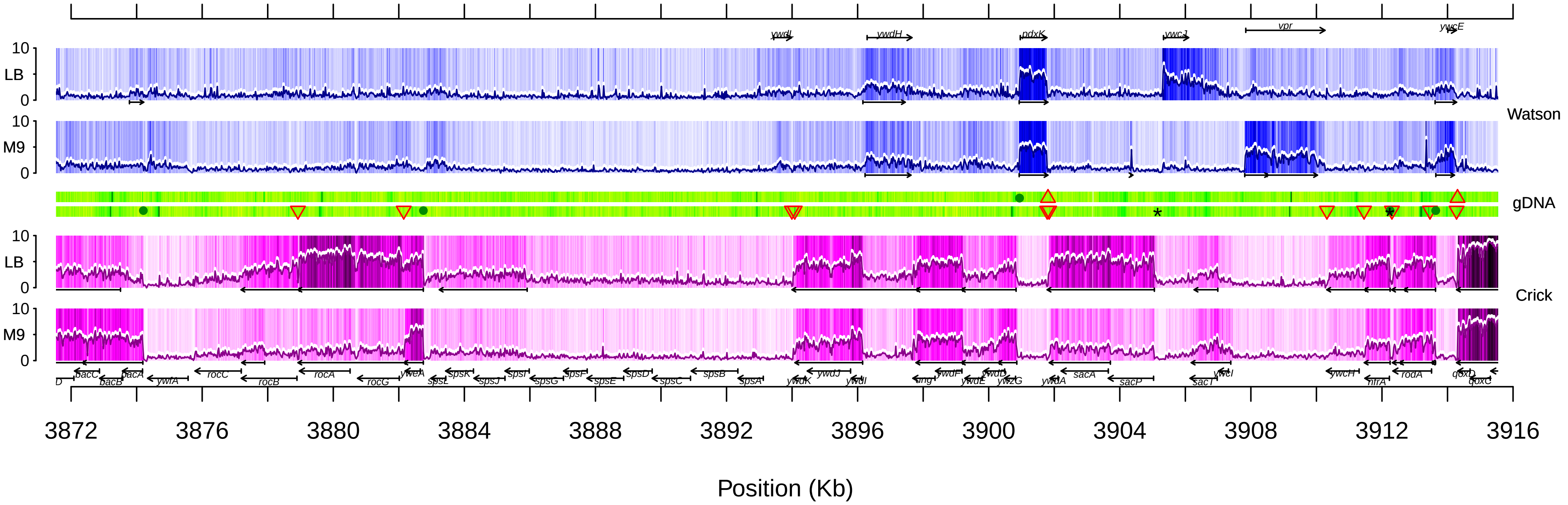

3916 – 3960 Kb

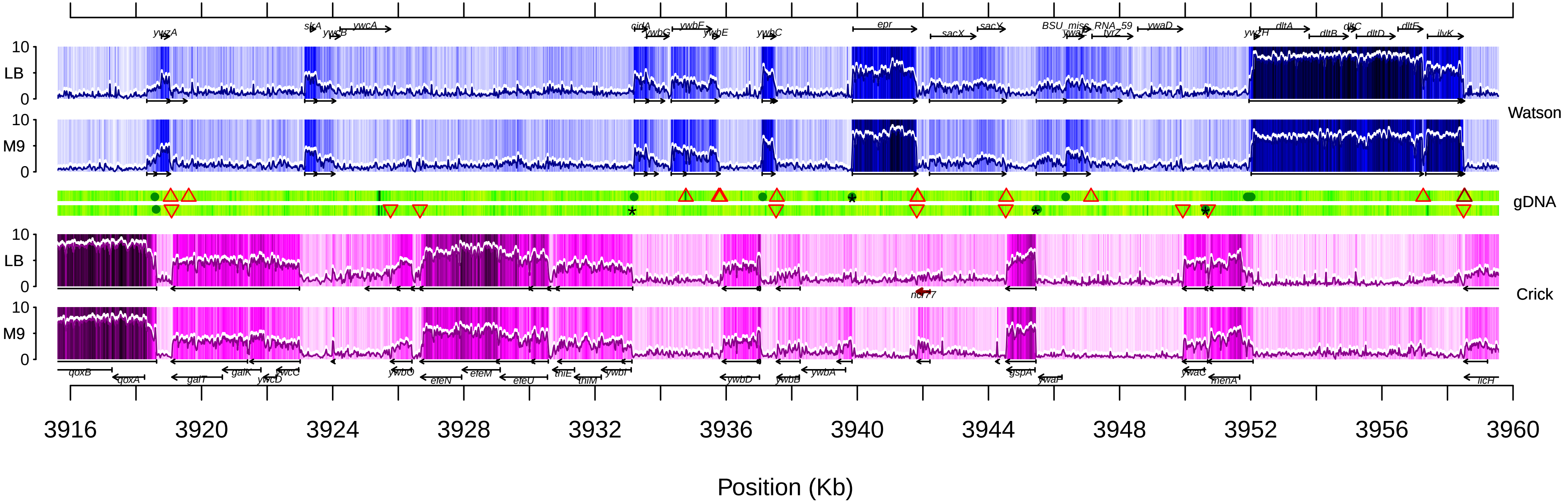

3960 − 4003 Kb

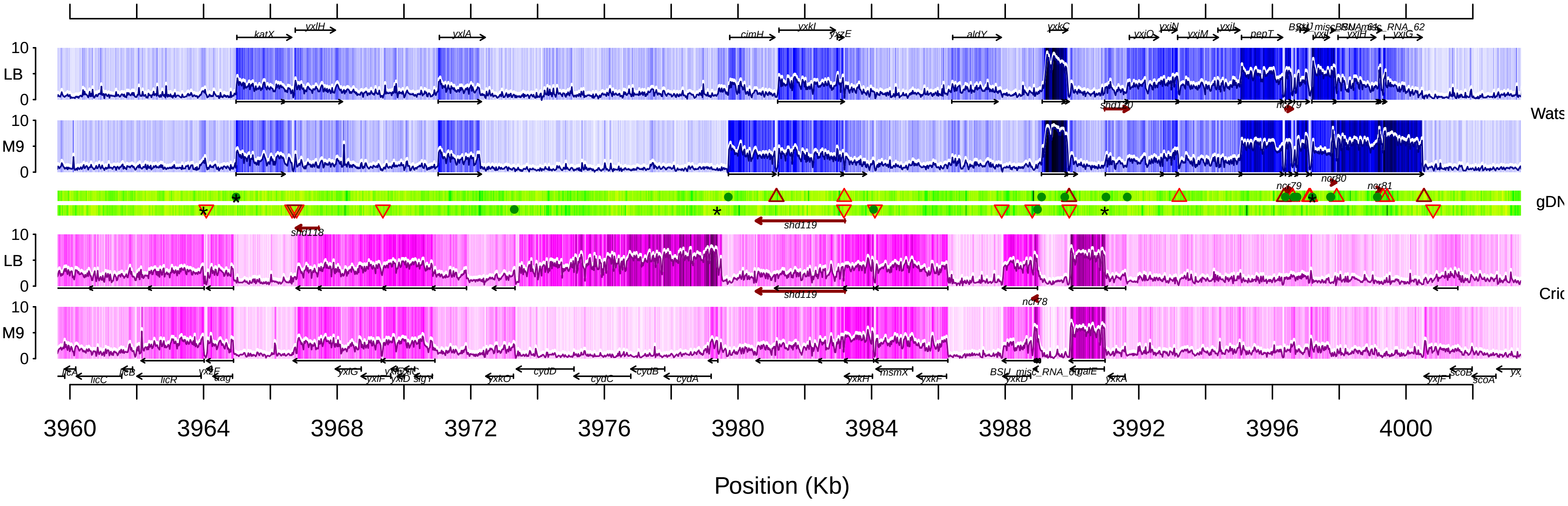

4004 − 4048 Kb

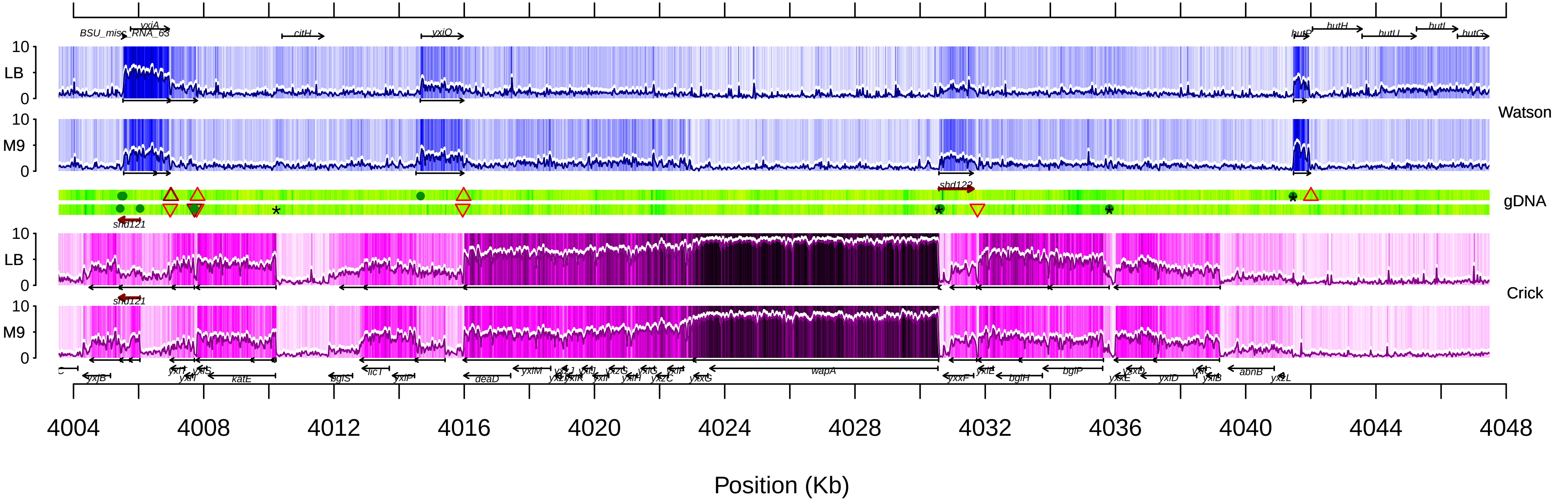

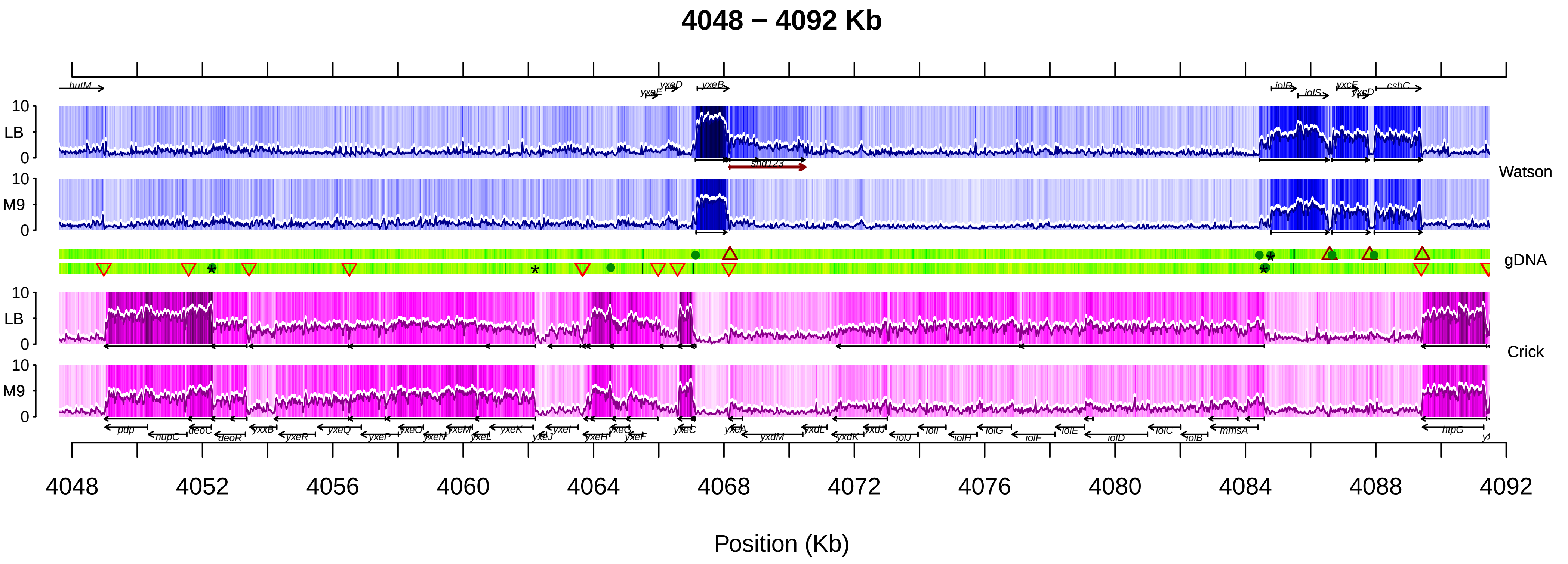
4048 − 4092 Kb
LB
M9
Watson
gDNA
Crick
10
0
butM
yxeD
yxeF
yxeB
iolR
iolS
yxcE
yxcD
csbC
snd123
pdp
nupC
deoC
deoR
yxxB
yxeR
yxeQ
yxeP
yxeO
yxeN
yxeM
yxeL
yxeK
yxeJ
yxeI
yxeH
yxeG
yxeF
yxeC
yxeA
yxdM
yxdL
yxdK
yxdJ
iolJ
iolI
iolH
iolG
iolF
iolE
iolD
iolC
iolB
mmsA
htpG
4048
4052
4056
4060
4064
4068
4072
4076
4080
4084
4088
4092
Position (Kb)

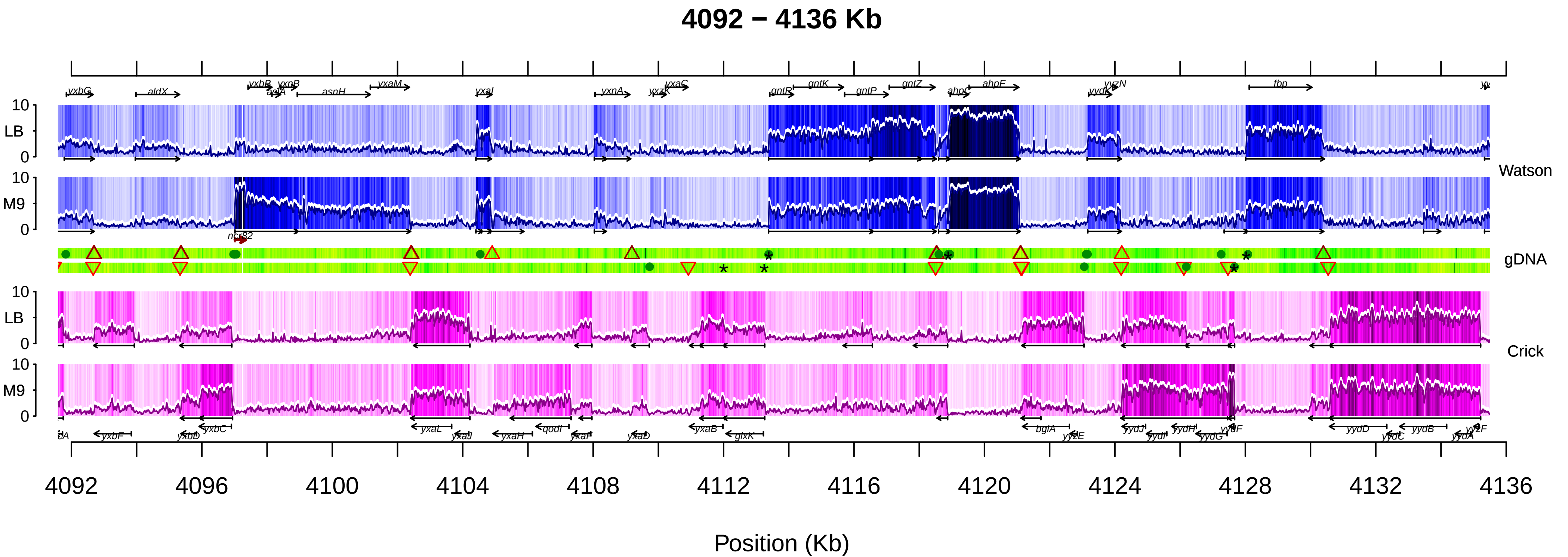
4092 − 4136 Kb
LB
M9
Watson
gDNA
Crick
10
0
yxbG
aldX
yxbB
yxnB
asnH
yxaM
yxaI
yxnA
yxaC
gntR
gntK
gntP
gntZ
ahpC
ahpF
yyzN
yydK
fbp
ncr82
yxbF
yxbD
yxbC
yxaL
yxaJ
yxaH
qodI
yxaF
yxaD
yxaB
glxK
bglA
yyzE
yydJ
yydI
yydH
yydG
yydF
yydD
yydC
yydB
yydA
yyzF
4092
4096
4100
4104
4108
4112
4116
4120
4124
4128
4132
4136
Position (Kb)

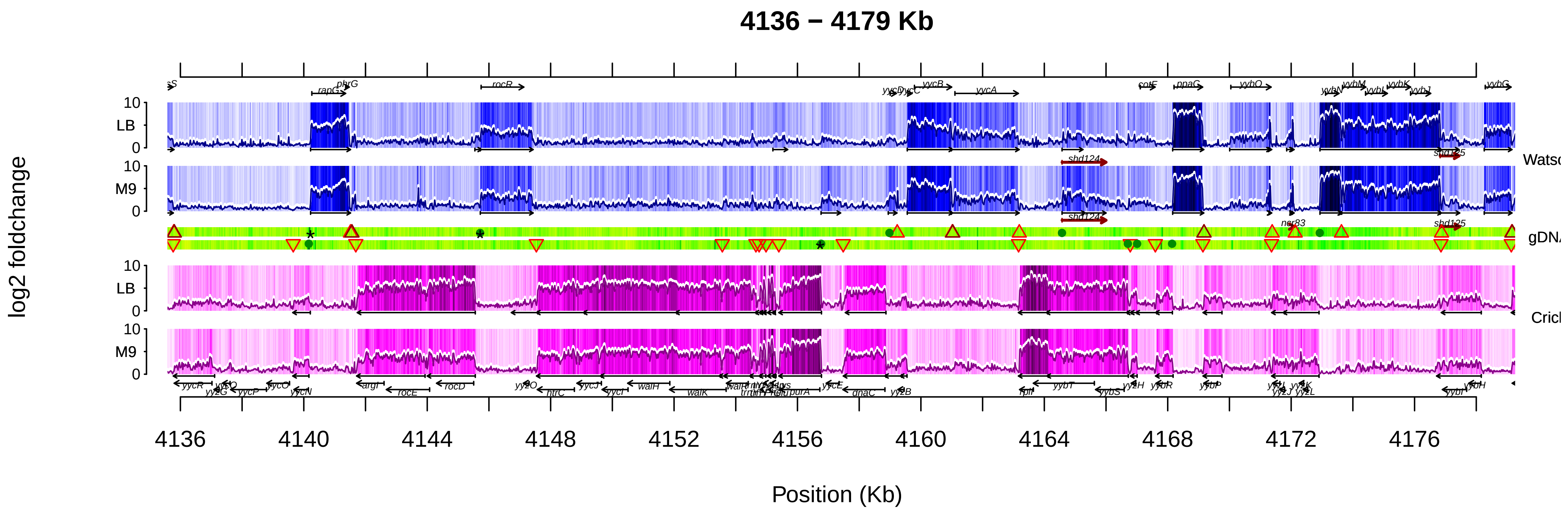
4136 − 4179 Kb
log2 foldchange
LB
M9
Watso
gDNA
Cricl
0
10
4136
4140
4144
4148
4152
4156
4160
4164
4168
4172
4176
Position (Kb)

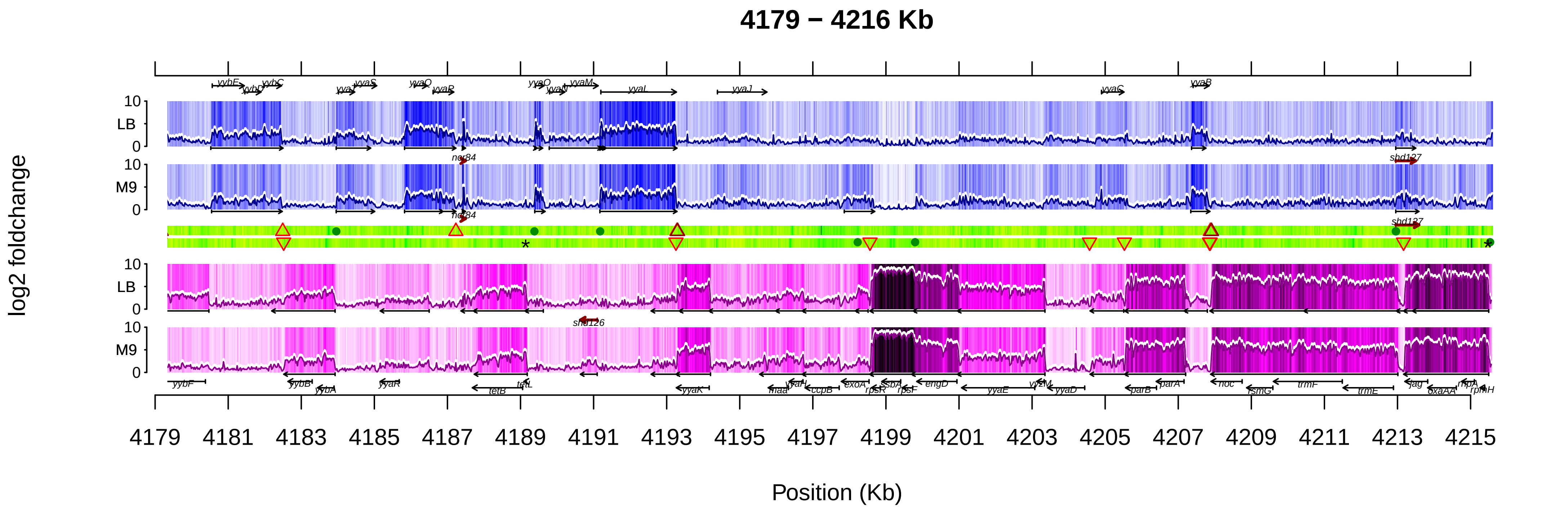
4179 − 4216 Kb
log2 foldchange
LB
M9
0
10
4179
4181
4183
4185
4187
4189
4191
4193
4195
4197
4199
4201
4203
4205
4207
4209
4211
4213
4215
Position (Kb)
